# Supplementary material for: Medulloblastoma group 3 and 4 tumors comprise a clinically and biologically significant expression continuum reflecting human cerebellar development
Source: Cell Rep. 2022 Aug 3;40(5):111162. doi: 10.1016/j.celrep.2022.111162 (PMC9638015; doi:10.1016/j.celrep.2022.111162)
Supplement: Document S1. Figures S1–S7 [file mmc1.pdf]

Cell Reports, Volume *40*

Supplemental information

Medulloblastoma group 3 and 4 tumors comprise a clinically and biologically significant expression continuum reflecting human cerebellar development

Daniel Williamson, Edward C. Schwalbe, Debbie Hicks, Kimberly A. Aldinger, Janet C. Lindsey, Stephen Crosier, Stacey Richardson, Jack Goddard, Rebecca M. Hill, Jemma Castle, Yura Grabovska, James Hacking, Barry Pizer, Stephen B. Wharton, Thomas S. Jacques, Abhijit Joshi, Simon Bailey, and Steven C. Clifford

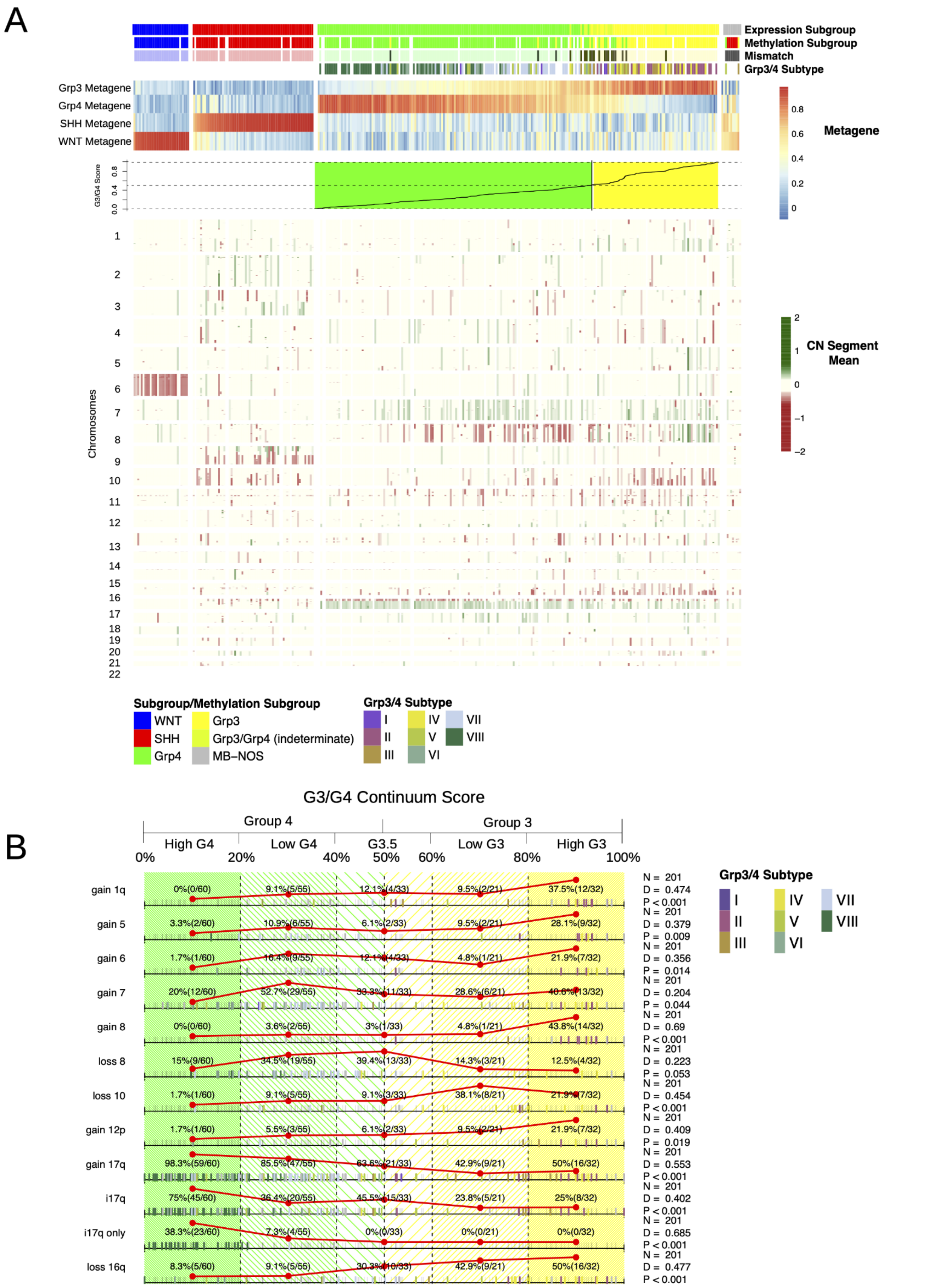

Figure 1S: Copy number changes are non-randomly distributed with respect to position on the Group3/Group4 continuum. A: Heatmap showing copy number changes by chromosome for n=331 MB and grouped by subgroup. MB_{Grp3}/MB_{Grp4} individuals are ordered by G3/G4 score. Column annotation shows subgroup as determined by RNA-seq (Expression Subgroup) as determined by DNA methylation array (Methylation Subgroup), and DNA methylation MB_{Grp3}/MB_{Grp4} subtype (I-VIII) as per Sharma *et al* 2019 (Sharma et al., 2019) as defined using MNPv2 classifier (Capper et al., 2018) (Grp3/4 Subtype). The line plot (bottom) shows G3/G4 score. **B:** Rug plot showing distribution of aneuploidy/copy number change with respect to G3/G4 score. Summary counts are given according to the convenient divisions of HighG4, LowG4, G3.5, LowG3,

HighG3 (these categories are arbitrary divisions of the continuum for the purposes of visualization and comparison and do not represent “real” subgroups) and reflected by the red line plots. Presence of a given feature is indicated by a bold tick mark, the color of which indicates methylation MB_{Grp3}/MB_{Grp4} subtype (I-VIII). Adjusted P-values for a Kolmogorov-Smirnoff statistic (D) are shown to denote non-random distribution of features with respect to G3/G4 score. Related to Figure 2.

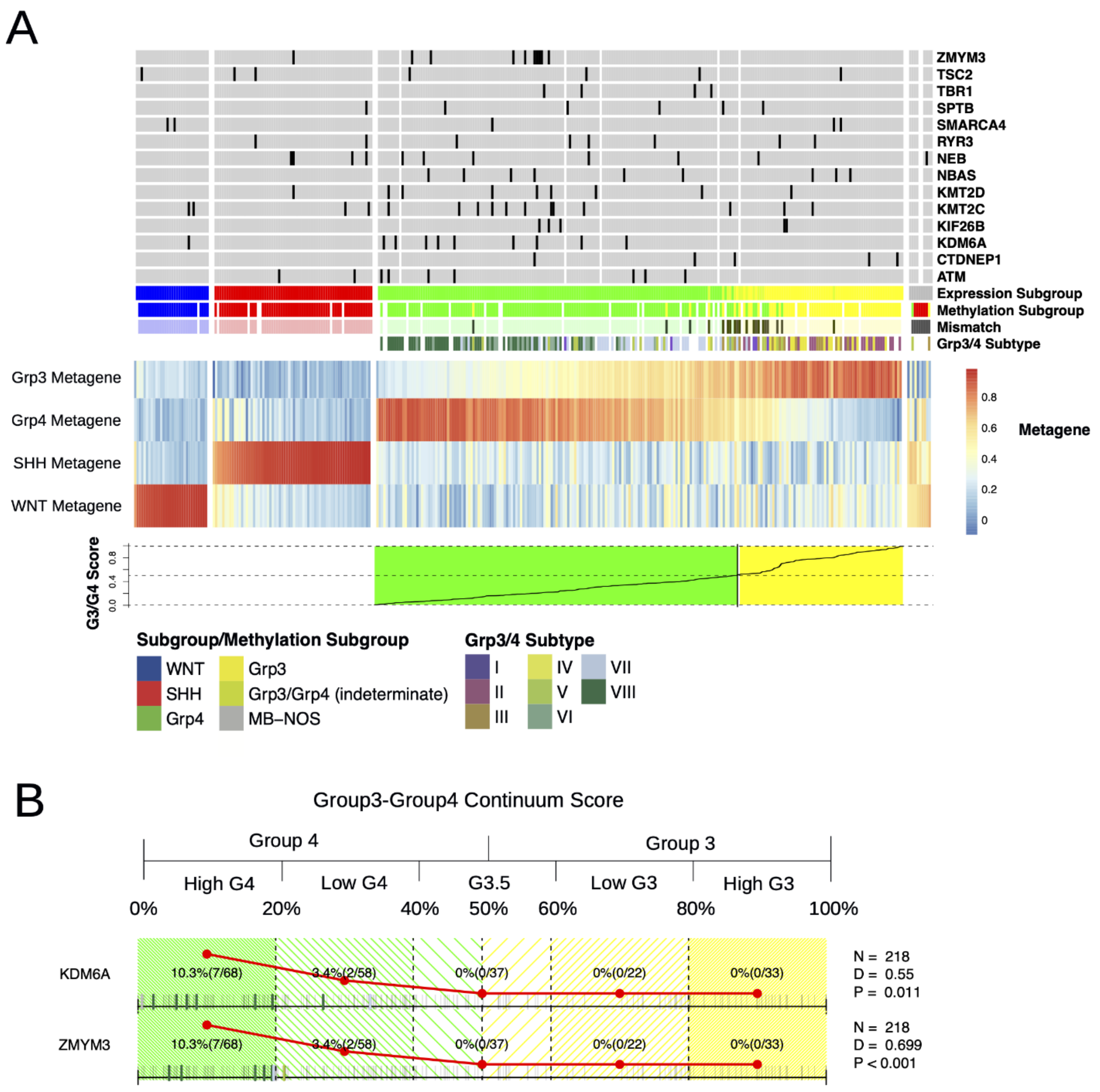

Figure 2S: Certain mutations are non-randomly distributed with respect to the Group3/Group4 continuum. A: Heatmap showing 4 consensus NMF metagenes calculated for n=331 MB and grouped by subgroup. MB_{Grp3}/MB_{Grp4} individuals are ordered by G3/G4 score. Column annotation shows subgroup as determined by RNA-seq (Expression Subgroup) as determined by methylation (Methylation Subgroup), methylation MB_{Grp3}/MB_{Grp4} subtype (I-VIII) as per Sharma *et al* 2019 as defined using MNPv2 classifier (Grp3/4 Subtype). Presence of mutations are indicated to be present or not by dark grey shading. White indicates missing data. **B:** Rug plot showing distribution of mutations with respect to G3/G4 score. Summary counts are given according to the convenient divisions of HighG4, LowG4, G3.5, LowG3, HighG3 (these categories are arbitrary divisions of the continuum for the purposes of visualization and comparison and do not represent “real” subgroups) and reflected by the red line plots. Presence of a given feature is indicated by a bold tick mark the color of which indicates methylation MB_{Grp3}/MB_{Grp4} subtype (I-VIII). P-values for a Kolmogorov-Smirnoff statistic (D) are shown to denote non-random distribution of features with respect to G3/G4 score. Related to Figures 1 & 2.

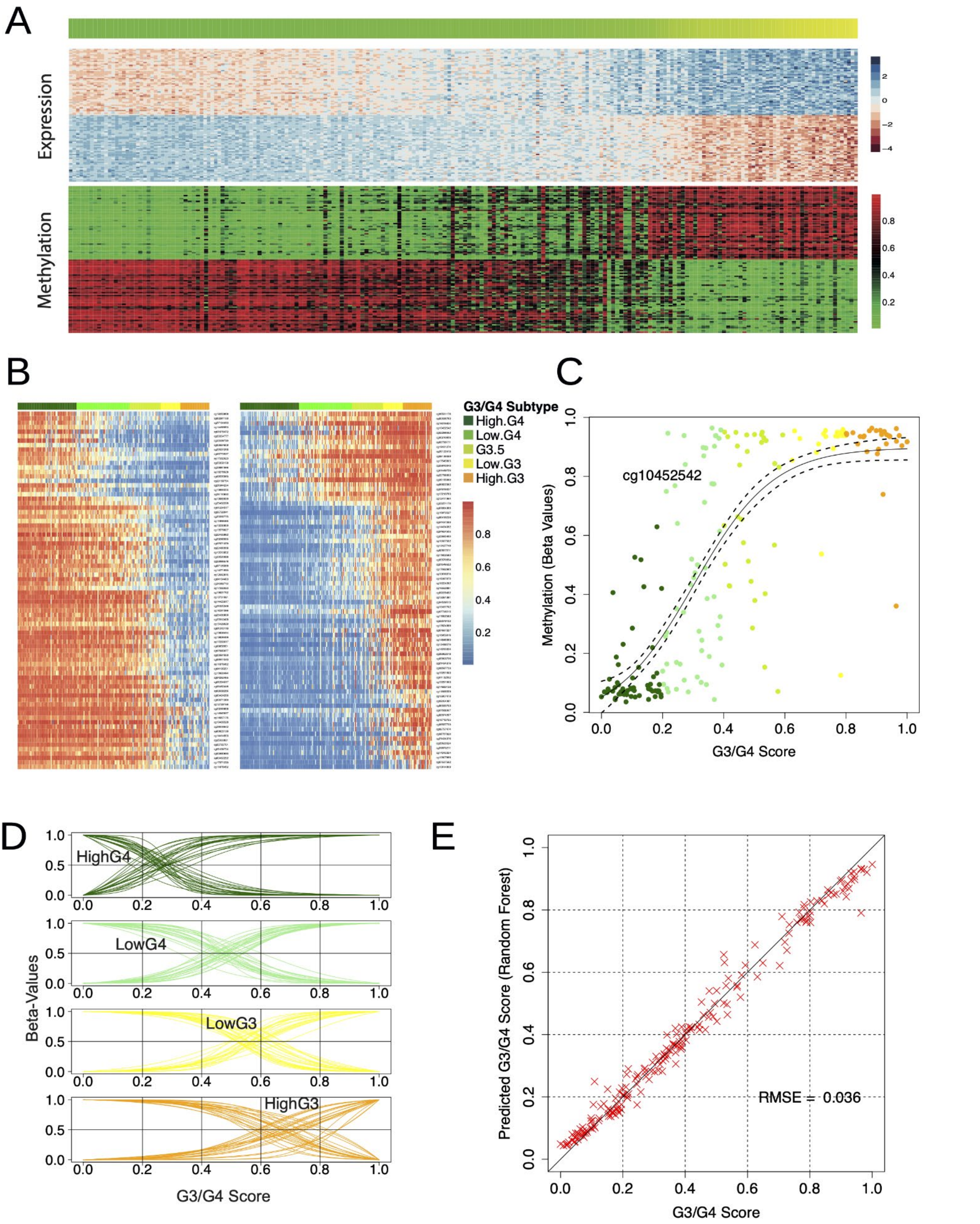

Figure 3S: The transcriptional G3/G4 score can be recapitulated using DNA methylation profiles. A: Heatmap showing top 50 genes most significantly differentially expressed (top) and top 50 CpGs differentially methylated between MB_{Grp3} and MB_{Grp4}. Samples are ordered according to G3/G4 score. Note the difference in gradation for the expression values as opposed to the more binary distribution of DNA methylation beta-values. **B:** Heatmap showing DNA methylation values of the top 40 most discriminatory CpGs distinguishing HighG4 (dark green), LowG4 (light green), Low G3 (yellow) and High G3 (orange). G4 hypermethylated CpGs are shown on the left and hypomethylated CpGs on the right. Samples are ordered according to G3/G4 score and G3/G4 categories (HighG4, LowG4, G3.5, LowG3, HighG3; these categories are arbitrary divisions of the continuum for the purposes of visualization and comparison and do not represent "real" subgroups) are annotated. **C:** Scatterplot showing beta-values for CpG "cg19784198" colored by G3/G4 categories (HighG4, LowG4, G3.5, LowG3, HighG3) an example of a CpG

differentially expressed between MB_{Grp3} and MB_{Grp4} showing a bimodal methylation distribution. The relationship with G3/G4 score can effectively be modelled by a sigmoid/logistic function. **D:** Fitted sigmoid curve representing the relationship between CpG beta-value and G3/G4 Score. Top 40 most discriminatory CpGs distinguishing HighG4 (dark green), LowG4 (light green), Low G3 (yellow) and High G3 (orange) are shown. **E:** The performance of the cross-validated random forest classifier showing predicted G3/G4 score (derived from DNA methylation values) against actual G3/G4 score (derived from RNA-seq) n = 192. Related to Figure 2.

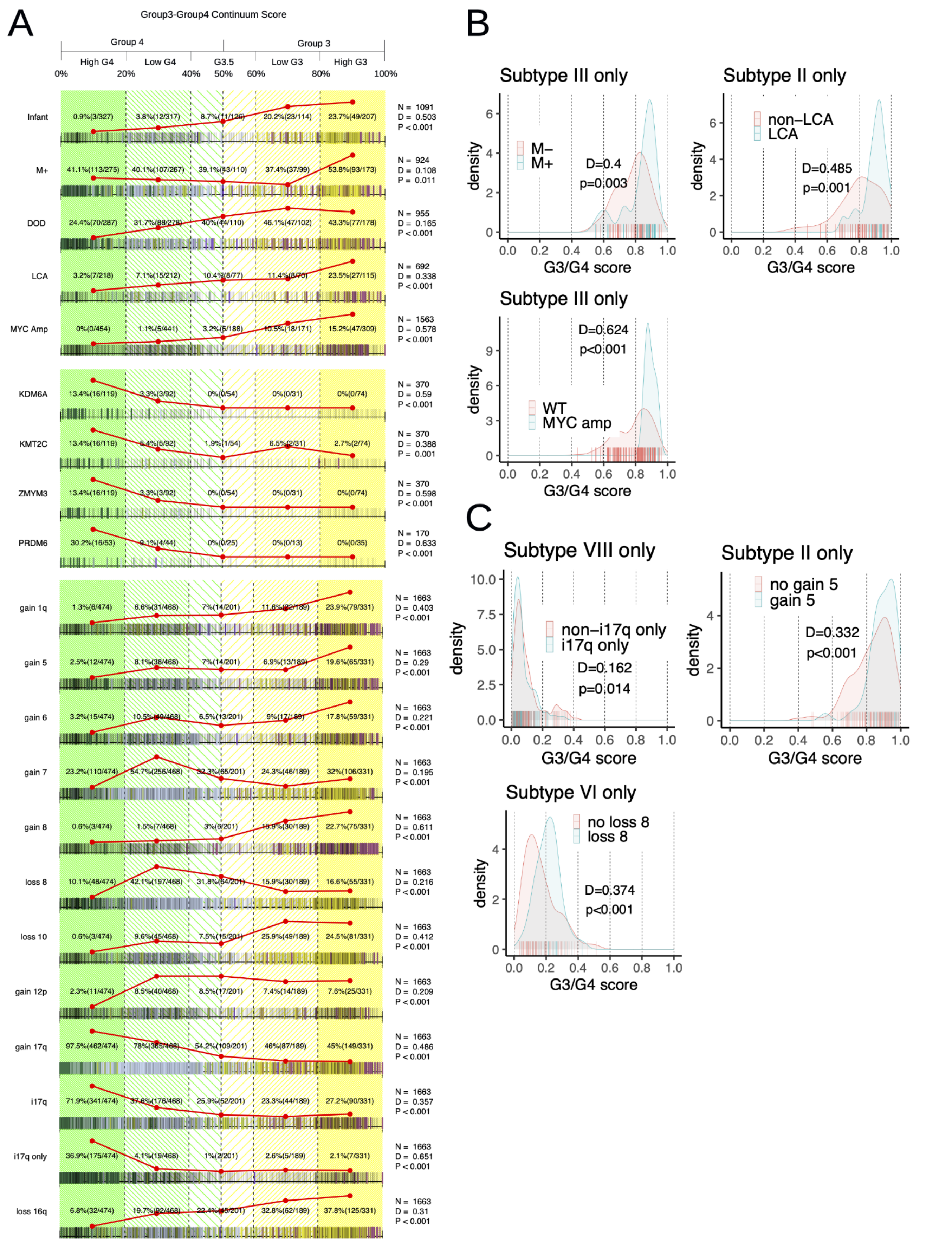

Figure 4S Clinico-pathological characteristics, mutations and copy number changes are all non-randomly distributed with respect to the Group3/Group4 continuum (as determined by DNA-methylation profile). A: Rug plot showing distribution of clinicopath features (top) mutations (middle) and copy number (bottom) with respect to G3/G4 score derived from DNA methylation data. Summary counts are given according to the convenient divisions of HighG4, LowG4, G3.5, LowG3, HighG3 (these categories are arbitrary divisions of the continuum for the purposes of visualization and comparison and do not represent "real" subgroups) and reflected by the red line plots. Presence of a given feature is indicated by a bold tick mark the color of which indicates methylation MB_{Grp3}/MB_{Grp4} subtype (I-VIII). P-values for a Kolmogorov-Smirnoff statistic (D) are shown to denote non-random distribution of features with respect to G3/G4 score. Infant=age at diagnosis < 3 years, Metastases = M+, DOD=Dead of Disease, LCA = Large Cell Anaplasia, PRDM6 = PRDM6 rearrangement. **B:** Empirical density and rug plots

showing the distribution of M+ in MB_{Grp3}/MB_{Grp4} subtype III, LCA in MB_{Grp3}/MB_{Grp4} subtype II and MYC amplification in MB_{Grp3}/MB_{Grp4} subtype III with respect to G3/G4 score. The given clinico-pathological features are significantly non randomly distributed with respect to G3/G4 score even within specific MB_{Grp3}/MB_{Grp4} subtypes as shown by Kolmogorov-Smirnoff test (D). **C:** Empirical density and rug plots showing the distribution of copy number changes i17q in MB_{Grp3}/MB_{Grp4} subtype VIII, Gain of chromosome 5 in MB_{Grp3}/MB_{Grp4} subtype II and loss of chromosome 8 in MB_{Grp3}/MB_{Grp4} subtype VI with respect to G3/G4 score. The given copy number features are significantly non randomly distributed with respect to G3/G4 score even within specific MB_{Grp3}/MB_{Grp4} subtypes as shown by Kolmogorov-Smirnoff test (D). Related to Figure 2.

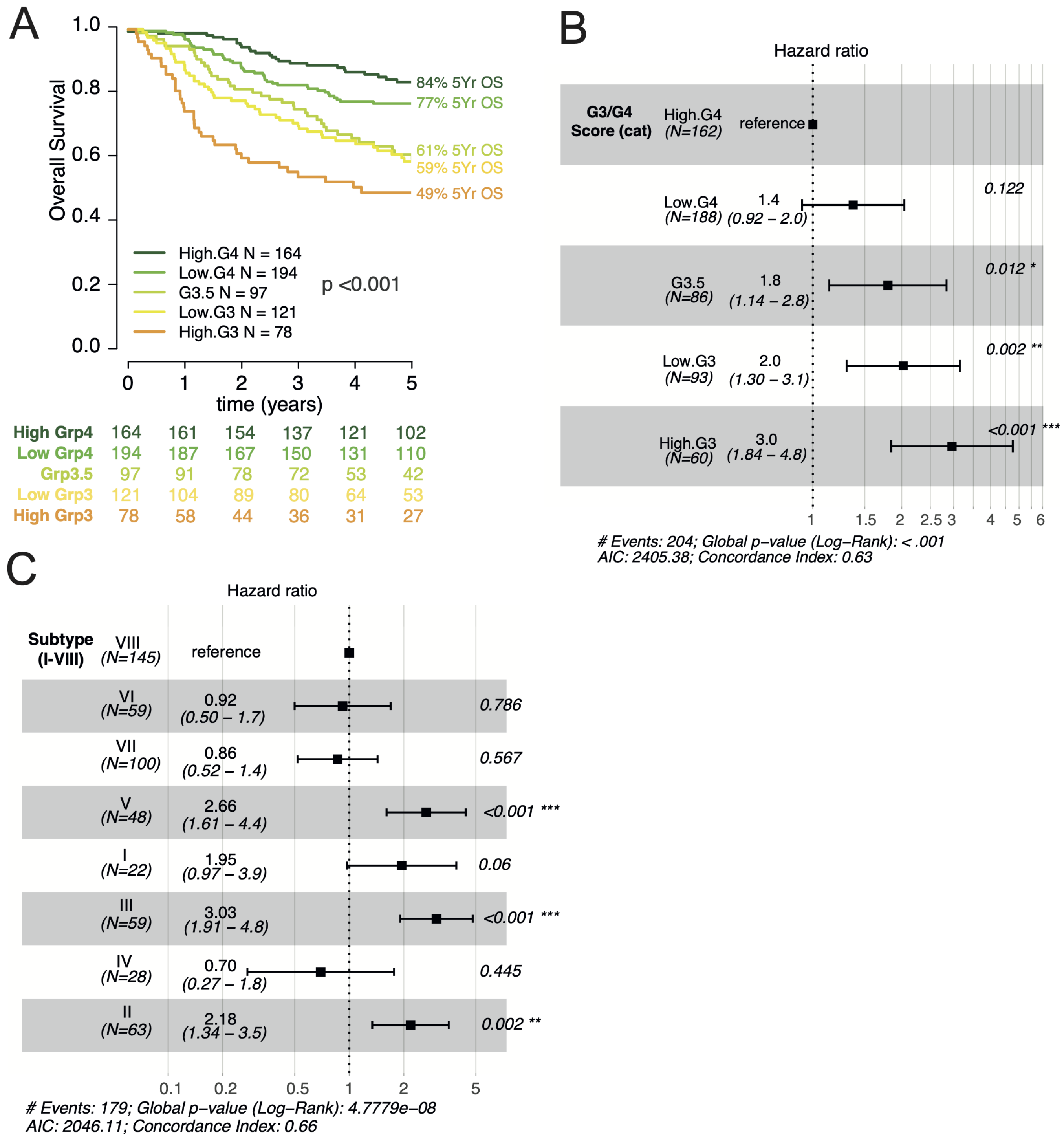

Figure 5S Survival outcomes of Group3/Group4 medulloblastoma patients is significantly related to position on the Group3/Group4 continuum (as determined by DNA-methylation profile). A: Kaplan-Meier plot showing significant differences in MB_{Grp3}/MB_{Grp4} overall survival (patients of all ages) by G3/G4 continuum position divided for convenience as HighG4, LowG4, G3.5, LowG3, HighG3 (these categories are arbitrary divisions of the continuum for the purposes of visualization and comparison and do not represent “real” subgroups). **B:** Forest plot showing univariate Cox models (patients > 3 years) of overall survival containing the variables G3/G4 score (as predicted by DNA methylation) treated as a categorical variable and **C:** MB_{Grp3}/MB_{Grp4} methylation subtype. Related to Figure 2.

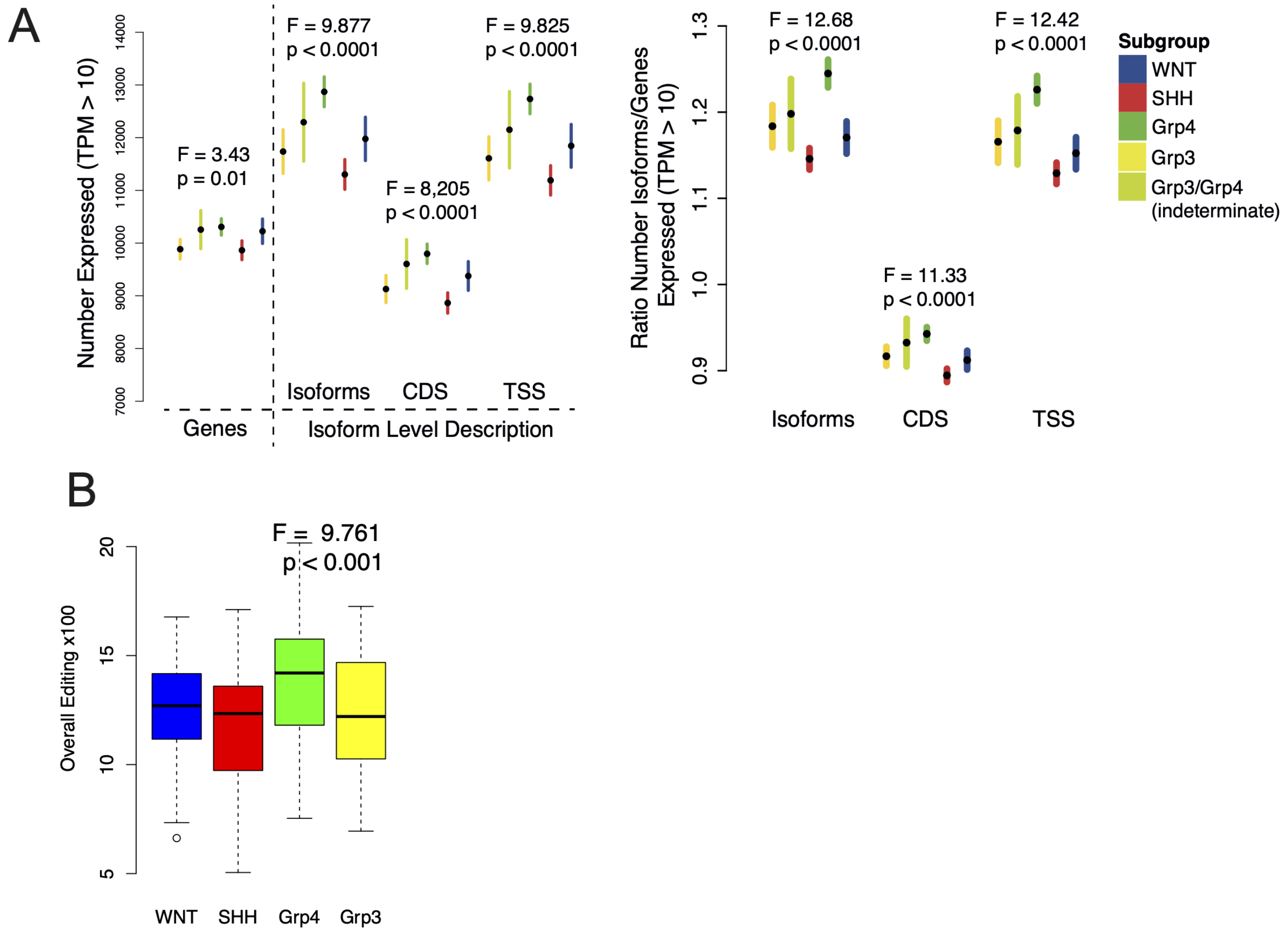

Figure 6S Isoform diversity and level of RNA-editing is related to medulloblastoma subgroup. A: Boxplot showing (left) the distribution by MB subgroup of moderately expressed genes, isoforms, CDS or TSS as defined by a TPM>10 and (right) the same given as a ratio of expressed isoforms, CDS or TSS per expressed genes. **B:** Boxplot showing significant differences in OEI (Overall Editing Index), i.e. level of RNA-editing by MB subgroup. Related to Figure 4.

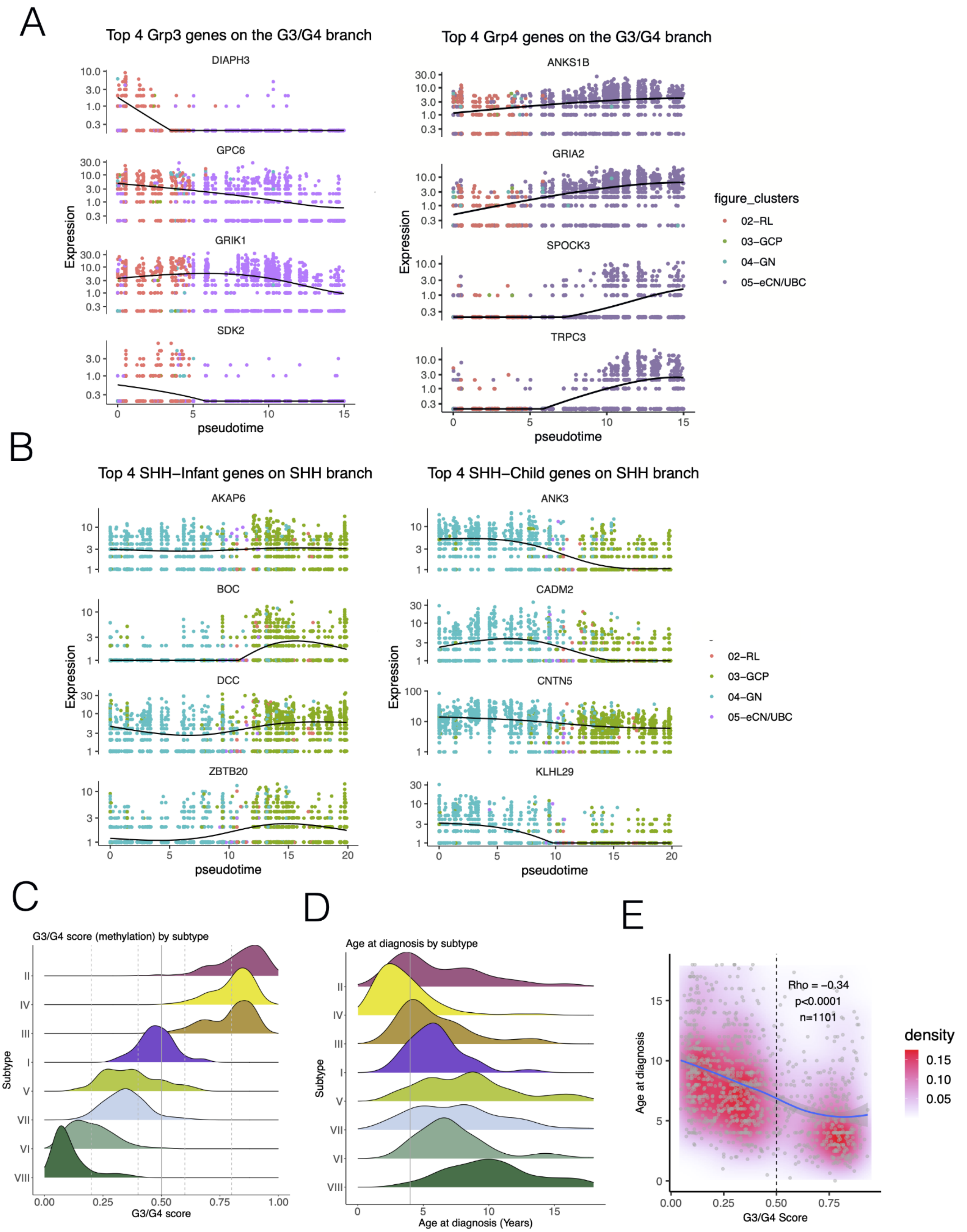

Figure 7S: Expression of Group3/Group4 genes is significantly related to (earlier/later) pseudotime position along the Rhombic Lip – Unipolar Brush cell trajectory and position on the continuum is related to age of onset. Plots showing the per-cell expression of genes whose expression varies according to pseudotime on the **A:** RL to eCN/UBC branch (MB_{Grp3} specific genes are shown on the left and MB_{Grp4} specific genes shown on the right) and the **B:** GCP to GN branch ($MB_{SHH\text{-}Infant}$ specific genes are show on the left and $MB_{SHH\text{-}Child}$ specific genes are shown on the right). Cell type is denoted by color. Black line represents a loess curve. Expression is represented as normalized count data. **C:** Ridgeplots showing distribution of G3/G4 score MB_{Grp3}/MB_{Grp4} patients by methylation subtype (I-VIII) and **D:** distribution of age at diagnosis by DNA methylation subtype (I-

VIII). **E:** Scatterplot showing age at diagnosis by G3/G4 score (as determined by DNA methylation), 2d empirical density is shown as red shading and a loess curve with 95% CI is shown as blue line with grey shading. Related to Figure 6 & Figure 7.
