# Supplementary material for: Medulloblastoma group 3 and 4 tumors comprise a clinically and biologically significant expression continuum reflecting human cerebellar development
Source: Cell Rep. 2022 Aug 3;40(5):111162. doi: 10.1016/j.celrep.2022.111162 (PMC9638015; doi:10.1016/j.celrep.2022.111162)
Supplement: Table S1. Demographic summary of patients used in the study, related to Figure 1 — DOD, dead of disease, LCA, large cell anaplasia; DN, desmoplastic/nodular. [file mmc2.pdf]

Demographic Summary of Patients

	Subgroup						
	Group3	Group3/Group4	Group4	SHH	WNT	MB-NOS	Total
**Sex**							
Male	41/55 (74.5%)	8/12 (66.7%)	96/132 (72.7%)	31/61 (50.8%)	13/28 (46.4%)	5/7 (71.4%)	194/295 (65.8%)
**Age**							
Adult (>16 years)	0/56 (0.0%)	0/12 (0.0%)	2/134 (1.5%)	5/61 (8.2%)	1/29 (3.4%)	1/7 (14.3%)	9/299 (3.0%)
Infant (<3 years)	18/56 (32.1%)	0/12 (0.0%)	6/134 (4.5%)	18/61 (29.5%)	0/29 (0.0%)	2/7 (28.6%)	44/299 (14.7%)
**Clinicopathology**							
Relapse	25/50 (50.0%)	3/10 (30.0%)	43/124 (34.7%)	23/55 (41.8%)	3/28 (10.7%)	4/7 (57.1%)	101/274 (36.9%)
DOD	26/54 (48.1%)	3/12 (25.0%)	39/131 (29.8%)	22/60 (36.7%)	4/29 (13.8%)	4/7 (57.1%)	98/293 (33.4%)
Sub Total Resection	8/50 (16.0%)	2/12 (16.7%)	31/114 (27.2%)	4/55 (7.3%)	1/28 (3.6%)	0/6 (0.0%)	46/265 (17.4%)
LCA	7/42 (16.7%)	2/11 (18.2%)	8/105 (7.6%)	9/50 (18.0%)	4/24 (16.7%)	0/6 (0.0%)	30/238 (12.6%)
DN	1/42 (2.4%)	0/11 (0.0%)	6/105 (5.7%)	16/50 (32.0%)	0/24 (0.0%)	0/6 (0.0%)	23/238 (9.7%)
M+	21/50 (42.0%)	4/12 (33.3%)	33/122 (27.0%)	12/58 (20.7%)	2/29 (6.9%)	1/6 (16.7%)	73/277 (26.4%)
**Mutations**							
GFI1B rearrangement	0/63 (0.0%)	0/13 (0.0%)	3/147 (2.0%)	0/67 (0.0%)	0/31 (0.0%)	0/10 (0.0%)	3/331 (0.9%)
GFI1 rearrangement	5/63 (7.9%)	0/13 (0.0%)	4/147 (2.7%)	0/67 (0.0%)	0/31 (0.0%)	0/10 (0.0%)	9/331 (2.7%)
MYCN amplification	0/47 (0.0%)	1/11 (9.1%)	7/118 (5.9%)	13/55 (23.6%)	0/27 (0.0%)	1/5 (20.0%)	22/263 (8.4%)
MYC amplification	10/47 (21.3%)	1/11 (9.1%)	2/118 (1.7%)	0/54 (0.0%)	0/27 (0.0%)	0/5 (0.0%)	13/262 (5.0%)
TERT	0/42 (0.0%)	0/10 (0.0%)	1/108 (0.9%)	16/51 (31.4%)	0/25 (0.0%)	1/6 (16.7%)	18/242 (7.4%)
TP53	1/63 (1.6%)	0/12 (0.0%)	1/143 (0.7%)	12/67 (17.9%)	1/31 (3.2%)	0/8 (0.0%)	15/324 (4.6%)
CTNNB1	0/50 (0.0%)	0/11 (0.0%)	1/109 (0.9%)	0/53 (0.0%)	25/28 (89.3%)	0/6 (0.0%)	26/257 (10.1%)
**MB(Grp3/Grp4) methylation subtype**							
I	2/63 (3.2%)	1/13 (7.7%)	2/147 (1.4%)	0/67 (0.0%)	0/31 (0.0%)	0/10 (0.0%)	5/331 (1.5%)
II	22/63 (34.9%)	0/13 (0.0%)	0/147 (0.0%)	0/67 (0.0%)	0/31 (0.0%)	0/10 (0.0%)	22/331 (6.6%)
III	10/63 (15.9%)	4/13 (30.8%)	0/147 (0.0%)	0/67 (0.0%)	0/31 (0.0%)	1/10 (10.0%)	15/331 (4.5%)
IV	16/63 (25.4%)	1/13 (7.7%)	0/147 (0.0%)	0/67 (0.0%)	0/31 (0.0%)	0/10 (0.0%)	17/331 (5.1%)
V	3/63 (4.8%)	2/13 (15.4%)	11/147 (7.5%)	0/67 (0.0%)	0/31 (0.0%)	1/10 (10.0%)	17/331 (5.1%)
VI	0/63 (0.0%)	1/13 (7.7%)	18/147 (12.2%)	0/67 (0.0%)	0/31 (0.0%)	0/10 (0.0%)	19/331 (5.7%)
VII	1/63 (1.6%)	1/13 (7.7%)	35/147 (23.8%)	0/67 (0.0%)	0/31 (0.0%)	0/10 (0.0%)	37/331 (11.2%)
VIII	0/63 (0.0%)	0/13 (0.0%)	48/147 (32.7%)	0/67 (0.0%)	0/31 (0.0%)	0/10 (0.0%)	48/331 (14.5%)
Unknown/NA	9/63 (14.3%)	3/13 (23.1%)	33/147 (22.4%)	67/67 (100.0%)	31/31 (100.0%)	8/10 (80.0%)	151/331 (45.6%)
**Methylation subgroup**							
Group3	52/63 (82.5%)	6/13 (46.2%)	3/147 (2.0%)	0/67 (0.0%)	0/31 (0.0%)	1/10 (10.0%)	62/331 (18.7%)
Group4	6/63 (9.5%)	5/13 (38.5%)	125/147 (85.0%)	0/67 (0.0%)	0/31 (0.0%)	1/10 (10.0%)	137/331 (41.4%)
SHH	0/63 (0.0%)	0/13 (0.0%)	0/147 (0.0%)	0/67 (0.0%)	29/31 (93.5%)	0/10 (0.0%)	29/331 (8.8%)
WNT	0/63 (0.0%)	0/13 (0.0%)	0/147 (0.0%)	61/67 (91.0%)	0/31 (0.0%)	6/10 (60.0%)	67/331 (20.2%)
Unknown/NA	5/63 (7.9%)	2/13 (15.4%)	19/147 (12.9%)	6/67 (9.0%)	2/31 (6.5%)	2/10 (20.0%)	36/331 (10.9%)
