# Supplementary material for: Medulloblastoma group 3 and 4 tumors comprise a clinically and biologically significant expression continuum reflecting human cerebellar development
Source: Cell Rep. 2022 Aug 3;40(5):111162. doi: 10.1016/j.celrep.2022.111162 (PMC9638015; doi:10.1016/j.celrep.2022.111162)
Supplement: Document S2. Article plus supplemental information [file mmc6.pdf]

Cell Reports

Article

Medulloblastoma group 3 and 4 tumors comprise a clinically and biologically significant expression continuum reflecting human cerebellar development

Graphical abstract

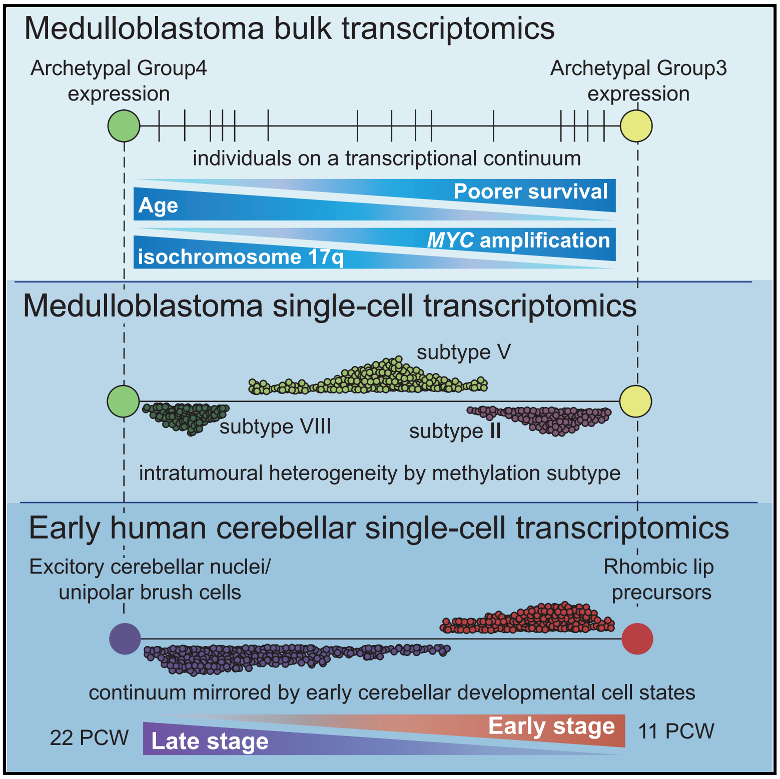

Authors

Daniel Williamson, Edward C. Schwalbe, Debbie Hicks, ..., Abhijit Joshi, Simon Bailey, Steven C. Clifford

Correspondence

daniel.williamson@newcastle.ac.uk (D.W.),
steve.clifford@newcastle.ac.uk (S.C.C.)

In brief

The childhood brain tumor medulloblastoma is classified into multiple DNA methylation-based subtypes. Using RNA-seq, Williamson et al. show that group 3 and group 4 tumors manifest as intermediates on a transcriptomic continuum. Position on the continuum is associated with molecular pathology and disease course. The continuum mirrors early cerebellar development, implicating cells of origin.

Highlights

- Group 3 and group 4 medulloblastoma exist along a transcriptomic continuum
- Position on the continuum is prognostic and reflects key molecular aberrations
- Intratumoral transcriptional heterogeneity is limited according to subtype
- This continuum maps to early fetal development, implicating cells of origin

Williamson et al., 2022, Cell Reports *40*, 111162
August 2, 2022 © 2022 The Author(s).
https://doi.org/10.1016/j.celrep.2022.111162

CellPress

Cell Reports

CellPress
OPEN ACCESS

Article

Medulloblastoma group 3 and 4 tumors comprise a clinically and biologically significant expression continuum reflecting human cerebellar development

Daniel Williamson,[1,9,*] Edward C. Schwalbe,[1,2] Debbie Hicks,[1] Kimberly A. Aldinger,[3] Janet C. Lindsey,[1] Stephen Crosier,[1] Stacey Richardson,[1] Jack Goddard,[1] Rebecca M. Hill,[1] Jemma Castle,[1] Yura Grabovska,[1,4] James Hacking,[1] Barry Pizer,[5] Stephen B. Wharton,[6] Thomas S. Jacques,[7] Abhijit Joshi,[8] Simon Bailey,[1] and Steven C. Clifford[1,*]

[1]Wolfson Childhood Cancer Research Centre, Translational and Clinical Research Institute, Newcastle University Centre for Cancer, Newcastle University, Newcastle upon Tyne, UK

[2]Department of Applied Sciences, Northumbria University, Newcastle upon Tyne, UK

[3]Center for Integrative Brain Research, Seattle Children's Research Institute, Seattle, WA, USA

[4]Division of Molecular Pathology, Institute of Cancer Research, London, UK

[5]Institute of Translational Research, University of Liverpool, Liverpool, UK

[6]Sheffield Institute for Translational Neuroscience, University of Sheffield, Sheffield, UK

[7]Developmental Biology and Cancer Programme, UCL GOS Institute of Child Health, London, and Department of Histopathology, Great Ormond Street Hospital for Children NHS Foundation Trust, London, UK

[8]Department of Neuropathology, Royal Victoria Infirmary (RVI), Newcastle University Teaching Hospitals NHS Foundation Trust, Newcastle upon Tyne, UK

[9]Lead contact

*Correspondence: daniel.williamson@newcastle.ac.uk (D.W.), steve.clifford@newcastle.ac.uk (S.C.C.)

https://doi.org/10.1016/j.celrep.2022.111162

SUMMARY

Medulloblastoma is currently subclassified into distinct DNA methylation subgroups/subtypes with particular clinico-molecular features. Using RNA sequencing (RNA-seq) in large, well-annotated cohorts of medulloblastoma, we show that transcriptionally group 3 and group 4 medulloblastomas exist as intermediates on a bipolar continuum between archetypal group 3 and group 4 entities. Continuum position is prognostic, reflecting a propensity for specific DNA copy-number changes, and specific switches in isoform/enhancer usage and RNA editing. Examining single-cell RNA-seq (scRNA-seq) profiles, we show that intratumoral transcriptional heterogeneity along the continuum is limited in a subtype-dependent manner. By integrating with a human scRNA-seq reference atlas, we show that this continuum is mirrored by an equivalent continuum of transcriptional cell types in early fetal cerebellar development. We identify distinct developmental niches for all four major subgroups and link each to a common developmental antecedent. Our findings show a transcriptional continuum arising from oncogenic disruption of highly specific fetal cerebellar cell types, linked to almost every aspect of group 3/group 4 molecular biology and clinico-pathology.

INTRODUCTION

The division of medulloblastoma (MB) into molecular subgroups has defined the past decade of MB research, making it all but impossible to interpret future findings except through the prism of these fundamental biological subdivisions. MB was first divided into subgroups on the basis of profiling by expression array (Cho et al., 2011; Fattet et al., 2009; Kool et al., 2008; Northcott et al., 2011; Thompson et al., 2006) and, subsequently, DNA methylation array (Hovestadt et al., 2014; Schwalbe et al., 2013). The current consensus is that there exist four major MB subgroups (MB_{SHH}, MB_{WNT}, MB_{Grp3}, MB_{Grp4}), each with unique clinico-biological characteristics (Taylor et al., 2012); MB_{WNT} and MB_{SHH} are named after characteristic disruptions in the WNT (*CTNNB1* mutation (Clifford et al., 2006; Ellison et al., 2005)) and SHH (*PTCH*, *SUFU*, *SMO* mutation, or *GLI2* amplification (Kool et al., 2014)) pathways, respectively. MB_{WNT} denotes an almost entirely curable disease (Ellison et al., 2005), and MB_{SHH} occur more frequently in infants (Kool et al., 2014). The remaining two subgroups, group 3 (MB_{Grp3}) and group 4 (MB_{Grp4}), do not exhibit subgroup-defining mutations (Northcott et al., 2017) but nonetheless possess distinct clinico-biological characteristics; MB_{Grp3} patients have a greater incidence of "high-risk" features such as LCA (large-cell/anaplastic) histology and *MYC* amplification (Kool et al., 2012; Northcott et al., 2012; Ryan et al., 2012; Taylor et al., 2012). MB_{Grp4} tumors more frequently demonstrate isochromosome 17q (i17q) (Sharma et al., 2019). Some overlap in mutational spectrum, DNA methylation, and expression characteristics between MB_{Grp3} and MB_{Grp4} has often been noted, and these are considered more closely related molecularly to one another than to MB_{SHH} and MB_{WNT}, leading them to be considered as a

This is an open access article under the CC BY license (http://creativecommons.org/licenses/by/4.0/).

non-WNT/non-SHH group in the latest World Health Organization (WHO) classification (Louis et al., 2021). The advent of routine MB molecular subgrouping has enabled the current generation of molecularly driven trials (e.g., NCT02066220, NCT01878617, NCT02724579, NCT01125800) (Li et al., 2019; Robinson et al., 2015), which exploit MB_{WNT}/MB_{SHH} biology to stratify treatments or direct biological therapeutics.

Further elaborations of the consensus subgroups were published, based primarily upon methylomic definitions (Cavalli et al., 2017; Northcott et al., 2017; Schwalbe et al., 2017). These were followed by a second consensus study that defined 8 subtypes within MB_{Grp3}/MB_{Grp4}, I–VIII, a number of which comprised a mix of MB_{Grp3} and MB_{Grp4} tumors (Sharma et al., 2019). Furthermore, MB_{SHH} can be further divided into subtypes broadly associated with age at diagnosis (Kool et al., 2014; Schwalbe et al., 2017). The fact that certain MB_{Grp3}/MB_{Grp4} subtypes (e.g., I, V) overlap between MB_{Grp3} and MB_{Grp4} further supports a relationship between the two subtypes. A recent study of MB used single-cell RNA sequencing (scRNA-seq) analysis (Hovestadt et al., 2019) of 4,873 individual cells from 17 MB_{Grp3}/MB_{Grp4} patients to define two transcriptional meta-programs representing a continuum of neuronal cellular differentiation states. This was mirrored in the transcriptional differences between bulk MB_{Grp3} and MB_{Grp4} patients and concluded that $MB_{Grp3/Grp4}$ contain cells along a common continuum of neuronal differentiation, providing further rationale to support this relationship.

Based on murine modeling, expression, and imaging studies (Gibson et al., 2010), MB_{WNT} and MB_{SHH} are believed to derive from two spatially distinct developmental origins in the early hindbrain, lower rhombic lip (RL)/dorsal brainstem, and upper RL/early cerebellum, respectively. The developmental origins of MB_{Grp3} and MB_{Grp4} were investigated in a study mapping subgroup-specific super-enhancer elements, suggesting deep cerebellar nuclei residing in the nuclear transitory zone as the cell of origin for MB_{Grp4} (Lin et al., 2016). More recently, two studies that compared bulk and single-cell transcriptomic (scRNA-seq) MB profiles with developing murine cerebellar scRNA-seq reference datasets described MB_{Grp3} and MB_{Grp4} as most closely resembling Nestin^{+} stem cells (Vladoiu et al., 2019) and unipolar brush cells (UBCs), respectively, highlighting putative cells of origin (Hovestadt et al., 2019; Vladoiu et al., 2019). It is notable that the conclusions of each of these studies rely principally upon cross-species comparisons with murine as opposed to human developmental references. Human RL development is more complex and prolonged than that of the mouse, possessing unique features not shared with any other vertebrates (Haldipur et al., 2019).

Here, we characterize the transcriptomic landscape of 331 primary MB, with clinico-pathological annotation, DNA methylation, and copy-number profiles, and we catalog subgroup-specific isoforms and RNA-editing events. We show that, despite the discrete methylomic subdivisions of the MB_{Grp3}/MB_{Grp4} methylation subtypes I–VIII, these tumors manifest transcriptionally on a bipolar continuum between MB_{Grp3} and MB_{Grp4} archetypes. Moreover, the position of an individual tumor on this continuum is predictive of methylation subtype, prognosis, specific copy-number and mutational alterations, and activation of key molecular pathways and regulatory events. By using human scRNA-seq fetal cerebellar reference data, we show that this continuum mirrors and recapitulates the major developmental trajectories within early human cerebellar development, allowing us to map the interplay between key oncogenic events and putative cells of origin for each MB subtype.

RESULTS

MB shows a continuum of expression between MB_{Grp3} and MB_{Grp4}

RNA-seq (~90 million paired-end reads) was performed on 331 snap-frozen primary samples from patients with a diagnosis of MB (Table S1). Transformed gene-level read counts were subject to consensus non-negative matrix factorization (NMF) clustering with resampling to determine the most stable number of clusters and metagenes (i.e., major biological effects described by multiple genes and summarized as a single score). As expected, a four-metagene/four-cluster solution was optimally stable, reflecting the four major consensus subgroups as currently understood (Figure 1A). Approximately 3% (10/331) of samples were defined as non-classifiable (i.e., low probability of classification). Approximately 4% (13/331) samples could only be classified as indeterminate MB_{Grp3}/MB_{Grp4} (i.e., confidently classifiable as either MB_{Grp3} or MB_{Grp4} but not specific as to which). The distribution of clinico-biological features was consistent with previously described features of the consensus MB subgroups (Figures 1A and S1A); for instance, chromosome 6 loss in 83% (24/29) of MB_{WNT}.

The two metagenes that described MB_{Grp3} and MB_{Grp4} samples were notably gradated and overlapping in an anticorrelative manner (Figure 1A), implying that, contrary to some previous descriptions using expression microarrays (Cavalli et al., 2017), MB_{Grp3} and MB_{Grp4} are not distinct transcriptional entities but rather exist as a continuum between two transcriptional polarities that we refer to here as G3 and G4. To describe this continuum, we created a continuous score (G3/G4 score) scaled between 0 and 1 to reflect the proportionate amount of G3/G4 metagene expression in each MB_{Grp3}/MB_{Grp4} (i.e., all non-WNT/non-SHH tumors) whereby a score of "0" indicates a 100% G4 tumor and "1" indicates a 100% G3 (Figure 1A). This was applied to the 223 samples classified as MB_{Grp3}, MB_{Grp4}, or intermediate MB_{Grp3}/MB_{Grp4}.

We regard these results as showing that no individuals fall into discrete transcriptional subtypes with respect to the G3/G4 continuum, but for convenient comparison, we subdivided the expression continuum (G3/G4 score) into five purely notional quantiles: highG4 (0–0.2, n = 69/223 [31%]), lowG4 (0.2–0.4, n = 60/223 [27%]), G3.5 (0.4–0.6, n = 39/223 [17%]), lowG3 (0.6–0.8, n = 22/223 [10%]), and highG3 (0.8–1 G3/G4 score, n = 33/223 [15%]). All of the samples with >0.5 G3/G4 score were classified as MB_{Grp3}. Notably, 15/20 (75%) MB_{Grp3}/MB_{Grp4} samples, which showed disagreement in classification between RNA-seq and DNA methylation array, were classified as indeterminate MB_{Grp3}/MB_{Grp4} by RNA-seq (Figure 1A). Examining the MB_{Grp3}/MB_{Grp4} subtype (I–VIII) calls by t-distributed stochastic neighbor embedding (t-SNE) (Figure 1B) shows clustering by subtype, suggesting that each methylation subtype imparts distinct secondary expression characteristics beyond the primary G3/G4 continuum metagene. Regardless, the MB_{Grp3}/MB_{Grp4}

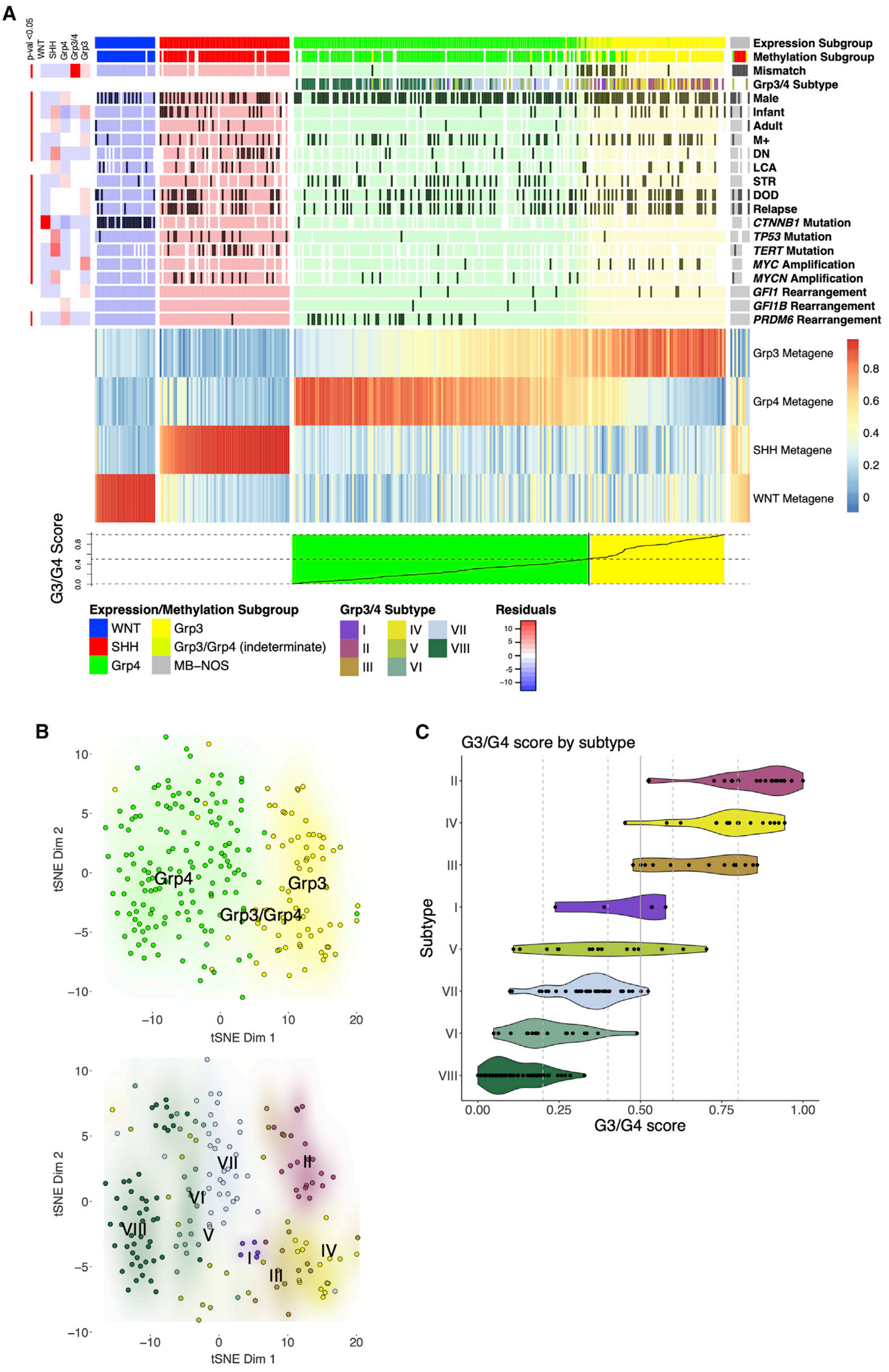

(legend on next page)

subtypes may be broadly ordered upon the G3/G4 continuum in partially overlapping domains from most group 4-like to most group 3-like (VIII, VI, VII, V, I, III, IV, II, respectively) (Figure 1C).

Specific clinico-biological features were significantly non-randomly distributed across the G3/G4 continuum (Figure 2A). For instance, the distribution of patients with LCA pathology along the continuum is significantly different from those without LCA pathology (D = 0.339, p = 0.046, n = 158); there appears to be more LCA patients toward the G3 end of the continuum. The distribution along the continuum of patients with certain large (arm level/chromosomal) copy-number alterations are significantly differently distributed compared to those without. Most notably, patients with i17q (D = 0.402, p < 0.001, n = 201) and chromosome 8 gain (D = 0.69, p < 0.001, n = 201) are more frequent toward the G4 and G3 poles, respectively (Figure S1). Mutations are not frequent in MB_{Grp3}/MB_{Grp4} (Northcott et al., 2017); however, non-synonymous mutations of *ZMYM3* and *KDM6A* are significantly non-randomly distributed with respect to the continuum (each p < 0.01) (Figure S2).

We examined the relationship between the G3/G4 score and prognosis. Again, we divided the G3/G4 score into notional quantiles for the purposes of visualization/description showing a progressively poorer 5-year overall survival (OS) across the continuum: Log rank (test for trend) $Z = -2.97$, p = 0.003, n = 191, highG3 = 46%, lowG3 = 57%, G3.5 = 71%, lowG4 = 81%, and highG4 = 76% (Figure 2B). Most important, Cox regression indicates that a continuous G3/G4 score is highly significant (relative risk [RR] 4.7, p = 0.003, n = 191) showing an increase in RR of death of 4.7 times greater for a patient with a G3/G4 score of 0 compared to a score of 1.

To assess any independent prognostic significance, we used multivariable Cox regression analysis of progression-free survival, including highG3 status alongside other risk factors (*MYC* amplification, LCA histology, and metastatic disease). The analysis showed that the highG3 status—chosen over a continuous variable in this instance as it overlaps most with other risk factors—retains significance (RR = 2.4, p = 0.014, n = 135), indicating that the G3/G4 score possesses significant independent prognostic power that is distinct from its association with other “high-risk” disease features (Figure 2C).

A G3/G4 continuum score can be reverse-engineered from DNA methylation profiles to validate clinico-pathological associations

A series of sample cohorts of MB_{Grp3}/MB_{Grp4} with DNA methylation profiles have previously been published by our group and others (Cavalli et al., 2017; Northcott et al., 2017; Schwalbe et al., 2017; Sharma et al., 2019). To these we added 166 profiles to produce a large cohort (n = 1,670) better powered to validate and further expand the findings we made using transcriptomic datasets. We therefore explored the possibility of reverse-engineering a G3/G4 score from DNA methylation data. Using the same method as used for expression was impossible, given that the constrained range (i.e., 0-1 [fully unmethylated]–[fully methylated]) and bimodal distribution of CpG methylation does not lend itself straightforwardly to a continuous score (Figure S3A). Unlike expression, which tends to follow a log-linear association with G3/G4 score, methylation follows a sigmoidal distribution from hypo- to hypermethylation or vice versa. The inflection point along the G3/G4 continuum at which these CpGs “switch” from one state to the other varies by CpG (Figures S3B–S3D). We trained a classifier using a training cohort of MB_{Grp3}/MB_{Grp4} samples for which we possessed both RNA-seq and DNA methylation profiles (n = 192). Pre-selecting 400 cross-validated CpG features that distinguish between each of the G3/G4 categorical states, we used these to train a random forest classifier to accurately predict (root-mean-square error [RMSE] = 0.036) a G3/G4 score from DNA methylation data alone (Figure S3E).

Using this larger MB_{Grp3}/MB_{Grp4} methylation cohort, we were able to demonstrate significant differences in distribution along the continuum for patients with infant status (<3 years), metastases, LCA, and *MYC* amplification (each progressively more frequent toward the G3 pole), and mutations of *PRDM6*, *KDM6A*, *KMT2C*, and *ZMYM3* (progressively more frequent toward the G4 pole) compared to patients who lack those features (each p < 0.001; Figure S4A). Likewise, chromosomal gains of 1q, 5, 6, 8, and 16q (each p < 0.001) were progressively more frequent toward the G3 pole, and i17q (p < 0.001) was progressively more frequent toward the G4 pole (Figure S4A). These findings thus validated our findings from the initial RNA-seq cohort.

The larger cohort size allowed us to also explore the relationship between the G3/G4 continuum and the MB_{Grp3}/MB_{Grp4} subtypes (I–VIII) as well as their previously reported clinico-pathological/mutational characteristics (Sharma et al., 2019). The MB_{Grp3}/MB_{Grp4} subtypes as predicted from DNA methylation data once again occupy discrete but partly overlapping domains within the G3/G4 continuum, broadly ordered, as per the RNA-seq-only cohort, from most archetypally MB_{Grp4} to MB_{Grp3} - VIII, VI, VII, V, I, III, IV, II, respectively (Figure 2D).

We next asked whether the variation in the distribution of clinicopathological features and mutation previously described

Figure 1. Group 3/group 4 medulloblastoma (MB) form a transcriptional continuum

(A) Heatmap showing 4 consensus NMF metagenes calculated for n = 331 MB and grouped by subgroup. MB_{Grp3}/MB_{Grp4} individuals are ordered by G3/G4 score. Annotation shows subgroup as determined by RNA-seq (expression subgroup), subgroup as determined by methylation (methylation subgroup), and methylation MB_{Grp3}/MB_{Grp4} subtype (I–VIII) as per Sharma et al. (2019) defined using Molecular Neuropathology version 2.0 (MNPv2) classifier (Capper et al., 2018) (Grp3/4 subtype). All of the other characteristics are indicated to be present or not by dark gray shading according to the following scheme: Infant, age at diagnosis younger than 3 years; Adult, age at diagnosis older than 16 years; DN, desmoplastic/nodular; LCA, large-cell/anaplastic; STR, subtotal resection; DOD, dead of disease. Side annotation (top left) shows a heatmap of chi-square residuals indicating subgroup enrichment and significance where relevant. The line plot (bottom) shows the G3/G4 score.

(B) t-SNE plot showing MB_{Grp3}/MB_{Grp4} samples shaded by subgroup (top) and methylation MB_{Grp3}/MB_{Grp4} subtype (I–VIII) (bottom). Points where subtype (I–VIII) could not be determined confidently are not shown.

(C) Violin plot showing G3/G4 score by MB_{Grp3}/MB_{Grp4} subtype (I–VIII).

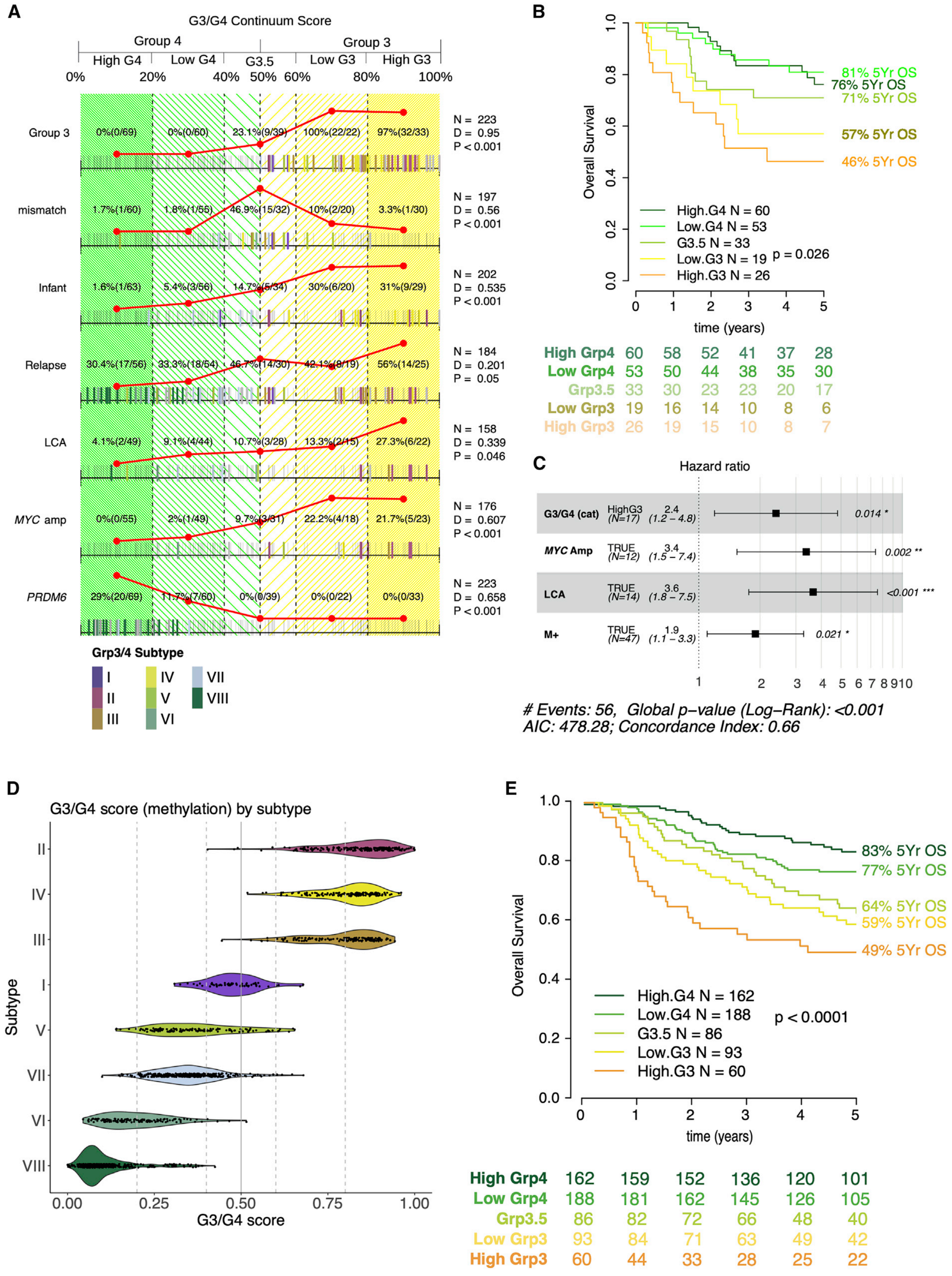

(legend on next page)

as being characteristic of MB_{Grp3}/MB_{Grp4} subtypes (I-VIII) (Sharma et al., 2019) were attributable to their position on the G3/G4 continuum, the MB_{Grp3}/MB_{Grp4} subtype (I–VIII), or, indeed, both. Certain frequent clinicopathological features and copy-number changes (e.g., metastatic disease, *MYC* amplification, LCA histology, i17q, loss of chromosome 8, gain of chromosome 5) are significantly non-randomly distributed with respect to G3/G4 continuum, even within individual subtypes (Figures S4B and S4C). For example, 100% (11/11) of subtype III with *MYC* amplifications are highG3 compared to 59% (69/117) without *MYC* amplification. The presence of i17q as the only major chromosomal alteration is a highly characteristic change in subtype VIII, but when considering only MB subtype, VIII is still significantly enriched at the highG4 end of the continuum (D = 0.162, p = 0.014).

The relationship between G3/G4 score and risk of death is significant and striking, allowing us to validate the findings of our RNA-seq cohort with greater confidence. Again, for the purposes of visualization/description, we divided patient G3/G4 scores into notional quantiles: Patients older than 3 years log rank (test for trend) $Z = -4.89$, $p < 0.0001$, n = 589, highG3 = 49%, lowG3 = 59%, G3.5 = 64%, lowG4 = 77%, and highG4 = 83% (Figure 2E). A similar result is found in patients of all ages: Log rank (test for trend) $Z = -5.49$, $p < 0.0001$, n = 654 (Figures S5A and S5B). Most important, G3/G4 score is efficiently modeled as a continuous variable using Cox proportional hazards. Again, patients older than 3 years shows a 3× increased risk of death from one end of the continuum to the other (RR = 3, n = 589, $p < 0.001$). We also note that MB_{Grp3}/MB_{Grp4} subtypes (I–VIII) are significantly associated with OS (n = 524, $p < 0.001$) (Figure S5C).

The G3/G4 continuum is associated with differential regulation of oncogenic/developmental pathways

The expression of 590 genes is significantly correlated with the G3/G4 score in our RNA-seq cohort ($p < 0.01$, $\log_2$ fold change >10, n = 223), increasing/decreasing log linearly across the continuum. Most notably, *MYC* expression correlates significantly with the G3/G4 score (rho = 0.73, $p < 0.001$, n = 223)—approximately 46× greater from the G4 end of the continuum to the G3 (Figure 3A). Performing gene set enrichment analysis (GSEA), we observed that transcriptional targets of MYC were also significantly upregulated (NES = 3.37, p = 0.007) (Figure 3B). Single-sample GSEA (ssGSEA) analysis (Hänzelmann et al., 2013) was used to represent the activation/repression of pathways/signatures for each individual and found several oncogenic pathways that were progressively activated or repressed in a manner significantly correlated (each $p < 0.001$) with the G3/G4 continuum, including MYC, cell cycle, mammalian target of rapamycin (mTOR), transforming growth factor β (TGF-β) (activated at the G3 pole), and NOTCH (activated at the G4 pole) (Figure 3C). In addition, a broad pattern of progressive neuronal differentiation at the G4 pole and photoreceptor (CRX/NRL) characteristics at the G3 pole of the G3/G4 continuum were observed.

We examined differentially methylated regions (DMRs) within previously identified MB_{Grp3}/MB_{Grp4} specific enhancer loci (Lin et al., 2016), identifying 45 that also overlapped with gene promoters; each "switched" from hypomethylated to hypermethylated or vice versa at specific points along the G3/G4 continuum. The expression of 33/45 of these genes is significantly correlated with the G3/G4 continuum ($p < 0.01$). This switching appears progressive, with certain MB_{Grp3}/MB_{Grp4} enhancer loci "switching" earlier and others later. For instance, the enhancer/DMR loci overlapping with the promoters of MB lineage development/differentiation genes *LHX1*, *NEUROD2*, *LMX1A*, and *HLX* on average "switch" at points 0.23, 0.49, 0.56, 0.87, respectively, on the G3/G4 continuum (Figures 3D and 3E). We note also that the expression of each of these genes is significantly correlated with the G3/G4 continuum and DMR methylation (each $p < 0.01$). If we presuppose a model by which the G3/G4 continuum reflects interruption of early developmental cell fate at different points in different patients, then this observed switching is consistent with a developmental identity controlled by cumulative changes in underlying epigenetic architecture (i.e., patterns of methylation and/or enhancer usage) throughout a transition from an MB_{Grp3} to a MB_{Grp4} cell state.

The G3/G4 continuum is associated with post-transcriptional regulation of isoform expression and RNA editing

To explore the clinico-biological significance of differentially expressed transcriptional isoforms across subgroups, Kallisto (Bray et al., 2016) was used to estimate their abundance. Taking transcripts per million (TPM) >10 as indicative of a moderate-to-highly expressed isoform, it is notable that the diversity of isoforms being expressed across subgroups was significantly greater in MB_{Grp4} than MB_{Grp3} ($p < 0.001$, F = 9.877) (Figure S6A).

A total of 153 genes were identified whose expression overall is invariant but for which the expression of specific isoforms

Figure 2. Clinico-pathology, subtype, and survival are related to an individual's position on the group 3/group 4 continuum

(A) Rug plot showing distribution of clinico-pathological features with respect to G3/G4 score. Summary counts are given according to the divisions of highG4, lowG4, G3.5, lowG3, and highG3 (these categories are arbitrary divisions of the continuum for the purposes of visualization and comparison and do not represent "real" subgroups) and reflected by the red line plots. The presence of a feature is indicated by a bold tick mark, the color of which indicates MB_{Grp3}/MB_{Grp4} methylation subtype (I–VIII). Adjusted p values for a Kolmogorov-Smirnoff statistic (D) are shown to denote non-random distribution of features with respect to G3/G4 score. Mismatch, mismatch between methylation and expression call; Infant, age at diagnosis younger than 3 years; M+, metastatic; DOD, dead of disease; LCA, large-cell/anaplastic; PRDM6, *PRDM6* rearrangement.

(B) Kaplan-Meier plot showing significant differences (Log-Rank test for trend) in MB_{Grp3}/MB_{Grp4} overall survival by G3/G4 continuum position.

(C) Forest plot showing a multivariate Cox model fitted to progression-free survival and containing the independently significant variables highG3, *MYC* amplification, LCA, and M+.

(D) Violin plot showing G3/G4 score (derived from methylation) by MB_{Grp3}/MB_{Grp4} (I–VIII) subtype.

(E) Kaplan-Meier plot showing significant differences (Log-Rank test for trend) in MB_{Grp3}/MB_{Grp4} overall survival in patients aged older than 3 years by G3/G4 score (as derived from methylation values); n = 589.

CellPress
OPEN ACCESS

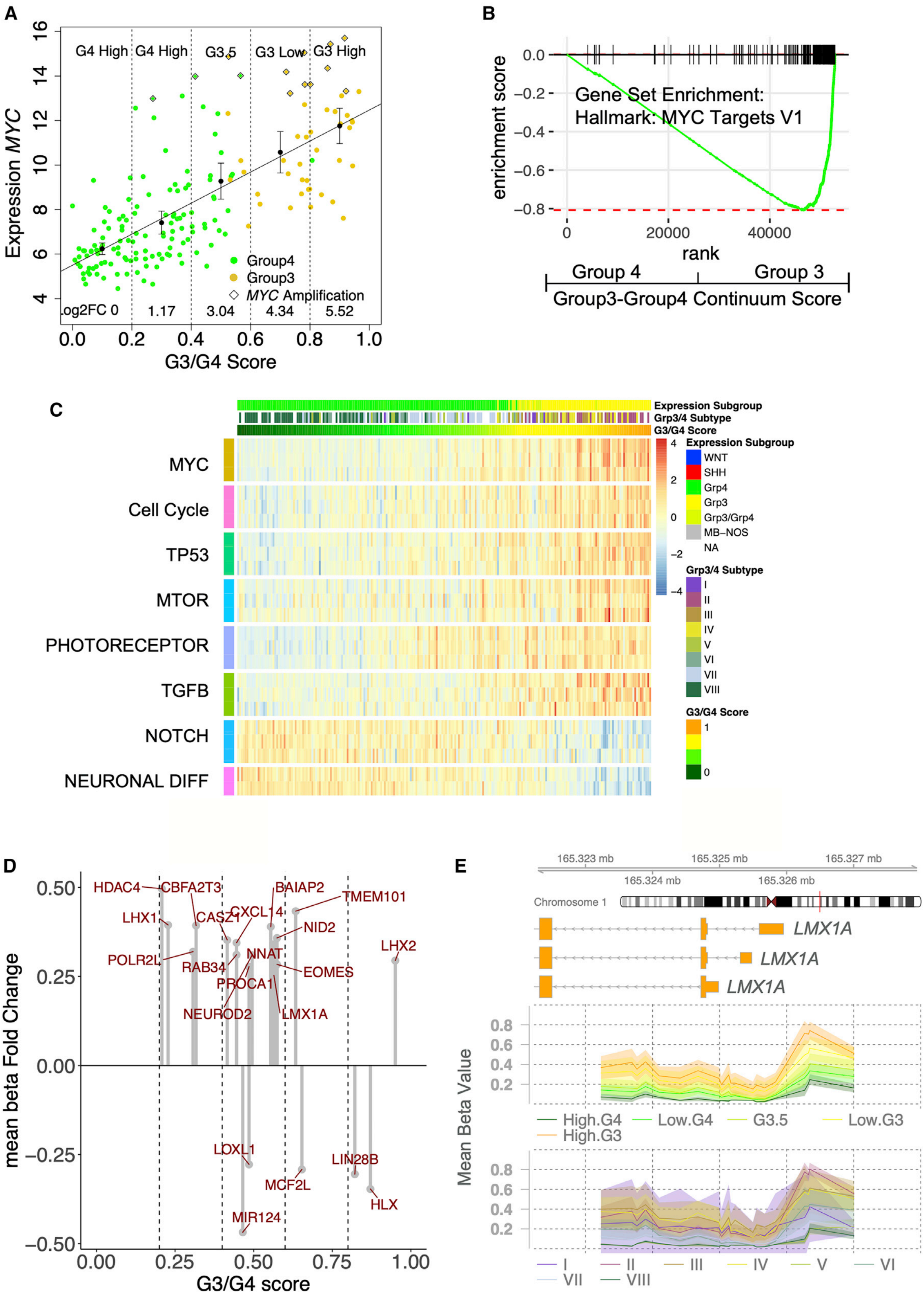

(legend on next page)

correlates significantly with G3/G4 score (Figure 4A). For instance, the overall expression of *general transcription factor IIi (GTF2I)* is ubiquitous, but a progressive isoform switch corresponding to the balance between β/δ (*GTF2I-215/GTF2I-218*) and α/γ (*GTF2I-221/GTF2I-212*) isoforms correlates significantly to G3/G4 score (Figure 4B). These isoform switches are known to alter protein stability (Shirai et al., 2015) and subcellular localization (Shirai et al., 2017).

A total of 4,668,508 established RNA editing sites were profiled using the QEdit/Reditools pipeline (Lo Giudice et al., 2020). We observed significant differences in overall A-I editing level. The Overall Editing Index (OEI, i.e., the total number of reads with G at all known editing positions over the number of all reads covering the positions) differs significantly with respect to subgroup ($F = 9.761$, $n = 223$, $p < 0.001$). Post hoc testing showed RNA editing events in MB_{Grp4} to be significantly more numerous than in MB_{Grp3} and MB_{SHH} (each $p < 0.01$) (Figure S6B). Analysis of 5,174 non-synonymous RNA editing sites showed 32 significantly differentially edited with respect to the G3/G4 continuum ($p < 0.05$; Figure 4C), the majority of which were more highly edited in MB_{Grp3}. One such RNA editing site is *AZIN1* chr8:103841636T>C, known to result in a S367G substitution that causes conformational changes, cytoplasmic-to-nuclear translocation, and gain of function, increasing tumor potential in hepatocellular carcinoma (Chen et al., 2013), non-small cell lung cancer (Hu et al., 2017), colorectal cancer (Shigeyasu et al., 2018), and gastric cancer (Okugawa et al., 2018) (Figure 4D). It is also notable that *ADAR1* and *ADAR2* expression are both correlated with G3/G4 score ($rho = 0.54$, $p < 0.001$ and $rho = 0.33$, $p < 0.001$, $n = 223$, respectively), although expression was higher in MB_{Grp3}, which may speak to a context-dependent effect on specific loci.

Intratumoral cellular heterogeneity with respect to the G3/G4 continuum is apparent but constrained by subtype

We projected our MB_{Grp3}/MB_{Grp4} metagenes onto a MB_{Grp3}/MB_{Grp4} scRNA-seq dataset comprising 4,256 cells from 15 individuals (5xSubtype-II, 2xSubtype-III, 1xSubtype-I, 2xSubtype-V, 4xSubtype-VIII) previously published by Hovestadt et al. (2019). The approach used to derive these metagenes is very similar methodologically to the way Hovestadt et al. derived their metaprograms (e.g., use of NMF, projection between bulk and single cell) and both indicate a continuum of scores at both the bulk and single-cell level. We projected our bulk metagenes (describing group 3/group 4 transcriptional variability in 223 bulk tumor profiles) onto scRNA-seq data. In contrast, Hovestadt et al. projected their scRNA-seq metagenes (describing neuronal cellular differentiation and calculated from 17 $MB_{Grp3/Grp4}$) onto bulk expression microarray samples. Our approach allowed us to impose a limit and scale between the extremities of tumor MB_{Grp3} and MB_{Grp4} transcriptional states, and in so doing, place each cell within a given sample on a common scale with our bulk tumors, allowing us to align cells with key tumor features such as subtype.

MB_{Grp3} individuals were described by Hovestadt et al. as being dominated by cells with an undifferentiated progenitor-like expression program and MB_{Grp4} dominated by a differentiated neuronal-like program; to some extent our MB_{Grp3} and MB_{Grp4} metagenes appear to equate with the meta-programs described by Hovestadt et al., and it is quite possible that both are describing similar phenomena. Of the 100 genes selected as the top genes by Hovestadt et al., 7/100 (metaprogram B—undifferentiated; e.g. *LAPTM4B*, *MYC*, *HLX)* and 8/100 (metaprogram C—differentiated; e.g. *KCNA1*, *ABLIM1*, *SPOCK2*) would have been selected in the equivalent top 100 from our analysis. Notably, 31 Hovestadt et al. metaprogram B/C genes (e.g., *ORC4*, *H3F3B*, *GNB2L1*) were invariant with respect to the G3/G4 continuum.

By placing bulk and scRNA-seq on a common scale, we show that the distribution of G3/G4 scores at the single-cell level indicates a certain amount of intratumoral cellular variation (Figure 5A), but that the majority of cells fall within the same G3/G4 range observed in the equivalent subtype bulk RNA-seq profiles (Figure 5B). For example, among MB subtype VIII individuals, 78% (667/853) of cells fall within the G3/G4 score 0–0.25 range, as per the equivalent subtype VIII bulk profiles (Figure 5B). We should note that different bulk $MB_{Grp3/Grp4}$ subtypes and their respective scRNA-seq populations occupy either a broader or narrower space on the G3/G4 continuum depending on the subtype; subtype V, for instance, is comparatively broad. In short, the phenomenon of a G3/G4 continuum observed in bulk RNA-seq analysis is produced by populations of individual cells, which themselves display continuous G3/G4 expression characteristics. These are constrained to occupy a discrete part of the G3/G4 continuum as dictated by their MB_{Grp3}/MB_{Grp4} (I–VIII) subtype.

MB subtypes and the G3/G4 continuum are mirrored in early human cerebellar development

The origins of MB within spatially and temporally distinct regions of the fetal cerebellum (upper RL/granule cell [GC] lineage for MB_{SHH} and lower RL for MB_{WNT}) have been established primarily

Figure 3. Position on the group 3/group 4 continuum corresponds linearly to oncogenic pathway activation and methylation of lineage-specific enhancers

(A) Scatterplot showing significant correlation ($p < 0.001$) between *MYC* expression and G3/G4 score. Log-linear line of best fit is shown. Dotted lines divide into highG4, lowG4, G3.5, lowG3, and highG3 (these categories are arbitrary divisions of the continuum for the purposes of visualization and comparison and do not represent "real" subgroups), and log2 fold changes for each category relative to highG4 are shown. Error bars represent standard error of mean.

(B) GSEA enrichment plot showing significant enrichment of MYC target genes. Genes were ranked by correlation with G3/G4 score.

(C) Heatmap of ssGSEA results showing level of pathway enrichment for 223 MB_{Grp3}/MB_{Grp4} individuals ordered by G3/G4 score. MsigDB pathways are curated into pathways (see STAR Methods).

(D) Lollipop plot showing mean beta fold change for DMRs within MB_{Grp3}/MB_{Grp4} specific enhancers/super-enhancers. The position on the x axis reflects the average point on the continuum at which the methylation level switches from hypo- to hypermethylation.

(E) Plot showing an MB_{Grp3}/MB_{Grp4}-specific enhancer within the MB_{Grp3}-specific gene, *LMX1A*, which overlaps with a differentially methylated region significantly associated with the G3/G4 continuum. The mean beta value per G3/G4 category (highG4, lowG4, G3.5, lowG3, highG3) and MB_{Grp3}/MB_{Grp4} subtype (I–VIII) are shown by line and the 95% confidence interval (CI) by shaded area.

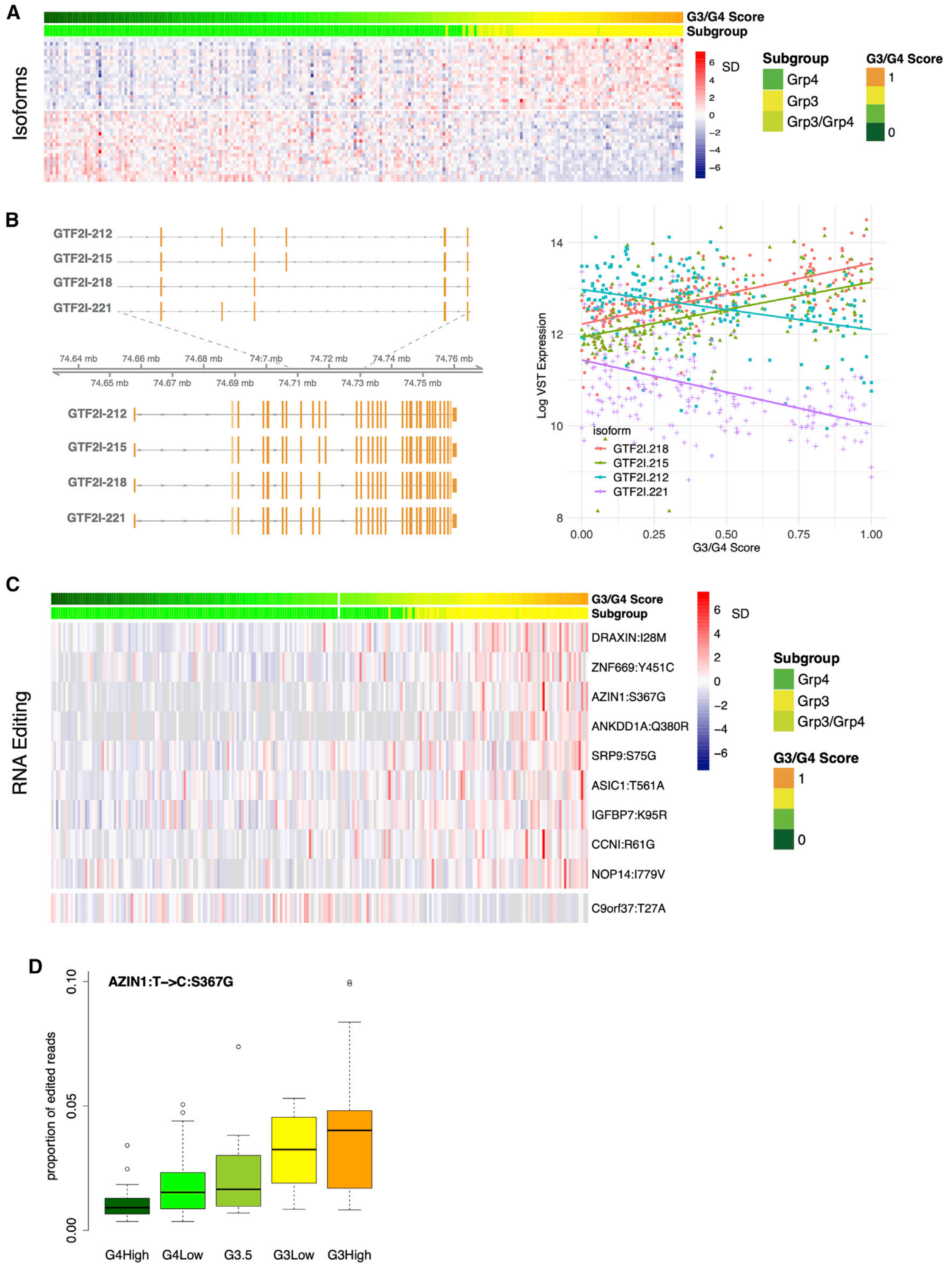

Figure 4. Position on the group 3/group 4 continuum is linearly associated with isoform usage and non-synonymous RNA editing events

(A) Heatmap showing expression of top significantly differentially expressed isoforms of genes whose overall expression is otherwise not significantly differentially expressed with respect to G3/G4 score.

(B) Schematic showing exon structure of 4 *GTF2I* isoforms significantly differentially expressed with respect to G3/G4 score (left) and scatterplot showing expression of these *GTF2I* isoforms versus G3/G4 score; line represents fitted log-linear model NB: *GTF2I* is not significantly differentially expressed at the gene level.

(legend continued on next page)

by mouse modeling (Gibson et al., 2010; Lin et al., 2016) and, more recently, by comparison with reference to mouse fetal cerebellum scRNA-seq datasets, which suggest a UBC origin for MB_{Grp4} (Hovestadt et al., 2019; Vladoiu et al., 2019). Such comparisons in embryonal tumors are predicated on the idea that partial transformation in an early prenatal cell interrupts development/differentiation, resulting in a proportion of the expression characteristics of the tumor-initiating cell being retained.

Here, we avoid any cross-species comparisons by using instead a human fetal cerebellum scRNA-seq reference set (69,174 cerebellar cells 9–21 post-conception weeks [PCWs]). We reconstructed a pseudotemporal cellular trajectory within a broadly defined RL lineage (12,243 cells, comprising RL precursors, excitatory cerebellar nuclei [eCN]/UBC, GC precursors [GCPs], and GC neurons subdivided into four clusters [GN]) (Figure 6A). We projected our four subgroup metagenes onto these cerebellar cells, identifying those cells that showed the highest expression of each metagene. As an alternative analysis, we also performed canonical correlation analysis (CCA) and achieved comparable results (see description in STAR Methods). These cells occupy distinct branches of our lineage. High MB_{WNT} metagene-expressing cells, as expected, occupy a discrete subset of the RL precursors (Figure 6B). High MB_{Grp3}/MB_{Grp4} metagene-expressing cells occupy a distinct eCN/UBC branch beginning with RL precursors (highly expressing MB_{Grp3} metagenes) and transitioning midway to eCN/UBC cells highly expressing the MB_{Grp4} metagene (Figure 6B). This cell trajectory in effect mirrors the G3/G4 continuum. This can be demonstrated formally by calculating a projected per-cell G3/G4 score, revealing a smooth transition from a MB_{Grp3}-like to a MB_{Grp4}-like expression state (Figure 6C). More straightforwardly, this is demonstrated by observing the significant change in expression with respect to pseudotime of those G3/G4 continuum-associated genes whose expression is sufficiently high to be consistently detectable within the relatively low-depth scRNA-seq data (each $p < 0.01$; Figure S7A).

Cells that express the MB_{SHH} metagene most highly, as expected, occupy a GC developmental branch beginning with GCPs and extending partly into the earliest GN cell types (Figure 6B). Two metagenes representing $MB_{SHH\text{-}Infant}$ (primarily patients younger than 4 years) and $MB_{SHH\text{-}Child}$ (primarily patients older than 4 years), as described in previous studies (Kool et al., 2014; Schwalbe et al., 2017), were also projected onto the cells in this branch. This indicated a switch midway through the GC pseudotemporal lineage from a predominantly $MB_{SHH\text{-}Infant}$ metagene to a predominantly $MB_{SHH\text{-}Child}$ metagene expression; this coincided approximately with the first transition from GCPs to GNs (Figure 6D). Again, where the expression of individual genes that distinguish infant MB_{SHH} from childhood MB_{SHH} were sufficiently detectable within the scRNA-seq profiles, they were significantly associated with pseudotime (each $p < 0.01$; Figure S7B).

Thus, by aligning the oncogenic G3/G4 scale with the pseudotemporal scale, we were able to order and align tumorigenic events to specific points within fetal cerebellar developmental lineages (Figure 7). *MYC* amplification, for instance, tends to coincide with the earlier RL pseudotemporal space, as opposed to *KDM6A* mutation, which occupies the later, more differentiated eCN/UBC space. Likewise for aneuploidies, the gain of chromosome 8 coincides with the earlier RL developmental space and i17q (as the sole copy-number alteration) with the later eCN/UBC cell types.

We note that as with the pseudotemporal transition from MB_{Grp3} to MB_{Grp4} or $MB_{SHH\text{-}Infant}$ to $MB_{SHH\text{-}Child}$, there is also a literal temporal transition. The cerebellar cells most closely associated with the archetypal MB_{Grp3} are predominant at 11 PCW (and possibly before). By 18 PCW, those most closely associated with the archetypal MB_{Grp4} predominate. This persists until at least 20 PCW. On the RL to GN branch, the cells most closely associated with $MB_{SHH\text{-}Infant}$ are predominant at PCW 11 and reduced by PCW 20, at which point $MB_{SHH\text{-}Child}$-associated cells predominate (Figure 7B). We should temper this observation by saying that the uniformity of sampling at each of these time points is uncertain.

This temporal staging from early to late forms of MB_{Grp3}/MB_{Grp4} is also mirrored in the average age of onset of disease. The distribution of age at diagnosis of each MB_{Grp3}/MB_{Grp4} (I–VIII) subtype closely parallels the distribution across the G3/G4 continuum (Figure S7C), and there is a significant correlation between G3/G4 score and age at diagnosis (Figure S7D).

DISCUSSION

Here, we show that, in regard to their transcriptomes, the primary intertumoral variation in MB_{Grp3}/MB_{Grp4} patients is continuous, in contrast to the discrete nature of the methylation MB_{Grp3}/MB_{Grp4} subtypes (I–VIII) (Cavalli et al., 2017; Northcott et al., 2017; Schwalbe et al., 2013; Sharma et al., 2019). This is not in itself contradictory, as we show that the MB_{Grp3}/MB_{Grp4} methylation subtypes are ordered along the G3/G4 continuum in discrete but partially overlapping domains (Figure 1D). Furthermore, as has been demonstrated previously (Cavalli et al., 2017; Sharma et al., 2019), the methylation subtypes are reflected to some extent in their expression profiles (Figure 1C). Nonetheless, these are shown here to be secondary expression characteristics subordinate to the overarching primary expression characteristic that is the G3/G4 continuum.

The position of an individual MB_{Grp3}/MB_{Grp4} tumor upon the continuum is significantly different in individuals with and without certain mutations, copy-number aberrations, clinicopathology, and histopathology. This is to be expected, as many of these have been shown to be non-randomly associated with MB_{Grp3}/MB_{Grp4} subtypes (Sharma et al., 2019). That both methylation subtype and the expression continuum are related to key tumor characteristics and, indeed, to one another is

(C) Top 10 significantly differentially edited non-synonymous RNA editing positions with respect to G3/G4 score.

(D) Boxplot showing level of T > C RNA editing at a non-synonymous position S367G within *AZIN1* divided into highG4, lowG4, G3.5, lowG3, and highG3 (these categories are arbitrary divisions of the continuum for the purposes of visualization and comparison and do not represent "real" subgroups); level of editing is significantly associated with G3/G4 score.

CellPress
OPEN ACCESS

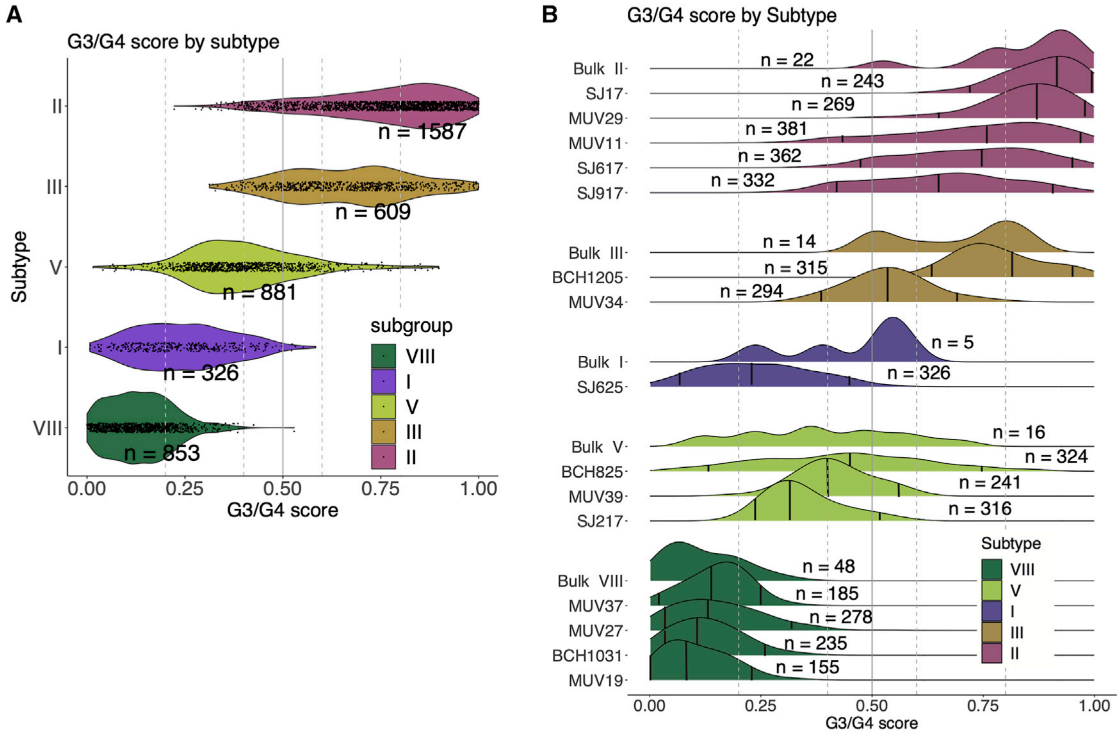

Figure 5. Distribution of single cells along the group 3/group 4 continuum is limited according to DNA methylation subtype

(A) Violin plot showing per-cell G3/G4 score (derived from projection onto scRNA-seq data) for 15 MB_{Grp3}/MB_{Grp4} patients aggregated by subtype.

(B) Ridge plot showing distribution of per-cell G3/G4 score (derived from projection onto scRNA-seq data) for each of 15 MB_{Grp3}/MB_{Grp4} patients shown alongside the G3/G4 score distribution of equivalent subtype bulk tumors. n = x refers to number of individuals for bulk tumors and number of cells for the scRNA-seq data. Vertical black lines indicate from left to right the fifth percentile, median, and 95th percentile. Dotted vertical lines denote the boundaries between highG4, lowG4, G3.5, lowG3, and highG3 (these categories are arbitrary divisions of the continuum for the purposes of visualization and comparison and do not represent "real" subgroups).

clear. The question remains as to what extent the intertumoral variation in such characteristics may be better explained by position upon the continuum than by methylation subtype. For at least some of these characteristics, those that are frequent and not specific to single subtypes (e.g., *MYC* amplification, LCA, i17q, gain of chromosome 5, loss of chromosome 8), it seems that they are more relatable to position on the continuum (Figures S4B and S4C).

The most striking association is between the G3/G4 continuum and risk of death, at least during the first 5 years post-diagnosis. Risk increases continuously with the G3/G4 continuum (Figure 2E), the documented phenomenon (Sharma et al., 2019) of late (>5 years post-diagnosis) relapse in subtype VIII notwithstanding. We regard this study as a description of an extremely close and therefore important relationship between biology and clinical course rather than as an advocation for its use as a clinical biomarker. Those judgments should be made using prospective clinical trials, and the cohort used here, while sizable and carefully reviewed, is a retrospective cohort with all of the limitations and caveats that implies. Nevertheless, we note that when it comes to incorporating molecular data into risk stratification schemes, the use of a single G3/G4 risk score for all MB_{Grp3}/MB_{Grp4} patients has a certain pragmatic logic over atomizing a rare cancer into 8 separate subtypes.

Pathway analysis of the G3/G4 continuum shows a concomitant activation of oncogenic processes (e.g., MYC, MTOR, TP53) as tumors become more MB_{Grp3}-like, which itself suggests a more aggressive phenotype. The influence of the G3/G4 continuum also extends to post-transcriptional regulation (i.e., isoform usage and RNA editing). Here, we describe log-linear relationships showing the primacy of the continuum in multiple aspects of $MB_{Grp3/Grp4}$ transcriptional biology. A close relationship with cell differentiation (e.g., CRX/NRL, neuronal differentiation) is also evident and consistent with previous descriptions of MB_{Grp3}/MB_{Grp4} biology cell identity and differentiation (Bandopadhayay et al., 2019; Garancher et al., 2018). This is further reflected in the progressive switches in methylation status that we observe within MB_{Grp3}/MB_{Grp4} specific enhancers (Lin et al., 2016).

We show here that the MB_{Grp3}/MB_{Grp4} continuum is produced by individual cells that themselves exist in the same expression continuum as the bulk tumors. In part, this was observed by Hovestadt et al. (2019) in their original analysis of their pooled MB_{Grp3}/MB_{Grp4} scRNA-seq data. They described two metagenes diverging according to *MYC* expression and described bulk tumors as composed of cells of either a predominately differentiated, undifferentiated, or intermediate type, which themselves represent a continuum of neuronal differentiation

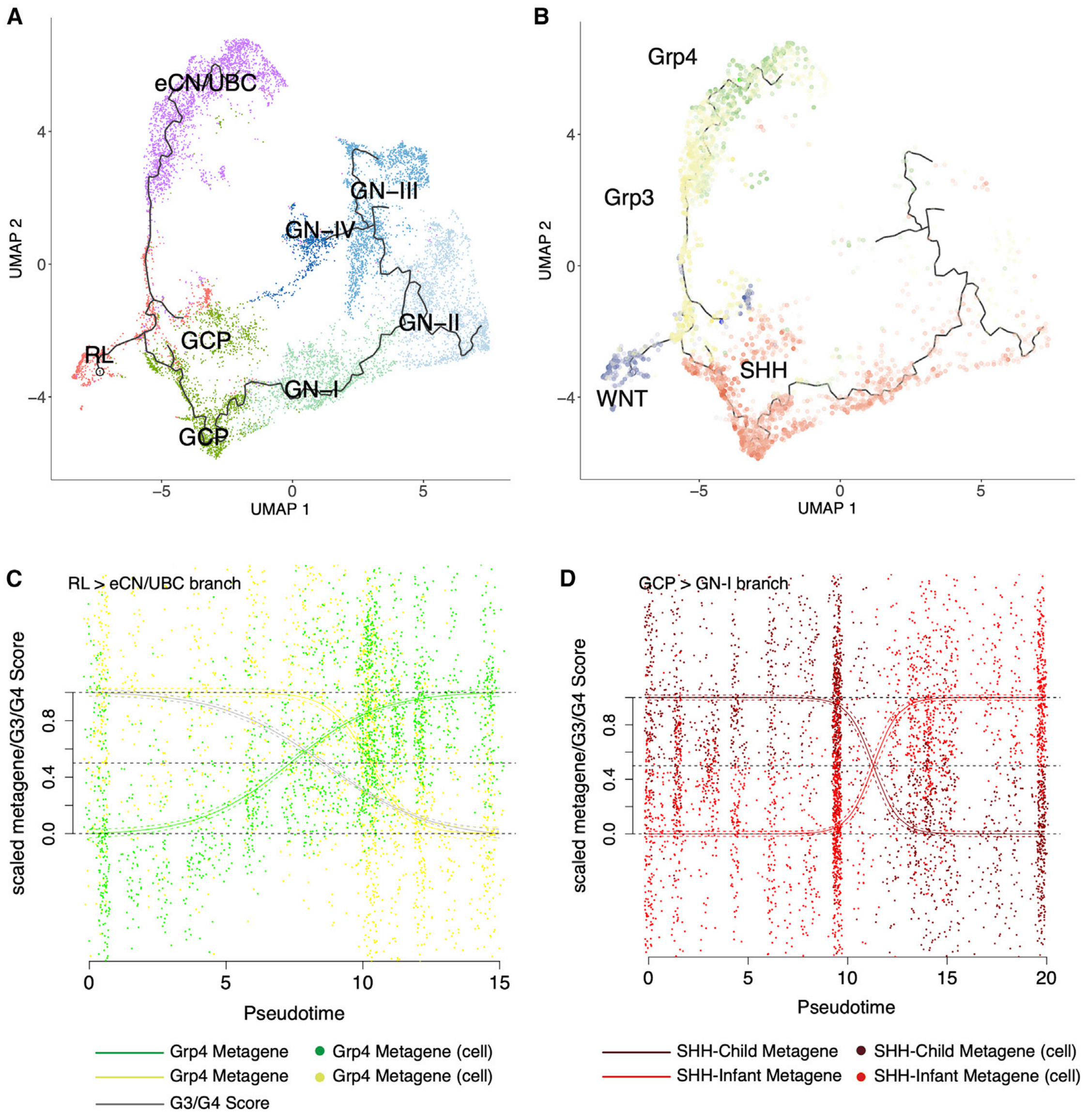

Figure 6. The group 3/group 4 continuum is mirrored in early human cerebellar development

(A) Uniform manifold approximation and projection (UMAP) plot of scRNA-seq profiles showing 12,243 cells of the RL lineage arranged according to developmental trajectory, which is indicated by the black line. Color denotes cell type as determined by graph-based clustering; RL, rhombic lip precursors; GCP, granule cell precursors; GN-I, GN-II, GN-III, GN-IV, 4 granule neuron cell types; eCN/UBC, excitatory cerebellar neurons/unipolar brush cells.

(B) UMAP plot of the RL lineage with those cells within the top decile of metagene expression marked with the following colors: MB_{Grp4}, green; MB_{Grp3}, yellow; MB_{SHH}, red; MB_{WNT}, blue.

(C) Scatterplot showing per-cell scaled metagene expression along the RL to eCN/UBC branch. Fitted sigmoid curves are shown, with SD indicated as dashed lines. The gray line represents a sigmoid curve fitted to per-cell G3/G4 score as a function of pseudotime.

(D) Scatterplot showing per-cell scaled metagene expression along the GCP to GN branch. Fitted curves are shown with SD shown as dashed lines. Curves are scaled to be constrained to a range of 0 and 1, to be coherent with bulk analysis. For this reason, by definition, some individual cells lie outside the 0 and 1 range.

(Hovestadt et al., 2019). We have expanded this by fitting individual cells onto the same metagene scale used to define the bulk tumor transcriptome, thereby defining more precisely the range of transcriptional intratumoral heterogeneity within MB_{Grp3}/MB_{Grp4} tumors and showing that it appears to be confined to certain limits prescribed by the MB_{Grp3}/MB_{Grp4} subtype. This in turn is consistent with the finding that MB sampled from different areas of the tumor or at diagnosis and relapse rarely alter subgroup (Kumar et al., 2021; Morrissy et al., 2016; Ramaswamy et al., 2013).

Unlike previous studies that attempted to define cells of origin, we used a human rather than a mouse scRNA-seq reference set for comparison. The use of a human atlas is significant because human RL persists longer through cerebellar development than the mouse and has unique cytoarchitectural features not shared with any other vertebrates (Haldipur et al., 2019). Mouse RL is a

CellPress
OPEN ACCESS

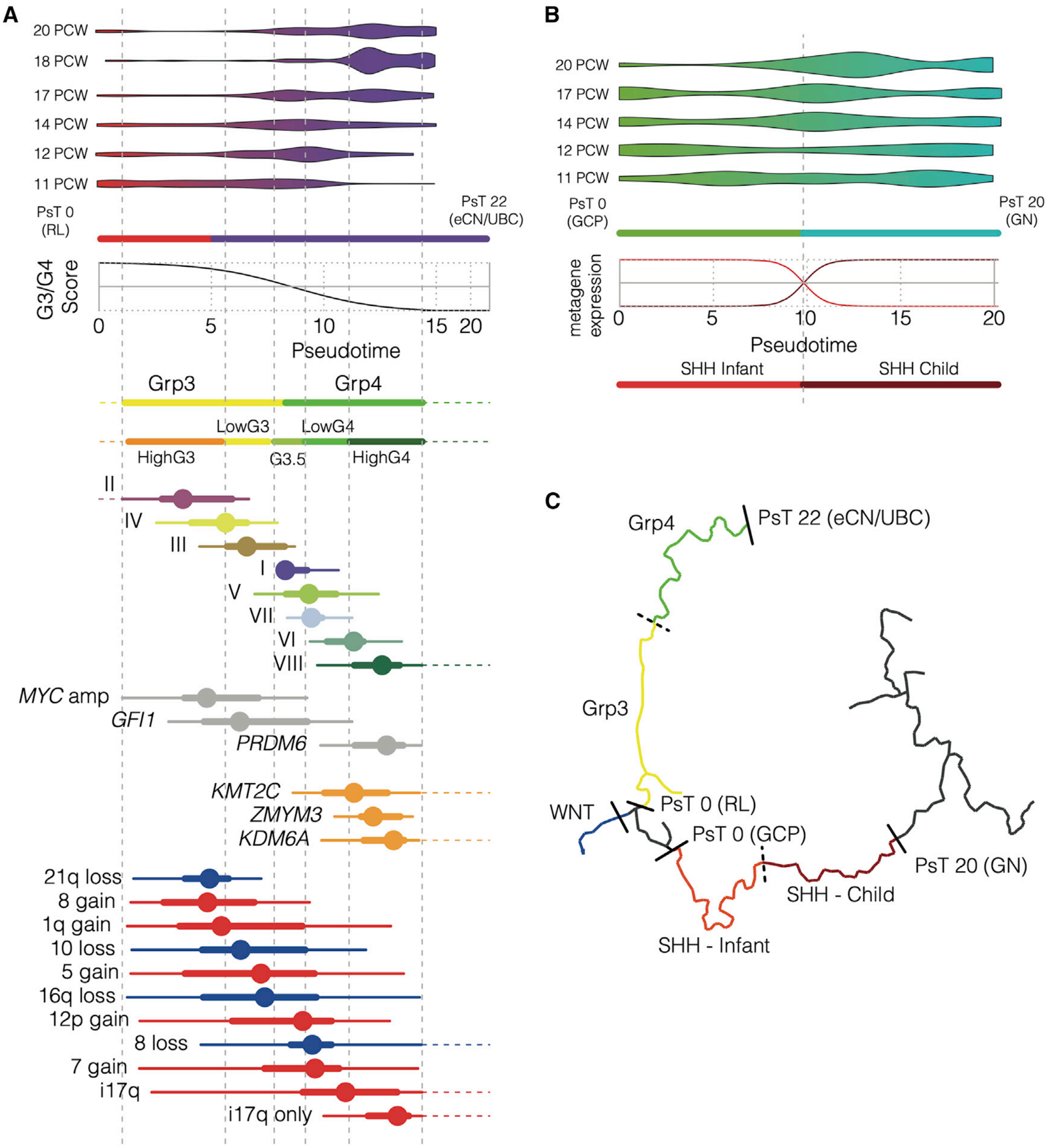

Figure 7. Key molecular characteristics of MB can be aligned to human fetal cerebellar developmental niches

(A) Schema showing the RL to eCN/UBC developmental branch, the relationship between pseudotime and G3/G4 score, and the staging of key tumor characteristics. From top to bottom: a violin plot showing pseudotime distribution of cells by time of sampling; color transition red to purple marks the point along the developmental trajectory at which cells are defined as eCN/UBC. A fitted sigmoid curve showing the relationship between pseudotime and G3/G4 score. Tumor characteristics are transformed from the G3/G4 scale to the pseudotime scale and marked at the appropriate points. Color bars represent subgroups. Methylation subtypes (I–VIII), mutations, and copy-number changes are marked by box and whisker. Dot represents median distribution; thick line represents the interquartile range; and the thinner lines correspond to range. Dotted horizontal lines denote where the range extends up to a G3/G4 score of 0 and 1 (i.e., matching the *ne plus ultra* pseudotime after which G3/G4 score is unchanged and exact relationship must be extrapolated). Dotted vertical lines denote the boundaries between highG4, lowG4, G3.5, lowG3, and highG3 (these categories are arbitrary divisions of the continuum for the purposes of visualization and comparison and do not represent “real” subgroups).

(B) Schematic showing the GCP to GN developmental branch and the relationship between pseudotime and $MB_{SHH\text{-}Infant}$ or $MB_{SHH\text{-}Child}$ metagene. From top to bottom: a violin plot showing pseudotime distribution of cells by time of sample; color transition green to blue marks the point along the developmental trajectory where cells become defined as GN. A loess curve shows the relationship between pseudotime and $MB_{SHH\text{-}Infant}$ (red) or $MB_{SHH\text{-}Child}$ metagene (dark red). Color bars show parts of trajectory paralleled by $MB_{SHH\text{-}Infant}$ or $MB_{SHH\text{-}Child}$ tumors.

(C) UMAP of developmental trajectory marked with colors to denote parts most associated with each MB subgroup and the relevant pseudotime (PsT) scale.

transient, proliferative stem cell zone present between embryonic day (E) E12.5 and E17.5, whereas human RL begins as a progenitor niche and is later compartmentalized into ventricular and subventricular zones, forming a human-specific progenitor pool within the posterior lobule, which persists until birth (Haldipur et al., 2019). We show that the MB_{Grp3}/MB_{Grp4} continuum is paralleled by a fetal cerebellar lineage that begins with an RL progenitor and ends with eCN/UBC. Aligning oncogenic features to windows within developmental pseudotemporal space suggests that cellular development/differentiation may be interrupted by oncogenic features at (or at least before) a certain point in the developmental trajectory. More speculatively, this may suggest a certain developmental pseudotemporal window of opportunity for specific oncogenic events to provoke MB of a given subtype. How or if this occurs would need to be modeled and tested through further functional experimentation. Nevertheless, we suggest that such future modeling efforts would be best directed to the appropriate window within the developmental trajectory, and we provide here a map to do so. We also demonstrate a putative relationship between earlier/later cell types and the age of onset of the disease. Importantly, we were able to identify a developmental niche for each of the four main MB subgroups including a separate space for $MB_{SHH\text{-}Child}$ and $MB_{SHH\text{-}Infant}$. Each of these is contained within a branch of the same early cerebellar lineage explicitly unifying each of the four subgroups to a common developmental antecedent, something not reported in previous studies. For instance, Hovestadt et al. (2019) were unable to identify a significant matching reference cell type for MB_{Grp3} and MB_{WNT}, whereas Vladoiu et al. (2019) did not analyze MB_{WNT} and note a prosaic resemblance of MB_{Grp3} to Nestin^{+} early neural stem-like cells.

In conclusion, our findings point to the following important insights. First, that group 3/group 4 MB and their methylation subtypes exist transcriptionally upon a continuum and that this is mirrored entirely by an equivalent continuum of transcriptional cell types in early human fetal cerebellar development. Second, that by using a human scRNA-seq reference, all four MB subtypes can be linked to a common developmental antecedent within the RL lineage. Third, that transcriptional intratumoral heterogeneity is limited to certain domains within the continuum as dictated by subtype. Finally, that the continuum is linked with almost every aspect of group 3/group 4 molecular biology and clinico-pathology. We anticipate this to have implications for the future treatment and modeling of the disease—most pressingly, a need to match cell type with specific timing of mutations to develop faithful models.

Limitations of the study

We wish to highlight the following, which we regard as some of the constraints and limitations of our study. In basing our conclusions upon a human developmental atlas, we note that we were selective, albeit based on prior knowledge, in the subset of cell types we considered to be potential candidate cells of origin—figuratively, by assigning them to what we broadly described as the RL lineage, and literally, by the physical process of cell extraction and the points in early human development for which sampling was possible (PCWs 9–21). MB_{WNT} in particular is thought to originate in the dorsal brainstem, and it may be that certain alternative cells of origin were excluded or curtailed on that basis. Nevertheless, previous studies follow a similar logic to our own and the coherent picture of the relationships between the subgroups would seem to bear out our choices. In addition, while we have aligned certain oncogenic features with specific developmental windows by virtue of their transcriptional resemblance, further functional experimentation will be required to determine if and how these oncogenic features provoke tumorigenesis specifically in these cell types.

We demonstrated a strong association between position on the G3/G4 continuum and risk of death. To what extent it may be effective and desirable to incorporate this into future clinical risk stratifications requires a more in-depth study, ideally as part of a prospective clinical trial. We have also touched upon the association between isoform expression or RNA editing and position on the G3/G4 continuum. We did this to demonstrate the primacy of the G3/G4 continuum in determining transcriptional biology; however, our description is by no means exhaustive and many important facets of MB RNA functional biology remain to be explored by future functional studies beyond the scope of the limited descriptions we have initiated here.

Finally, while we have demonstrated that the G3/G4 scores for individual cells appear to fall within a range on the continuum defined by the bulk tumors of the equivalent MB_{Grp3}/MB_{Grp4} subtypes (I–VIII), we should note that this was done with a relatively small number ($n = 15$) of individuals and that not all of the subtypes are covered equally. Further scRNA-seq analysis of individual MB_{Grp3}/MB_{Grp4} tumors should be undertaken to confirm the generalizability of this observation.

STAR★METHODS

Detailed methods are provided in the online version of this paper and include the following:

- KEY RESOURCES TABLE
- RESOURCE AVAILABILITY
  - Lead contact
  - Materials availability
  - Data and code availability
- EXPERIMENTAL MODEL AND SUBJECT DETAILS
  - Human tissue samples
- METHOD DETAILS
  - Patient samples and study cohort
  - RNA-seq analysis
- DNA METHYLATION ANALYSIS
- SCRNA-SEQ ANALYSIS
- QUANTIFICATION AND STATISTICAL ANALYSIS

SUPPLEMENTAL INFORMATION

Supplemental information can be found online at https://doi.org/10.1016/j.celrep.2022.111162.

ACKNOWLEDGMENTS

This work was supported by Cancer Research UK (C8464/A13457 and C8464/A23391), the Tom Graham Trust/CCLG, LoveOliver, Star for Harris and the

INSTINCT network, co-funded by The Brain Tumor Charity, Great Ormond Street Children's Charity, and Children with Cancer UK (grant no. 16/193). T.S.J. is grateful for additional funding from the Olivia Hodson Cancer Fund, Cancer Research UK, and the National Institute of Health Research. All of the research conducted at Great Ormond Street Hospital NHS Foundation Trust and UCL Great Ormond Street Institute of Child Health is made possible by the NIHR Great Ormond Street Hospital Biomedical Research Center. The views expressed are those of the authors and not necessarily those of the NHS, the NIHR or the Department of Health.

AUTHOR CONTRIBUTIONS

Conceptualization, D.W., E.C.S., S.B., and S.C.C; methodology, D.W., E.C.S., S.B., and S.C.C; formal analysis, D.W., E.C.S., K.A.A., and Y.G.; investigation, D.W., E.C.S., D.H., K.A.A., J.C.L., S.C., S.R., J.G., R.M.H., J.C., S.B.W., T.S.J., A.J., S.B., and S.C.C.; resources, K.A.A., B.P., S.B.W., T.S.J., and A.J.; data curation, D.W., E.C.S., D.H., J.C.L., S.R., J.G., R.M.H., J.C., Y.G., and J.H.; writing – original draft, D.W., E.C.S., and S.C.C.; writing – review & editing, D.H., K.A.A., S.R., J.G., R.M.H., B.P., and S.B.; visualization, D.W. and J.H.; supervision, D.W., S.B., and S.C.C.; funding acquisition, D.W., S.B., and S.C.C. All of the authors approved the final version of the manuscript.

Received: July 26, 2021
Revised: May 26, 2022
Accepted: July 13, 2022
Published: August 2, 2022

REFERENCES

Anders, S., Pyl, P.T., and Huber, W. (2015). HTSeq—a Python framework to work with high-throughput sequencing data. Bioinformatics *31*, 166–169. https://doi.org/10.1093/bioinformatics/btu638.

Aryee, M.J., Jaffe, A.E., Corrada-Bravo, H., Ladd-Acosta, C., Feinberg, A.P., Hansen, K.D., and Irizarry, R.A. (2014). Minfi: a flexible and comprehensive Bioconductor package for the analysis of Infinium DNA methylation microarrays. Bioinformatics *30*, 1363–1369. https://doi.org/10.1093/bioinformatics/btu049.

Bandopadhayay, P., Piccioni, F., O'Rourke, R., Ho, P., Gonzalez, E.M., Buchan, G., Qian, K., Gionet, G., Girard, E., Coxon, M., et al. (2019). Neuronal differentiation and cell-cycle programs mediate response to BET-bromodomain inhibition in MYC-driven medulloblastoma. Nat. Commun. *10*, 2400–2416. https://doi.org/10.1038/s41467-019-10307-9.

Bray, N.L., Pimentel, H., Melsted, P., and Pachter, L. (2016). Near-optimal probabilistic RNA-seq quantification. Nat. Biotechnol. *34*, 525–527. https://doi.org/10.1038/nbt.3519.

Butler, A., Hoffman, P., Smibert, P., Papalexi, E., and Satija, R. (2018). Integrating single-cell transcriptomic data across different conditions, technologies, and species. Nat. Biotechnol. *36*, 411–420. https://doi.org/10.1038/nbt.4096.

Capper, D., Jones, D.T.W., Sill, M., Hovestadt, V., Schrimpf, D., Sturm, D., Koelsche, C., Sahm, F., Chavez, L., Reuss, D.E., and Kratz, A. (2018). DNA methylation-based classification of central nervous system tumours. Nature *555*, 469–474. https://doi.org/10.1038/nature26000.

Cavalli, F.M.G., Remke, M., Rampasek, L., Peacock, J., Shih, D.J.H., Luu, B., Garzia, L., Torchia, J., Nor, C., Morrissy, A.S., et al. (2017). Intertumoral heterogeneity within medulloblastoma subgroups. Cancer Cell *31*, 737–754.e6. https://doi.org/10.1016/j.ccell.2017.05.005.

Chang, C.H., Housepian, E.M., and Herbert, C. (1969). An operative staging system and a megavoltage radiotherapeutic technic for cerebellar medulloblastomas. Radiology *93*, 1351–1359. https://doi.org/10.1148/93.6.1351.

Chen, L., Li, Y., Lin, C.H., Chan, T.H.M., Chow, R.K.K., Song, Y., Liu, M., Yuan, Y.-F., Fu, L., Kong, K.L., et al. (2013). Recoding RNA editing of AZIN1 predisposes to hepatocellular carcinoma. Nat. Med. *19*, 209–216. https://doi.org/10.1038/nm.3043.

Cho, Y.-J., Tsherniak, A., Tamayo, P., Santagata, S., Ligon, A., Greulich, H., Berhoukim, R., Amani, V., Goumnerova, L., Eberhart, C.G., et al. (2011). Integrative genomic analysis of medulloblastoma identifies a molecular subgroup that drives poor clinical outcome. J. Clin. Oncol. *29*, 1424–1430. https://doi.org/10.1200/JCO.2010.28.5148.

Clifford, S.C., Lusher, M.E., Lindsey, J.C., Langdon, J.A., Gilbertson, R.J., Straughton, D., and Elliso, D.W. (2006). Wnt/Wingless pathway activation and chromosome 6 loss characterize a distinct molecular sub-group of medulloblastomas associated with a favorable prognosis. Cell Cycle *5*, 2666–2670.

Dobin, A., Davis, C.A., Schlesinger, F., Drenkow, J., Zaleski, C., Jha, S., Batut, P., Chaisson, M., and Gingeras, T.R. (2013). STAR: ultrafast universal RNA-seq aligner. Bioinformatics *29*, 15–21. https://doi.org/10.1093/bioinformatics/bts635.

Ellison, D.W., Onilude, O.E., Lindsey, J.C., Lusher, M.E., Weston, C.L., Taylor, R.E., Pearson, A.D., and Clifford, S.C.; United Kingdom Children's Cancer Study Group Brain Tumour Committee (2005). Beta-catenin status predicts a favorable outcome in childhood medulloblastoma: the United Kingdom children's cancer study group brain Tumour committee. J. Clin. Oncol. *23*, 7951–7957. https://doi.org/10.1200/JCO.2005.01.5479.

Fattet, S., Haberler, C., Legoix, P., Varlet, P., Lellouch-Tubiana, A., Lair, S., Manie, E., Raquin, M.-A., Bours, D., Carpentier, S., et al. (2009). Beta-catenin status in paediatric medulloblastomas: correlation of immunohistochemical expression with mutational status, genetic profiles, and clinical characteristics. J. Pathol. *218*, 86–94. https://doi.org/10.1002/path.2514.

Garancher, A., Lin, C.Y., Morabito, M., Richer, W., Rocques, N., Larcher, M., Bihannic, L., Smith, K., Miquel, C., Leboucher, S., et al. (2018). NRL and CRX define photoreceptor identity and reveal subgroup-specific dependencies in medulloblastoma. Cancer Cell *33*, 435–449.e6. https://doi.org/10.1016/j.ccell.2018.02.006.

Gibson, P., Tong, Y., Robinson, G., Thompson, M.C., Currle, D.S., Eden, C., Kranenburg, T.A., Hogg, T., Poppleton, H., Martin, J., et al. (2010). Subtypes of medulloblastoma have distinct developmental origins. Nature *468*, 1095–1099. https://doi.org/10.1038/nature09587.

Haldipur, P., Aldinger, K.A., Bernardo, S., Deng, M., Timms, A.E., Overman, L.M., Winter, C., Lisgo, S.N., Razavi, F., Silvestri, E., et al. (2019). Spatiotemporal expansion of primary progenitor zones in the developing human cerebellum. Science *366*, 454–460. https://doi.org/10.1126/science.aax7526.

Hänzelmann, S., Castelo, R., and Guinney, J. (2013). GSVA: gene set variation analysis for microarray and RNA-seq data. BMC Bioinf. *14*, 7–15. https://doi.org/10.1186/1471-2105-14-7.

Hovestadt, V., Jones, D.T.W., Picelli, S., Wang, W., Kool, M., Northcott, P.A., Sultan, M., Stachurski, K., Ryzhova, M., Warnatz, H.-J., et al. (2014). Decoding the regulatory landscape of medulloblastoma using DNA methylation sequencing. Nature *510*, 537–541. https://doi.org/10.1038/nature13268.

Hovestadt, V., Smith, K.S., Bihannic, L., Filbin, M.G., Shaw, M.L., Baumgartner, A., DeWitt, J.C., Groves, A., Mayr, L., Weisman, H.R., et al. (2019). Resolving medulloblastoma cellular architecture by single-cell genomics. Nature *572*, 74–79. https://doi.org/10.1038/s41586-019-1434-6.

Hu, X., Chen, J., Shi, X., Feng, F., Lau, K.W., Chen, Y., Chen, Y., Jiang, L., Cui, F., Zhang, Y., et al. (2017). RNA editing of AZIN1 induces the malignant progression of non-small-cell lung cancers. Tumour Biol. *39*, 1010428317700001. https://doi.org/10.1177/1010428317700001.

Kool, M., Koster, J., Bunt, J., Hasselt, N.E., Lakeman, A., van Sluis, P., Troost, D., Meeteren, N.S.-V., Caron, H.N., Cloos, J., et al. (2008). Integrated genomics identifies five medulloblastoma subtypes with distinct genetic profiles, pathway signatures and clinicopathological features. PLoS One *3*, e3088. https://doi.org/10.1371/journal.pone.0003088.

Kool, M., Korshunov, A., Remke, M., Jones, D.T.W., Schlanstein, M., Northcott, P.A., Cho, Y.-J., Koster, J., Schouten-van Meeteren, A., van Vuurden, D., et al. (2012). Molecular subgroups of medulloblastoma: an international meta-analysis of transcriptome, genetic aberrations, and clinical data of WNT, SHH, Group 3, and Group 4 medulloblastomas. Acta Neuropathol. *123*, 473–484. https://doi.org/10.1007/s00401-012-0958-8.

Kool, M., Jones, D.T.W., Jäger, N., Northcott, P.A., Pugh, T.J., Hovestadt, V., Piro, R.M., Esparza, L.A., Markant, S.L., Remke, M., et al. (2014). Genome sequencing of SHH medulloblastoma predicts genotype-related response to smoothened inhibition. Cancer Cell *25*, 393–405. https://doi.org/10.1016/j.ccr.2014.02.004.

Kumar, R., Smith, K.S., Deng, M., Terhune, C., Robinson, G.W., Orr, B.A., Liu, A.P.Y., Lin, T., Billups, C.A., Chintagumpala, M., et al. (2021). Clinical outcomes and patient-matched molecular composition of relapsed medulloblastoma. J. Clin. Oncol. *39*, 807–821. https://doi.org/10.1200/JCO.20.01359.

Li, Y., Song, Q., and Day, B.W. (2019). Phase I and phase II sonidegib and vismodegib clinical trials for the treatment of paediatric and adult MB patients: a systemic review and meta-analysis. Acta Neuropathol. Commun. *7*, 123–128. https://doi.org/10.1186/s40478-019-0773-8.

Lin, C.Y., Erkek, S., Tong, Y., Yin, L., Federation, A.J., Zapatka, M., Haldipur, P., Kawauchi, D., Risch, T., Warnatz, H.-J., et al. (2016). Active medulloblastoma enhancers reveal subgroup-specific cellular origins. Nature *530*, 57–62. https://doi.org/10.1038/nature16546.

Lo Giudice, C., Silvestris, D.A., Roth, S.H., Eisenberg, E., Pesole, G., Gallo, A., and Picardi, E. (2020). Quantifying RNA editing in deep transcriptome datasets. Front. Genet. *11*, 194. https://doi.org/10.3389/fgene.2020.00194.

Lopes, M.C., Joyce, C., Ritchie, G.R.S., John, S.L., Cunningham, F., Asimit, J., and Zeggini, E. (2012). A combined functional annotation score for non-synonymous variants. Hum. Hered. *73*, 47–51. https://doi.org/10.1159/000334984.

Louis, D.N., Perry, A., Reifenberger, G., von Deimling, A., Figarella-Branger, D., Cavenee, W.K., Ohgaki, H., Wiestler, O.D., Kleihues, P., and Ellison, D.W. (2016). The 2016 world Health organization classification of tumors of the central nervous system: a summary. Acta Neuropathol. *131*, 803–820. https://doi.org/10.1007/s00401-016-1545-1.

Louis, D.N., Perry, A., Wesseling, P., Brat, D.J., Cree, I.A., Figarella-Branger, D., Hawkins, C., Ng, H.K., Pfister, S.M., Reifenberger, G., et al. (2021). The 2021 WHO classification of tumors of the central nervous system: a summary. Neuro Oncol. *23*, 1231–1251. https://doi.org/10.1093/neuonc/noab106.

Love, M.I., Huber, W., and Anders, S. (2014). Moderated estimation of fold change and dispersion for RNA-seq data with DESeq2. Genome Biol. *15*, 550. https://doi.org/10.1186/s13059-014-0550-8.

McLaren, W., Gil, L., Hunt, S.E., Riat, H.S., Ritchie, G.R.S., Thormann, A., Flicek, P., and Cunningham, F. (2016). The Ensembl variant effect predictor. Genome Biol. *17*, 122–214. https://doi.org/10.1186/s13059-016-0974-4.

Morrissy, A.S., Garzia, L., Shih, D.J.H., Zuyderduyn, S., Huang, X., Skowron, P., Remke, M., Cavalli, F.M.G., Ramaswamy, V., Lindsay, P.E., et al. (2016). Divergent clonal selection dominates medulloblastoma at recurrence. Nature *529*, 351–357. https://doi.org/10.1038/nature16478.

Northcott, P.A., Korshunov, A., Witt, H., Hielscher, T., Eberhart, C.G., Mack, S., Bouffet, E., Clifford, S.C., Hawkins, C.E., French, P., et al. (2011). Medulloblastoma comprises four distinct molecular variants. J. Clin. Oncol. *29*, 1408–1414. https://doi.org/10.1200/JCO.2009.27.4324.

Northcott, P.A., Jones, D.T.W., Kool, M., Robinson, G.W., Gilbertson, R.J., Cho, Y.-J., Pomeroy, S.L., Korshunov, A., Lichter, P., Taylor, M.D., and Pfister, S.M. (2012). Medulloblastomics: the end of the beginning. Nat. Rev. Cancer *12*, 818–834. https://doi.org/10.1038/nrc3410.

Northcott, P.A., Lee, C., Zichner, T., Stütz, A.M., Erkek, S., Kawauchi, D., Shih, D.J.H., Hovestadt, V., Zapatka, M., Sturm, D., et al. (2014). Enhancer hijacking activates GFI1 family oncogenes in medulloblastoma. Nature *511*, 428–434. https://doi.org/10.1038/nature13379.

Northcott, P.A., Buchhalter, I., Morrissy, A.S., Hovestadt, V., Weischenfeldt, J., Ehrenberger, T., Gröbner, S., Segura-Wang, M., Zichner, T., Rudneva, V.A., et al. (2017). The whole-genome landscape of medulloblastoma subtypes. Nature *547*, 311–317. https://doi.org/10.1038/nature22973.

Okugawa, Y., Toiyama, Y., Shigeyasu, K., Yamamoto, A., Shigemori, T., Yin, C., Ichikawa, T., Yasuda, H., Fujikawa, H., Yoshiyama, S., et al. (2018). Enhanced AZIN1 RNA editing and overexpression of its regulatory enzyme ADAR1 are important prognostic biomarkers in gastric cancer. J. Transl. Med. *16*, 366–411. https://doi.org/10.1186/s12967-018-1740-z.

Peters, T.J., Buckley, M.J., Statham, A.L., Pidsley, R., Samaras, K., Lord, R.V., Clark, S.J., and Molloy, P.L. (2015). De novo identification of differentially methylated regions in the human genome. Epigenetics & Chromatin *8*, 6. http://www.epigeneticsandchromatin.com/content/8/1/6.

Peterson, R.A. (2021). Finding Optimal Normalizing Transformations via bestNormalize. The R Journal *13*, 310–329. https://doi.org/10.32614/RJ-2021-041.

Qiu, X., Hill, A., Packer, J., Lin, D., Ma, Y.-A., and Trapnell, C. (2017). Single-cell Mrna quantification and differential analysis with Census. Nat. Methods *14*, 309–315. https://doi.org/10.1038/nmeth.4150.

Ramaswamy, V., Remke, M., Bouffet, E., Faria, C.C., Perreault, S., Cho, Y.-J., Shih, D.J., Luu, B., Dubuc, A.M., Northcott, P.A., et al. (2013). Recurrence patterns across medulloblastoma subgroups: an integrated clinical and molecular analysis. Lancet Oncol. *14*, 1200–1207. https://doi.org/10.1016/S1470-2045(13)70449-2.

Ritchie, M.E., Phipson, B., Wu, D., Hu, Y., Law, C.W., Shi, W., and Smyth, G.K. (2015). limma powers differential expression analyses for RNA-sequencing and microarray studies. Nucleic Acids Research *43*, e47. https://doi.org/10.1093/nar/gkv007.

Robinson, J.T., Thorvaldsdóttir, H., Winckler, W., Guttman, M., Lander, E.S., Getz, G., and Mesirov, J.P. (2011). Integrative genomics viewer. Nat. Biotechnol. *29*, 24–26. https://doi.org/10.1038/nbt.1754.

Robinson, G.W., Orr, B.A., Wu, G., Gururangan, S., Lin, T., Qaddoumi, I., Packer, R.J., Goldman, S., Prados, M.D., Desjardins, A., et al. (2015). Vismodegib exerts targeted efficacy against recurrent sonic hedgehog-subgroup medulloblastoma: results from phase II pediatric brain tumor consortium studies PBTC-025B and PBTC-032. J. Clin. Oncol. *33*, 2646–2654. https://doi.org/10.1200/JCO.2014.60.1591.

Ryan, S.L., Schwalbe, E.C., Cole, M., Lu, Y., Lusher, M.E., Megahed, H., O'Toole, K., Nicholson, S.L., Bognár, L., Garami, M., et al. (2012). MYC family amplification and clinical risk-factors interact to predict an extremely poor prognosis in childhood medulloblastoma. Acta Neuropathol. *123*, 501–513. https://doi.org/10.1007/s00401-011-0923-y.

Schwalbe, E.C., Williamson, D., Lindsey, J.C., Hamilton, D., Ryan, S.L., Megahed, H., Garami, M., Hauser, P., Dembowska-Baginska, B., Perek, D., et al. (2013). DNA methylation profiling of medulloblastoma allows robust subclassification and improved outcome prediction using formalin-fixed biopsies. Acta Neuropathol. *125*, 359–371. https://doi.org/10.1007/s00401-012-1077-2.

Schwalbe, E.C., Lindsey, J.C., Nakjang, S., Crosier, S., Smith, A.J., Hicks, D., Rafiee, G., Hill, R.M., Iliasova, A., Stone, T., et al. (2017). Molecular subgroups for clinical classification and outcome prediction in childhood medulloblastoma: a cohort study. Lancet Oncol. *18*, 958–971. https://doi.org/10.1016/S1470-2045(17)30243-7.

Scrucca, L., Fop, M., Murphy, T.B., and Raftery, A.E. (2016). mclust 5: clustering, classification and density estimation using Gaussian finite mixture models. The R Journal *8*, 289–317. https://doi.org/10.32614/RJ-2016-021.

Sharma, T., Schwalbe, E.C., Williamson, D., Sill, M., Hovestadt, V., Mynarek, M., Rutkowski, S., Robinson, G.W., Gajjar, A., Cavalli, F., et al. (2019). Second-generation molecular subgrouping of medulloblastoma: an international meta-analysis of Group 3 and Group 4 subtypes. Acta Neuropathol. *138*, 309–326. https://doi.org/10.1007/s00401-019-02020-0.

Shigeyasu, K., Okugawa, Y., Toden, S., Miyoshi, J., Toiyama, Y., Nagasaka, T., Takahashi, N., Kusunoki, M., Takayama, T., Yamada, Y., et al. (2018). AZIN1 RNA editing confers cancer stemness and enhances oncogenic potential in colorectal cancer. JCI Insight *3*, 99976. https://doi.org/10.1172/jci.insight.99976.

Shihab, H.A., Gough, J., Cooper, D.N., Stenson, P.D., Barker, G.L.A., Edwards, K.J., Day, I.N.M., and Gaunt, T.R. (2013). Predicting the functional, molecular, and phenotypic consequences of amino acid substitutions using hidden Markov models. Hum. Mutat. *34*, 57–65. https://doi.org/10.1002/humu.22225.

Shirai, Y., Watanabe, M., Sakagami, H., and Suzuki, T. (2015. Novel). Splice variants in the 5"UTR of Gtf2i expressed in the rat brain: alternative 5"UTRs

CellPress
OPEN ACCESS

and differential expression in the neuronal dendrites. J. Neurochem. *134*, 578–589. https://doi.org/10.1111/jnc.13136.

Shirai, Y., Li, W., and Suzuki, T. (2017). Role of splice variants of Gtf2i, a transcription factor localizing at postsynaptic sites, and its relation to neuropsychiatric diseases. Int. J. Mol. Sci. *18*, 411. https://doi.org/10.3390/ijms18020411.

Soneson, C., Love, M.I., and Robinson, M.D. (2015). Differential analyses for RNA-seq: transcript-level estimates improve gene-level inferences. F1000Research *4*. https://doi.org/10.12688/f1000research.7563.1.

Tamayo, P., Scanfeld, D., Ebert, B.L., Gillette, M.A., Roberts, C.W.M., and Mesirov, J.P. (2007). Metagene projection for cross-platform, cross-species characterization of global transcriptional states. Proc. Natl. Acad. Sci. USA *104*, 5959–5964. https://doi.org/10.1073/pnas.0701068104.

Taylor, M.D., Northcott, P.A., Korshunov, A., Remke, M., Cho, Y.-J., Clifford, S.C., Eberhart, C.G., Parsons, D.W., Rutkowski, S., Gajjar, A., et al. (2012). Molecular subgroups of medulloblastoma: the current consensus. Acta Neuropathol. *123*, 465–472. https://doi.org/10.1007/s00401-011-0922-z.

Thompson, M.C., Fuller, C., Hogg, T.L., Dalton, J., Finkelstein, D., Lau, C.C., Chintagumpala, M., Adesina, A., Ashley, D.M., Kellie, S.J., et al. (2006). Genomics identifies medulloblastoma subgroups that are enriched for specific genetic alterations. J. Clin. Oncol. *24*, 1924–1931. https://doi.org/10.1200/JCO.2005.04.4974.

Van der Auwera, G.A., Carneiro, M.O., Hartl, C., Poplin, R., del Angel, G., Levy-Moonshine, A., Jordan, T., Shakir, K., Roazen, D., Thibault, J., et al. (2013). From FastQ data to high confidence variant calls: the Genome Analysis Toolkit best practices pipeline. Curr. Protoc. Bioinf. *43*, 11.10.1–11.10.33. https://doi.org/10.1002/0471250953.bi1110s43.

Venables, W.N., and Ripley, B.D. (2002). Modern Applied Statistics with S, Fourth edition (Springer). https://www.stats.ox.ac.uk/pub/MASS4/.

Vladoiu, M.C., El-Hamamy, I., Donovan, L.K., Farooq, H., Holgado, B.L., Sundaravadanam, Y., Ramaswamy, V., Hendrikse, L.D., Kumar, S., Mack, S.C., et al. (2019). Childhood cerebellar tumours mirror conserved fetal transcriptional programs. Nature *572*, 67–73. https://doi.org/10.1038/s41586-019-1158-7.

STAR★METHODS

KEY RESOURCES TABLE

REAGENT or RESOURCE	SOURCE	IDENTIFIER
**Biological samples**		
Snap frozen medulloblastoma	CCLG Biobank/ Biological study and collaborating centres	See Table S1
**Chemicals, peptides, and recombinant proteins**		
Trizol	Thermo Fisher	15596026
1 X Low TE Buffer (10 mM Tris-HCL, ph 7.5-8.0, 0.1 mM EDTA)	Thermo Fisher	120900915
100% Ethanol, molecular biology grade	Sigma-Aldrich	E7023
Nuclease-free Water	Thermo Fisher	AM9930
**Critical commercial assays**		
RNeasy MinElute Cleanup Kit	Qiagen	74204
DNeasy Blood and Tissue Kit	Qiagen	69504
Agilent SureSelect XT2	Agilent	G9621A
Agilent SureSelect XTHS (Low Input)	Agilent	G9703A
Agilent SureSelect Custom DNA Target Enrichment Probes Tier 1 (500Kb)	Agilent	5190-4813
Afilent SureSelect XT Human All Exon v6 + UTR	Agilent	5190-8881
AMPPure XP Kit	Beckman Coulter	A63880
Herculase II Fusion DNA Polymerase	Agilent	600677
Dynabeads MyOne Streptavidin T1	Thermo Fisher	65601
Qubit dsDNA HS Assay Kit	Thermo Fisher	Q32851
**Deposited data**		
Medulloblastoma methylation array dataset E-MTAB-10754	This paper	Array Express: E-MTAB-10754
Medulloblastoma RNA-seq dataset E-MTAB-10767	This paper	Array Express: E-MTAB-10767
Medulloblastoma Methylation array dataset GSE130051	Sharma et al. 2019	GEO accession: GSE130051
Medulloblastoma Methylation array dataset GSE93646	Schwalbe et al., 2017	GEO accession: GSE93646
Medulloblastoma scRNA-seq dataset GSE119926	Hovestadt et al., 2019	GEO accession: GSE119926
Human fetal cerebellum scRNA-seq dataset	Human Cell Atlas (https://www.covid19cellatlas.org/aldinger20)	dbGAP accession: phs001908.v2.p1
**Software and algorithms**		
R v3.5.3 & v4.0.2 & R base packages	https://www.r-project.org	N/A
Bioconductor	http://bioconductor.org	NA
Kallisto v0.46.0	Bray, N., Pimentel, H., Melsted, P. et al. Near-optimal probabilistic RNA-seq quantification. Nat Biotechnol 34, 525–527 (2016). https://doi.org/10.1038/nbt.3519	N/A

(Continued on next page)

Continued

REAGENT or RESOURCE	SOURCE	IDENTIFIER
RNA-STAR v2.7.0e	Alexander Dobin, Carrie A. Davis, Felix Schlesinger, Jorg Drenkow, Chris Zaleski, Sonali Jha, Philippe Batut, Mark Chaisson, Thomas R. Gingeras, STAR: ultrafast universal RNA-seq aligner, Bioinformatics, Volume 29, Issue 1, January 2013, Pages 15–21, https://doi.org/10.1093/bioinformatics/bts635	NA
HTSeq v0.9.1	G Putri, S Anders, PT Pyl, JE Pimanda, F Zanini Analysing high-throughput sequencing data in Python with HTSeq 2.0 https://doi.org/10.1093/bioinformatics/btac166 (2022)	N/A
SAMtools v1.9,	Heng Li, Bob Handsaker, Alec Wysoker, Tim Fennell, Jue Ruan, Nils Homer, Gabor Marth, Goncalo Abecasis, Richard Durbin, 1000 Genome Project Data Processing Subgroup, The Sequence Alignment/Map format and SAMtools, Bioinformatics, Volume 25, Issue 16, 15 August 2009, Pages 2078–2079, https://doi.org/10.1093/bioinformatics/btp352	N/A
Picard v2.2.4	https://github.com/broadinstitute/picard	N/A
QEdit	https://github.com/BioinfoUNIBA/QEdit	N/A
Genome Analysis Toolkit (GATK) version 3.7	McKenna A, Hanna M, Banks E, Sivachenko A, Cibulskis K, Kernytsky A, Garimella K, Altshuler D, Gabriel S, Daly M, DePristo MA. (2010). The Genome Analysis Toolkit: a MapReduce framework for analyzing next-generation DNA sequencing data. Genome Res, 20:1297-303. https://doi.org/10.1101/gr.107524.110.	N/A
Ensembl Variant Effect Predictor (VEP)	McLaren, W., Gil, L., Hunt, S.E. et al. The Ensembl Variant Effect Predictor. Genome Biol 17, 122 (2016). https://doi.org/10.1186/s13059-016-0974-4	N/A
DESeq2_1.22.2	Love MI, Huber W, Anders S (2014). “Moderated estimation of fold change and dispersion for RNA-seq data with DESeq2.” Genome Biology, 15, 550. https://doi.org/10.1186/s13059-014-0550-8.	N/A
minfi_1.28.4	Fortin J, Triche TJ, Hansen KD (2017). “Preprocessing, normalization and integration of the Illumina HumanMethylationEPIC array with minfi.” Bioinformatics, 33(4). https://doi.org/10.1093/bioinformatics/btw691.	N/A
NMF_0.23.0	Gaujoux R, Seoighe C (2010). “A flexible R package for nonnegative matrix factorization.” BMC Bioinformatics, 11(1), 367. ISSN 1471-2105, https://doi.org/10.1186/1471-2105-11-367, https://bmcbioinformatics.biomedcentral.com/articles/10.1186/1471-2105-11-367.	N/A
limma_3.38.3	Ritchie et al, 2015, Ritchie ME, Phipson B, Wu D, Hu Y, Law CW, Shi W, Smyth GK (2015). “limma powers differential expression analyses for RNA-sequencing and microarray studies.” Nucleic Acids Research, 43(7), e47. https://doi.org/10.1093/nar/gkv007.	N/A
sva_3.36.0	https://bioconductor.org/packages/release/bioc/html/sva.html	N/A
tximport_1.10.1	Soneson et al., 2015, Soneson C, Love MI, Robinson MD (2015). “Differential analyses for RNA-seq: transcript-level estimates improve gene-level inferences.” F1000Research, 4. https://doi.org/10.12688/f1000research.7563.1.	N/A

(*Continued on next page*)

Continued

REAGENT or RESOURCE	SOURCE	IDENTIFIER
tximportData_1.10.0	Soneson et al., 2015, Soneson C, Love MI, Robinson MD (2015). "Differential analyses for RNA-seq: transcript-level estimates improve gene-level inferences." F1000Research, 4. https://doi.org/10.12688/f1000research.7563.1.	N/A
caret_6.0–86	cran package: https://cran.r-project.org/web/packages/available_packages_by_name.html	N/A
DMRcate_1.18.0	Peters et al., 2015, Peters TJ, Buckley MJ, Statham AL, Pidsley R, Samaras K, Lord RV, Clark SJ, Molloy PL (2015). "De novo identification of differentially methylated regions in the human genome." Epigenetics & Chromatin, 8, 6. http://www.epigeneticsandchromatin.com/content/8/1/6.	N/A
Rtsne_0.15	cran package: https://github.com/jkrijthe/Rtsne	N/A
biomaRt_2.38.0	Durinck S, Spellman P, Birney E, Huber W (2009). "Mapping identifiers for the integration of genomic datasets with the R/Bioconductor package biomaRt." Nature Protocols, 4, 1184–1191.	N/A
ggplot2_3.3.2	Wickham, H., 2016. ggplot2: Elegant Graphics for Data Analysis, Springer-Verlag New York. Available at: https://ggplot2.tidyverse.org	N/A
SingleCellExperiment_1.4.1	Amezquita R, Lun A, Becht E, Carey V, Carpp L, Geistlinger L, Marini F, Rue-Albrecht K, Risso D, Soneson C, Waldron L, Pages H, Smith M, Huber W, Morgan M, Gottardo R, Hicks S (2020). "Orchestrating single-cell analysis with Bioconductor." Nature Methods, 17, 137–145. https://www.nature.com/articles/s41592-019-0654-x.	N/A
Seurat_3.2.0	Stuart T, Butler A, Hoffman P, Hafemeister C, Papalexi E, III WMM, Hao Y, Stoeckius M, Smibert P, Satija R (2019). "Comprehensive Integration of Single-Cell Data." Cell, 177, 1888-1902. https://doi.org/10.1016/j.cell.2019.05.031, https://doi.org/10.1016/j.cell.2019.05.031.	N/A
survival_3.2–7	https://cran.r-project.org/web/packages/survival/index.html	N/A
tidyverse_1.3.0	Wickham H, Averick M, Bryan J, Chang W, McGowan LD, François R, Grolemund G, Hayes A, Henry L, Hester J, Kuhn M, Pedersen TL, Miller E, Bache SM, Müller K, Ooms J, Robinson D, Seidel DP, Spinu V, Takahashi K, Vaughan D, Wilke C, Woo K, Yutani H (2019). "Welcome to the tidyverse." Journal of Open Source Software, 4(43), 1686. https://doi.org/10.21105/joss.01686.	N/A
mclust_5.4.6	Scrucca et al., 2016, Scrucca L, Fop M, Murphy TB, Raftery AE (2016). "mclust 5: clustering, classification and density estimation using Gaussian finite mixture models." The R Journal, 8(1), 289–317. https://doi.org/10.32614/RJ-2016-021.	N/A
fgsea_1.8.0	Korotkevich G, Sukhov V, Sergushichev A (2019). "Fast gene set enrichment analysis." bioRxiv. https://doi.org/10.1101/060012, http://biorxiv.org/content/early/2016/06/20/060012.	N/A
vcdExtra_0.7–1	https://cran.r-project.org/web/packages/vcdExtra/index.html	N/A
survminer_0.4.8	https://cran.r-project.org/web/packages/survminer/index.html	N/A
GSVA_1.30.0	Hänzelmann S, Castelo R, Guinney J (2013). "GSVA: gene set variation analysis for microarray and RNA-Seq data." BMC Bioinformatics, 14, 7. https://doi.org/10.1186/1471-2105-14-7, http://www.biomedcentral.com/1471-2105/14/7.	N/A

(*Continued on next page*)

CellPress
OPEN ACCESS

Continued

REAGENT or RESOURCE	SOURCE	IDENTIFIER
Hmisc_4.4–1	https://cran.r-project.org/web/packages/Hmisc/index.html	N/A
lumi_2.34.0	Du, P., Kibbe, W.A., Lin, S.M. (2008). "lumi: a pipeline for processing Illumina microarray." Bioinformatics	N/A
e1071_1.7–3	https://cran.r-project.org/web/packages/e1071/index.html	N/A
mlbench_2.1–1	https://cran.r-project.org/web/packages/mlbench/index.html	N/A
randomForest_4.6–14	https://cran.r-project.org/web/packages/randomForest/index.html	N/A
DoseFinding_0.9–17	https://cran.r-project.org/web/packages/DoseFinding/index.html	N/A
car_3.0–10	https://cran.r-project.org/web/packages/car/index.html	N/A
gplots_3.1.0	https://cran.r-project.org/web/packages/gplots/index.html	N/A
RColorBrewer_1.1–2	https://cran.r-project.org/web/packages/RColorBrewer/index.html	N/A
pheatmap_1.0.12	https://cran.r-project.org/web/packages/pheatmap/index.html	N/A
ggridges_0.5.2	https://cran.r-project.org/web/packages/ggridges/index.html	N/A
ggrepel_0.8.2	https://cran.r-project.org/web/packages/ggrepel/index.html	N/A
ggnewscale_0.4.3	https://cran.r-project.org/web/packages/ggnewscale/index.html	N/A
Gviz_1.26.5	Hahne F, Ivanek R (2016). "Statistical Genomics: Methods and Protocols." In Mathé E, Davis S (eds.), chapter Visualizing Genomic Data Using Gviz and Bioconductor, 335–351. Springer New York, New York, NY. ISBN 978-1-4939-3578-9, https://doi.org/10.1007/978-1-4939-3578-9_16, https://doi.org/10.1007/978-1-4939-3578-9_16.	N/A
patchwork_1.0.1	https://cran.r-project.org/web/packages/patchwork/index.html	N/A
SingleCell Experiment_1.10.1	Amezquita R, Lun A, Becht E, Carey V, Carpp L, Geistlinger L, Marini F, Rue-Albrecht K, Risso D, Soneson C, Waldron L, Pages H, Smith M, Huber W, Morgan M, Gottardo R, Hicks S (2020). "Orchestrating single-cell analysis with Bioconductor." Nature Methods, 17, 137–145. https://www.nature.com/articles/s41592-019-0654-x.	N/A
Seurat_4.0.1	Satija R, Farrell JA, Gennert D, Schier AF, Regev A (2015). "Spatial reconstruction of single-cell gene expression data." Nature Biotechnology, 33, 495-502. https://doi.org/10.1038/nbt.3192, https://doi.org/10.1038/nbt.3192.	N/A
bestNormalize_1.7.0	Peterson, 2021. "Finding Optimal Normalizing Transformations via bestNormalize." The R Journal, 13(1), 310–329. https://doi.org/10.32614/RJ-2021-041.	N/A
ggnewscale_0.4.5	https://cran.r-project.org/web/packages/ggnewscale/index.html	N/A
scales_1.1.1	https://cran.r-project.org/web/packages/scales/index.html	N/A
ggpattern_0.1.3	https://cran.r-project.org/web/packages/ggpattern/index.html	N/A
ggtern_3.3.0	Hamilton NE, Ferry M (2018). "ggtern: Ternary Diagrams Using ggplot2." Journal of Statistical Software, Code Snippets, 87(3), 1–17. https://doi.org/10.18637/jss.v087.c03.	N/A

(*Continued on next page*)

Continued

REAGENT or RESOURCE	SOURCE	IDENTIFIER
MASS_7.3–51.6	Venables and Ripley, 2002, Modern Applied Statistics with S, Fourth edition. Springer, New York. ISBN 0-387-95457-0, https://www.stats.ox.ac.uk/pub/MASS4/.	N/A
ggridges_0.5.3	https://cran.r-project.org/web/packages/ggridges/index.html	N/A
plotly_4.9.3	Sievert C (2020). Interactive Web-Based Data Visualization with R, plotly, and shiny. Chapman and Hall/CRC. ISBN 9781138331457, https://plotly-r.com.	N/A
ggplot2_3.3.3	Wickham H (2016). ggplot2: Elegant Graphics for Data Analysis. Springer-Verlag New York. ISBN 978-3-319-24277-4, https://ggplot2.tidyverse.org.	N/A
monocle3_0.2.2	Trapnell, C., Cacchiarelli, D., Grimsby, J. et al. The dynamics and regulators of cell fate decisions are revealed by pseudotemporal ordering of single cells. Nat Biotechnol 32, 381–386 (2014). https://doi.org/10.1038/nbt.2859	N/A
survMisc	https://cran.r-project.org/web/packages/survMisc/index.html	N/A

RESOURCE AVAILABILITY

Lead contact

Further information and request for resources and reagents should be directed to and will be fulfilled by the lead contact, Daniel Williamson (daniel.williamson@ncl.ac.uk).

Materials availability

This study did not generate new unique reagents.

Data and code availability

- Data arising from this study has been deposited in Array Express: E-MTAB-10754 and E-MTAB-10767 and are publicly available as of the date of publication. Additionally, this study makes use of previously deposited datasets GEO: GSE130051, GSE93646, and GSE119926. For scRNA-seq fetal cerebellar data, processed data are available through the Human Cell Atlas (https://www.covid19cellatlas.org/aldinger20) and the UCSC Cell Browser (https://cbl-dev.cells.ucsc.edu). Sequence data is available in the Database of Genotypes and Phenotypes, under accession number dbGAP: phs001908.v2.p1 (dbGAP/NCBI). Details are listed in the key resources table and method details section.
- No custom code was used in this study. Open-source algorithms were used as detailed in the method details section. Details on how these algorithms were used are available from the corresponding authors upon request.
- Any additional information required to reanalyze the data reported in this paper is available from the lead contact upon request.

EXPERIMENTAL MODEL AND SUBJECT DETAILS

Human tissue samples

Snap frozen tumor samples from individuals with a confirmed medulloblastoma diagnosis were used for RNA-seq analysis. These were provided as part of UK CCLG-approved biological study BS-2007–04 and/or with approval from Newcastle North Tyneside Research Ethics Committee (study reference 07/Q0905/71); informed, written consent was obtained from parents of all patients younger than 16 years. 66% of patients in the study were male, 15% were aged less than 3 years, 3% > 16 years and 82% aged 3-16 years (details given in Table S1).

METHOD DETAILS

Patient samples and study cohort

331 tumor samples from individuals with a confirmed medulloblastoma diagnosis were used for the RNA-seq analysis. Histopathological variants were defined according to the WHO 2016 guidelines (Louis et al., 2016). Metastatic status (M+) was defined as M > 1 as per Chang's criteria (Chang et al., 1969). *MYC* and *MYCN* amplification status was assessed by fluorescence *in situ* hybridization and/or copy-number estimates from methylation array and *TP53*, *CTNNB1*, and *TERT* mutation status by Sanger sequencing. DNA was

extracted using Qiagen DNeasy blood and tissue kit. Other mutations were assessed using next-generation sequencing. Whole-exome and targeted gene panel sequencing was performed using the Agilent SureSelect target enrichment platform and Illumina paired-end sequencing according to manufacturer's instructions. NGS datasets were analyzed for coding/exonic region variants using Genome Analysis Toolkit (GATK) version 3.7, according to Broad Institute's best practices (Burrows wheeler alignment, Haplotype Caller, Variant Quality Score Recalibration for exomes and Hard-filtering for panel) (Van der Auwera et al., 2013) and annotated using Ensembl Variant Effect Predictor (McLaren et al., 2016). Variants were predicted pathogenic if their consequence included coding or splice donor/acceptor mutations, max allele frequency was <0.01 in each of the large sequencing studies (ExAC, GnomAD/exomes, 1000 Genomes, ALFA) and predicted to be deleterious by both CAROL and FATHHM prediction tools (Lopes et al., 2012; Shihab et al., 2013). Variants called by targeted panel sequencing were called at a mean read depth of 278 (Standard Error of Mean = 11). Exome studies were performed at mean depth of 40x. Pathogenic variants required a variant allele frequency $\geq$10%, a minimum read depth $\geq$10 and a minimum 2 variant forward reads and 2 variant reverse reads. Variants were further curated for obvious artifacts by visual inspection in Integrative Genomics Viewer (IGV) (J. T. Robinson et al., 2011). Chromosome-arm level copy-number estimates were derived from DNA methylation array data using conumee (R/Bioconductor). A larger previously published MB_{Grp3}/MB_{Grp4} cohort (Sharma et al., 2019) (Schwalbe et al., 2017) (GSE130051 & GSE93646) to which 166 novel profiles were added (E-MTAB-10754) (n = 1670, exact samples used are detailed in Table S2) was used for methylation-only analysis.

RNA-seq analysis

Total RNA was extracted from snap frozen tissue samples using Trizol extraction followed by Qiagen RNeasy Cleanup Kit and then subjected to transcriptome sequencing using Illumina TruSeq RNA Library Prep and HiSeq 2500 platform achieving a ~90M paired end reads per sample. Following QC checks (fastqc/bamqc) samples were aligned to genome hg19 using *RNA-star* (Dobin et al., 2013) in two-pass alignment mode and per gene read counts generated using *ht-seq count* (Anders et al., 2015) and Gencode v25. Where isoform abundance estimates were required these were generated using *kallisto* (Bray et al., 2016). For differential expression analysis *DESeq2* (Love et al., 2014) (R/Bioconductor) was used. R/Bioconductor was used for other analysis, clustering and visualization. Read counts were first normalized and a variance stabilizing transform was first applied using the *vst* function within *DESeq2* (R/Bioconductor). Additionally, a batch correction controlling for sequencing batch was applied using the implementation of ComBat within the *sva* package (R/Bioconductor). Consensus NMF analysis was performed as per the method described in Schwalbe et al. (Schwalbe et al., 2017) and Sharma et al. (Sharma et al., 2019). Briefly, multi-run NMF is performed with n = 250 iterations of 80% bootstrapping. Metagenes calculated following each iteration are projected on to each removed sample and k-means clustering used to predict the class of each removed sample based on the larger training set. A range of NMF metagene ranks (3-10) and k-means clusters (3-10) are tested and cophenetic indices (a shorthand measure of the robustness of sample clustering) used to evaluate the consistency of classification for each combination of metagenes (Table S3). A 4-metagene/4-cluster solution was considered optimally stable based on the following rationale: i) for each level of NMF rank (k) average silhouette width dropped substantially after 4 clusters (c) and generally peaked at c = 4, ii) considering only c = 4 solutions, sample reproducibility was maximized by k = 4 and k = 5 metagenes with larger reductions in sample reproducibility for additional metagenes, iii) when choosing between k = 4 and k = 5 metagenes differences in sample reproducibility were minimal but the additional k5 metagene was effectively redundant, only expressed in a tiny minority of samples and did not track with any known biological characteristics or subgroups. Also 4-metagenes/4-clusters was coherent with previous descriptions of the disease and our prior understanding of the main subgroups. Samples which were assigned to the same class with <90% consistency upon resampling were designated as MB-NOS, except where they were alternately assigned as MB_{Grp3} or MB_{Grp4} with >90% consistency, in which case they were classified as MB_{Grp3}/MB_{Grp4}.

Averaged and standardized metagene h-values from across the bootstraps were used as measures of metagene expression. All NMF projections were performed using column-rank and post-projection normalization as per the method described by Tamayo et al. (Tamayo et al., 2007). t-SNE were used for visualization was performed using the *Rtsne* package (R/CRAN).

G3/G4 score was calculated by applying a logistic transformation 1/(1 + exp(-x)) to the MB_{Grp3} and MB_{Grp4} metagenes (excluding two outliers). The G3/G4 score was calculated as the MB_{Grp3} proportion of the total metagene scaled to between 0 and 1. For convenience of visualization, or where categorical comparison was required, we referred to individuals >0 & $\leq$0.2 as "HighG4", >0.2 & $\leq$ 0.4 as "LowG4", >0.4 & $\leq$0.6 as "G3.5", >0.6 & $\leq$0.8 as "LowG3" and >0.8 & $\leq$1 as "HighG3".

RNA editing was estimated using the QEdit/Reditools pipeline as previously described (https://github.com/BioinfoUNIBA/QEdit) (Lo Giudice et al., 2020). Differential RNA-editing was calculated using a p-adjusted (Benjamini-Hochberg) Mann-Whitney U-test for two group analysis and Anova with TukeyHSD (post-hoc) for multi-group analysis. Where unknown from DNA analysis *GFI1/GFI1B*, *PRDM6* rearrangements were each inferred from RNA-seq data as per the method used originally by Northcott et al. (Northcott et al., 2014, 2017).

GSEA was performed using MsigDb library version 7.1 and the implementation of the original algorithm within the package *fgsea* (R/Bioconductor) and ssGSEA using the implementation within *GSVA* (R/Bioconductor) (Hänzelmann et al., 2013). The following gene sets were selected as reflective of the pathway categories given in Figure 4C. MYC = "HALLMARK_MYC_TARGETS_V2", "MYC_UP.V1_UP", "DANG_MYC_TARGETS_UP". Cell Cycle = "FISCHER_G1_S_CELL_CYCLE", "GO_POSITIVE_REGULATION_OF_CELL_CYCLE", "GO_SIG NAL_TRANSDUCTION_INVOLVED_IN_CELL_CYCLE_CHECKPOINT", TP53 = "CEBALL OS_TARGETS_OF_TP53_AND_MYC_UP", "REACTOME_TRANSCRIPTIONAL_REGULATION _ BY_TP53", "REACTOME_TP53_REGULATES", MTOR = "HALLMARK_MTORC1_SIGNALING", "MTOR_UP.V1_UP", "MTOR_UP.

N4.V1_UP", PHOTORECEPTOR = "GO_EYE_PHOTOREC EPTOR_CELL_DIFFERENTIATION", "GO_CAMERA_TYPE_EYE_PHOTORECEPTOR_CELL_ DIFFERENTIATION", "GO_EYE_PHOTORECEPTOR_CELL_DEVELOPMENT", TGFB1 = "KARLSSON_TGFB1_TARGETS_UP", "JAZAG_TGFB1_SIGNALING_VIA_SMAD4_UP", "KARAKAS_ TGFB1_SIGNALING" NOTCH = "GO_POSITIVE_REGULATION_OF_NOTCH_SIGNALING _PATHWAY", "REACTOME_ACTIVATED_NOTCH1_TRANSMITS_SIGNAL_TO_THE_NUCLEUS", "NGUYEN_NOTCH1_TARGETS_UP", Neuronal Diff = "GO_CENTRAL_NERVOUS_SYSTEM _NEURON_DIFFERENTIATION" "LE_NEURONAL_DIFFERENTIATION_UP".

In analyzing association with G3/G4 score, the loss or gain of each non-acrocentric chromosome arm was considered as were the more frequent MB_{Grp3}/MB_{Grp4} mutations in genes *ATM*, *CTDNEP1*, *KDM6A*, *KIF26B*, *KMT2C*, *KMT2D*, *NBAS*, *NEB*, *RYR3*, *SMARCA4*, *SPTB*, *TBR1*, *TSC2*, and *ZMYM3*.

DNA METHYLATION ANALYSIS

Beta/M-values were derived from HumanMethylation450 BeadChip (450k) and Infinium HumanMethylationEPIC (850k) arrays using the ssNOOB method within the package *minfi* (Aryee et al., 2014) excluding known SNPs and cross-hybridizing probes. In order to construct a random forest classifier which predicted G3/G4 score from DNA methylation data, we performed feature selection of CpGs using 192 MB_{Grp3}/MB_{Grp4} samples with both RNA-seq (i.e. known G3/G4 score) and Methylation array. We constructed using *limma* (R/Bioconductor) a number of bootstrapped (80% with 100 iterations) significance tests testing differential methylation between each of the categories HighG4, LowG4, G3.5, LowG3 and HighG3. We measured average performance for a range of numbers of features (10-100) on removed samples using a tuned support vector machine, however performance plateaued after a certain number of features, so it was decided to select the top 80 most frequently selected CpGs for each comparison. Thus n = 400 CpG features were used to train a random forest classifier which was then subject to recursive feature elimination using 50x cross-validation and implemented using the *rfe/rfeControl* function within the *caret* package (R/CRAN). An internal validation process by which model performance was estimated by recreating the model multiple times without individuals whose predicted score was then used to estimate performance. Where sigmoid curves are shown, these were fitted using the *fitmod* function within the *Dose-Finding* package (R/Bioconductor). For visualization these were scaled to a minimum 0 and maximum 1.

Methylation subtype calling (Sharma et al., 2019) was obtained using an extension of the Heidelberg brain tumor classifier available at [https://www.molecularneuropathology.org/mnp]. A methylation classifier prediction score of >0.8 was used to assign subtype. Samples were excluded if not confirmed as MB by MNP.

Significantly differentially methylated regions (DMRs) distinguishing G4High, G4Low, G3.5, G3Low and G3High were calculated using *dmrcate* (R/Bioconductor) using settings lambda = 1000, C = 2. Regions were considered when the total number of CpGs $\geq$5, the minimum FDR <0.05 and the mean Beta fold change >0.25. These were further filtered to identify DMRs which overlapped with the MB_{Grp3}/MB_{Grp4} specific enhancer/superenhancer regions identified by Lin et al. (Lin et al., 2016).

scRNA-SEQ ANALYSIS

A previously published medulloblastoma scRNA-seq dataset (Hovestadt et al., 2019) GSE119926 was used. However, we used only the MB_{Grp3}/MB_{Grp4} primary patient samples (excluding the patient-derived xenografts) (n = 4256 cells, n = 15 samples) and excluded patients SJ970 and SJ723 due to the relatively few available cells. The pre-publication Human fetal cerebellar single cell reference dataset, consisting of 69,174 cells, classified into 21 cell types and derived from 15 donors between 9 and 21 PCW, details can be found within https://www.biorxiv.org/content/10.1101/2020.06.30.174391v1 (Aldinger et al. in press Nature Neuroscience). For the purposes of metagene projection, *Seurat* (R/Bioconductor) (Butler et al., 2018) was used to select the 5000 most variable features using the "vst" method for both datasets and the resulting normalized matrices subject to NMF projection of the bulk metagenes and calculation of the G3/G4 score as per the bulk analysis described above. In this way, a per-cell metagene score and G3/G4 score was calculated.

An alternative method was used for validation purposes, namely Canonical Correlation Analysis (CCA). CCA has been previously used to facilitate cross-species/cross-platform comparisons (Butler et al., 2018). The limitations of CCA are such that it cannot be used to achieve quite the same cell by cell projection we can with NMF. Nevertheless, the basic results are comparable showing similarity between MB_{WNT} and RL, MB_{SHH} and GCP, MB_{Grp3} and RL and MB_{Grp4} with eCN/UBC.

CCA is performed as a singular value decomposition of a distance matrix between bulk RNA-seq medulloblastoma and the fetal cerebellar scRNA-seq dataset. Cosine distance is used to calculate a CCA score reflecting the correlation of differential expression and thus the relative similarity between medulloblastoma subgroup and fetal cerebellar cell type; a similar technique was used by Hovestadt et al. (Hovestadt et al., 2019). Whilst the top similarity for MB_{Grp3} and MB_{Grp4} is RL and eCN/UBC respectively treating them as discrete subtypes - although necessary for the CCA analysis - goes somewhat against our purpose. We therefore created a second analysis where we divided the $MB_{Grp3/Grp4}$ patients in our bulk reference into five quantiles based on their G3/G4 score, i.e. position on the continuum. This reflected the transition from a "straight-up" resemblance to eCN/UBC at the extreme Grp4 end of the continuum and RL at the extreme Grp3 end.

Developmental trajectory analysis was performed using *monocle v3* (Qiu et al., 2017) (R/Bioconductor) using 12,243 cells classified as RL, GCP, GN or eCN/UBC which we defined broadly as the rhombic lip lineage as per Aldinger et al. Monocle v3 functions used

were preprocess_cds, align_cds, reduce_dimension, cluster_cells, learn_graph, order_cells and plot_cells to visualize by UMAP. To rule out the possibility that an association between MB_{Grp3} and RL was simply an artifact of higher cellular proliferation we estimated the cell cycle phase using Seurat. Whilst there is a higher proportion of cycling cells in RL compared to eCN/UBC the same can also be said of GCP. This speaks against a default matching of MB_{Grp3} metagenes to any actively cycling cells. We also tried regressing out the effect of the cell cycle using the ‘‘CC.difference’’ (Seurat, R/Cran) method and reprojected our metagenes. This had little effect on the projection, as did removing all genes with ‘‘cell cycle’’ ontology. Top genes driving association with projected MB_{Grp3} and MB_{Grp4} metagenes in the developmental setting include *ASIC2*, *GRIK1*, *KCNQ3*, *ANK3*, *ANKS1B*, *GRIA2*; none of which are classic oncogenes, The GO terms significantly enriched (DAVID/EASE) are ‘‘cell junction’’, ‘‘postsynaptic membrane’’, ‘‘integral component of plasma membrane’’ and ‘‘cell division’’ (each Benjamini $p < 0.01$)

The relevant branches for MB_{Grp3}/MB_{Grp4} and MB_{SHH} were divided as indicated (Figure 7) and the relationship between pseudotime and G3/G4 score/metagene was defined using a loess curve function. This enabled developmental and oncogenic events to be mapped onto a common scale (Figure 7). Genes whose expression varied significantly according to pseudotime were detected using Moran's test statistic as implemented by *monocle v3*. For analysis of the differences between $MB_{SHH\text{-}Infant}$ and $MB_{SHH\text{-}Child,}$ a further metagene calculated using NMF rank = 2 only on MB_{SHH} (67/331 samples) was additionally projected onto the single cells in the same manner as the other metagenes. For calculating empirical density, the *density* function was used (R/Bioconductor) except where weighted two-dimensional estimation was needed in which case the *kde2d.weighted* function from the package *ggtern* (R/Bioconductor) was used. Weights were calculated as the number of cells at a given sampling point (9-21PCW) as a proportion of the total number of cells sampled.

QUANTIFICATION AND STATISTICAL ANALYSIS

Data analysis and visualization was carried out in R 3.5.3 except for the analysis of fetal cerebellar scRNA-seq which was performed using R 4.0.2. CRAN and Bioconductor packages used are given in the key resources table. To test significant association with time to death/progression, a log-rank test (test for trend as implemented by *survMisc* (R/Cran)) or Cox-regression was used. OS was used when assessing the basic relationship between G3/G4 score/subtype with risk of death. This was to maximise the number of data points (more OS than PFS data was available). When assessing use as an independent biomarker PFS was preferred as standard for the field as patients who relapse, almost without fail, go on to relapse.

A Kolmogorov-Smirnoff test was used to compare distributions across the G3/G4 continuum of patients with or without specific clinico-pathological mutational and copy number features. Where significant this indicates that patients with or without a given feature are significantly likely to be drawn from different G3/G4 score distributions. The implication being that with respect to a given feature patients are non-randomly distributed across the G3/G4 continuum. Where gene expression/pathway associations with G3/G4 score are assessed, these are assessed using Pearson's correlation coefficient (Table S4). The test statistics and significant p-values ($p < 0.05$) are stated in the text and figures and were adjusted for multiple hypothesis testing using Benjamini-Hochberg for high-dimensional analyses. Where values of n are given, these generally pertain to number of samples/individual patients except where otherwise indicated. Boxplots, where used, show dispersion as per standard i.e. (center line = median, box = interquartile range, whisker = range minus outliers).

Data were excluded where samples were clearly indicated to be duplicated across multiple related datasets. Additional exclusions were carried out for samples where methylation array detection p value did not reach significance threshold in at least 90% of the array. Methylation samples were excluded from the analysis if not confirmed as medulloblastoma by MNP2.0. In our analysis of the scRNA-seq dataset GSE119926 we excluded patients SJ970 and SJ723 due to the relatively few available cells.

Cell Reports, Volume *40*

Supplemental information

Medulloblastoma group 3 and 4 tumors comprise a clinically and biologically significant expression continuum reflecting human cerebellar development

Daniel Williamson, Edward C. Schwalbe, Debbie Hicks, Kimberly A. Aldinger, Janet C. Lindsey, Stephen Crosier, Stacey Richardson, Jack Goddard, Rebecca M. Hill, Jemma Castle, Yura Grabovska, James Hacking, Barry Pizer, Stephen B. Wharton, Thomas S. Jacques, Abhijit Joshi, Simon Bailey, and Steven C. Clifford

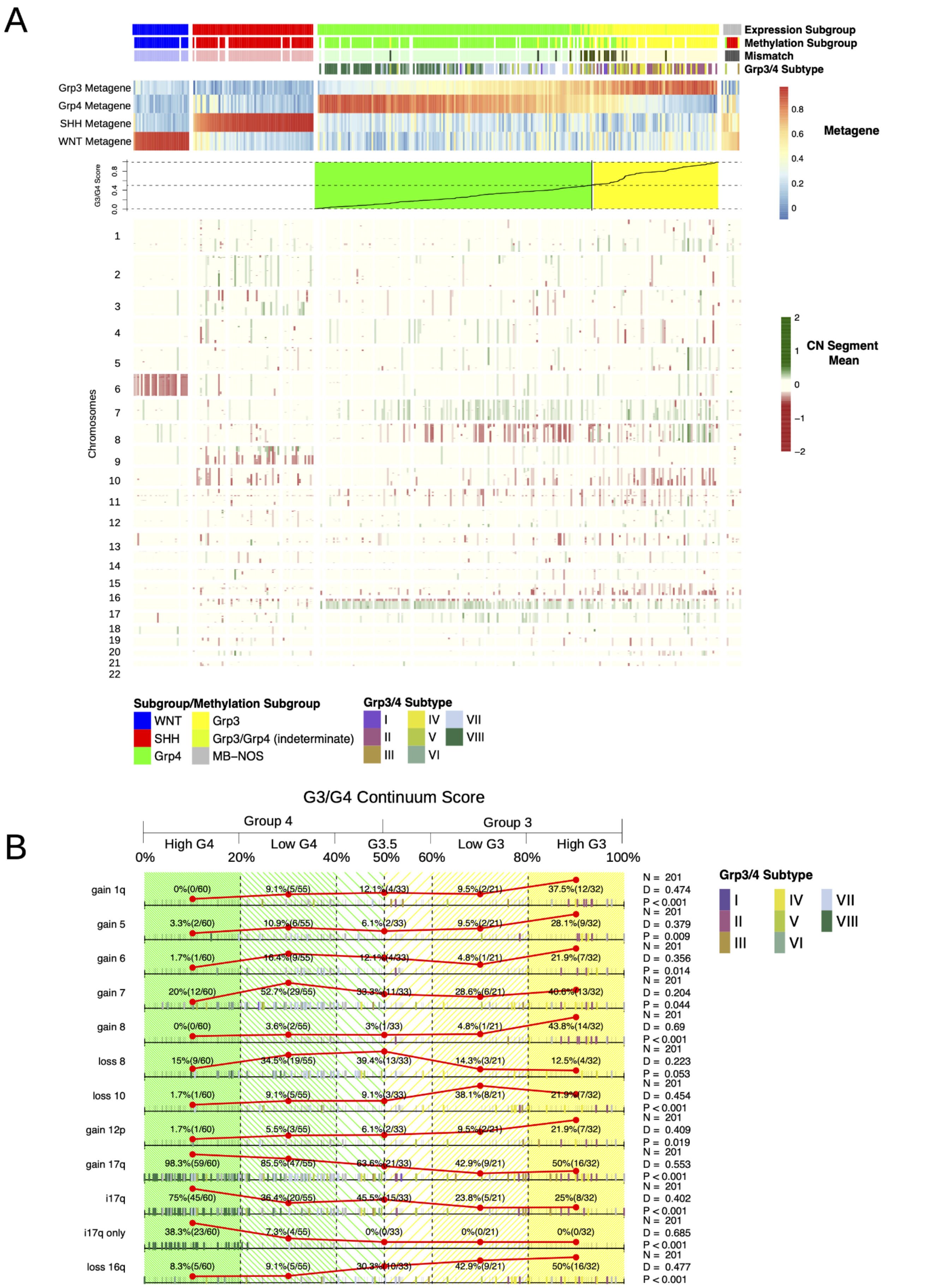

Figure 1S: Copy number changes are non-randomly distributed with respect to position on the Group3/Group4 continuum. A: Heatmap showing copy number changes by chromosome for n=331 MB and grouped by subgroup. MB_{Grp3}/MB_{Grp4} individuals are ordered by G3/G4 score. Column annotation shows subgroup as determined by RNA-seq (Expression Subgroup) as determined by DNA methylation array (Methylation Subgroup), and DNA methylation MB_{Grp3}/MB_{Grp4} subtype (I-VIII) as per Sharma *et al* 2019 (Sharma et al., 2019) as defined using MNPv2 classifier (Capper et al., 2018) (Grp3/4 Subtype). The line plot (bottom) shows G3/G4 score. **B:** Rug plot showing distribution of aneuploidy/copy number change with respect to G3/G4 score. Summary counts are given according to the convenient divisions of HighG4, LowG4, G3.5, LowG3,

HighG3 (these categories are arbitrary divisions of the continuum for the purposes of visualization and comparison and do not represent “real” subgroups) and reflected by the red line plots. Presence of a given feature is indicated by a bold tick mark, the color of which indicates methylation MB_{Grp3}/MB_{Grp4} subtype (I-VIII). Adjusted P-values for a Kolmogorov-Smirnoff statistic (D) are shown to denote non-random distribution of features with respect to G3/G4 score. Related to Figure 2.

A

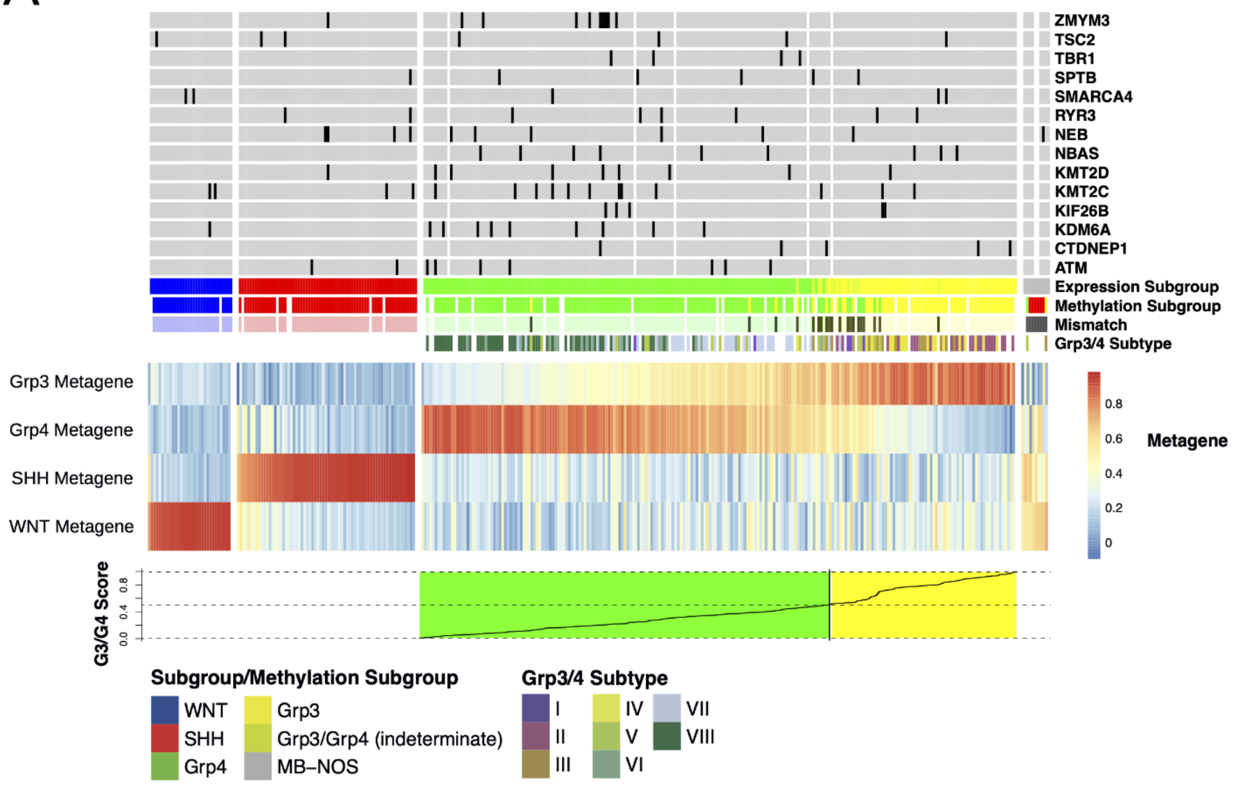

B

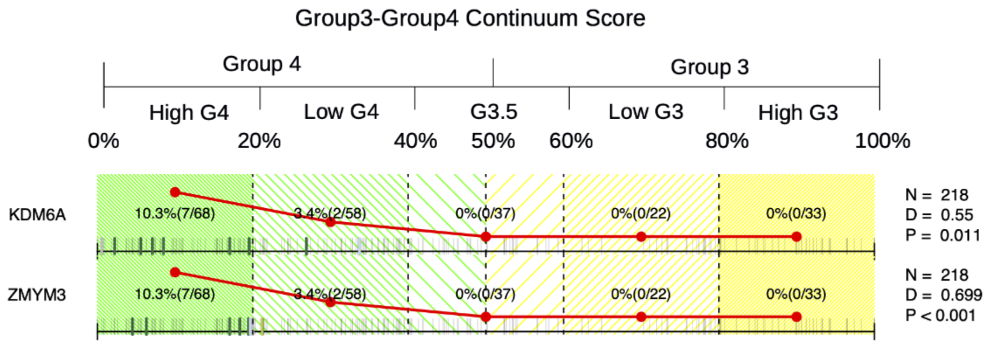

Figure 2S: Certain mutations are non-randomly distributed with respect to the Group3/Group4 continuum. A: Heatmap showing 4 consensus NMF metagenes calculated for n=331 MB and grouped by subgroup. MB_{Grp3}/MB_{Grp4} individuals are ordered by G3/G4 score. Column annotation shows subgroup as determined by RNA-seq (Expression Subgroup) as determined by methylation (Methylation Subgroup), methylation MB_{Grp3}/MB_{Grp4} subtype (I-VIII) as per Sharma *et al* 2019 as defined using MNPv2 classifier (Grp3/4 Subtype). Presence of mutations are indicated to be present or not by dark grey shading. White indicates missing data. **B:** Rug plot showing distribution of mutations with respect to G3/G4 score. Summary counts are given according to the convenient divisions of HighG4, LowG4, G3.5, LowG3, HighG3 (these categories are arbitrary divisions of the continuum for the purposes of visualization and comparison and do not represent "real" subgroups) and reflected by the red line plots. Presence of a given feature is indicated by a bold tick mark the color of which indicates methylation MB_{Grp3}/MB_{Grp4} subtype (I-VIII). P-values for a Kolmogorov-Smirnoff statistic (D) are shown to denote non-random distribution of features with respect to G3/G4 score. Related to Figures 1 & 2.

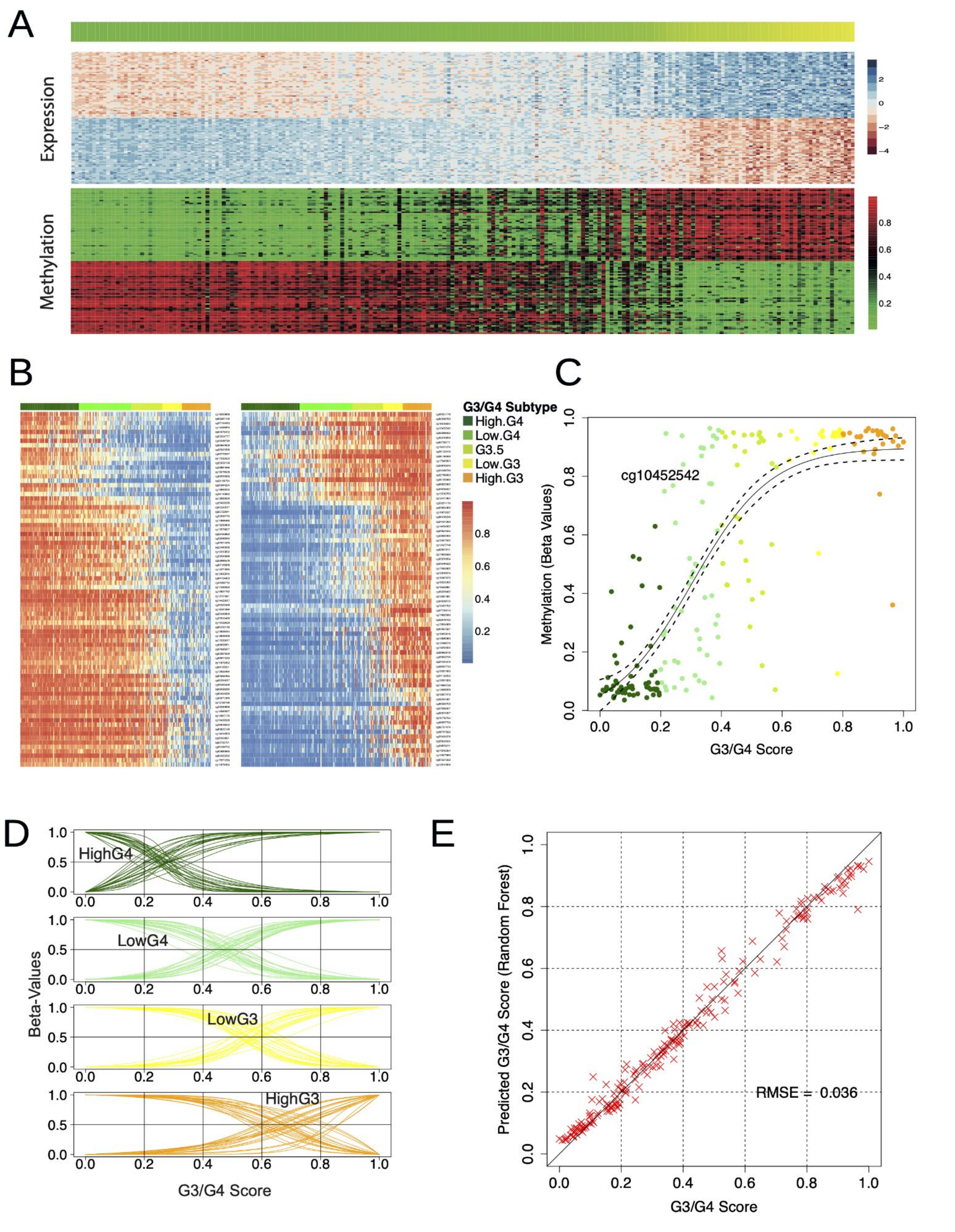

Figure 3S: The transcriptional G3/G4 score can be recapitulated using DNA methylation profiles. A: Heatmap showing top 50 genes most significantly differentially expressed (top) and top 50 CpGs differentially methylated between MB_{Grp3} and MB_{Grp4}. Samples are ordered according to G3/G4 score. Note the difference in gradation for the expression values as opposed to the more binary distribution of DNA methylation beta-values. **B:** Heatmap showing DNA methylation values of the top 40 most discriminatory CpGs distinguishing HighG4 (dark green), LowG4 (light green), Low G3 (yellow) and High G3 (orange). G4 hypermethylated CpGs are shown on the left and hypomethylated CpGs on the right. Samples are ordered according to G3/G4 score and G3/G4 categories (HighG4, LowG4, G3.5, LowG3, HighG3; these categories are arbitrary divisions of the continuum for the purposes of visualization and comparison and do not represent "real" subgroups) are annotated. **C:** Scatterplot showing beta-values for CpG "cg19784198" colored by G3/G4 categories (HighG4, LowG4, G3.5, LowG3, HighG3) an example of a CpG

differentially expressed between MB_{Grp3} and MB_{Grp4} showing a bimodal methylation distribution. The relationship with G3/G4 score can effectively be modelled by a sigmoid/logistic function. **D:** Fitted sigmoid curve representing the relationship between CpG beta-value and G3/G4 Score. Top 40 most discriminatory CpGs distinguishing HighG4 (dark green), LowG4 (light green), Low G3 (yellow) and High G3 (orange) are shown. **E:** The performance of the cross-validated random forest classifier showing predicted G3/G4 score (derived from DNA methylation values) against actual G3/G4 score (derived from RNA-seq) n = 192. Related to Figure 2.

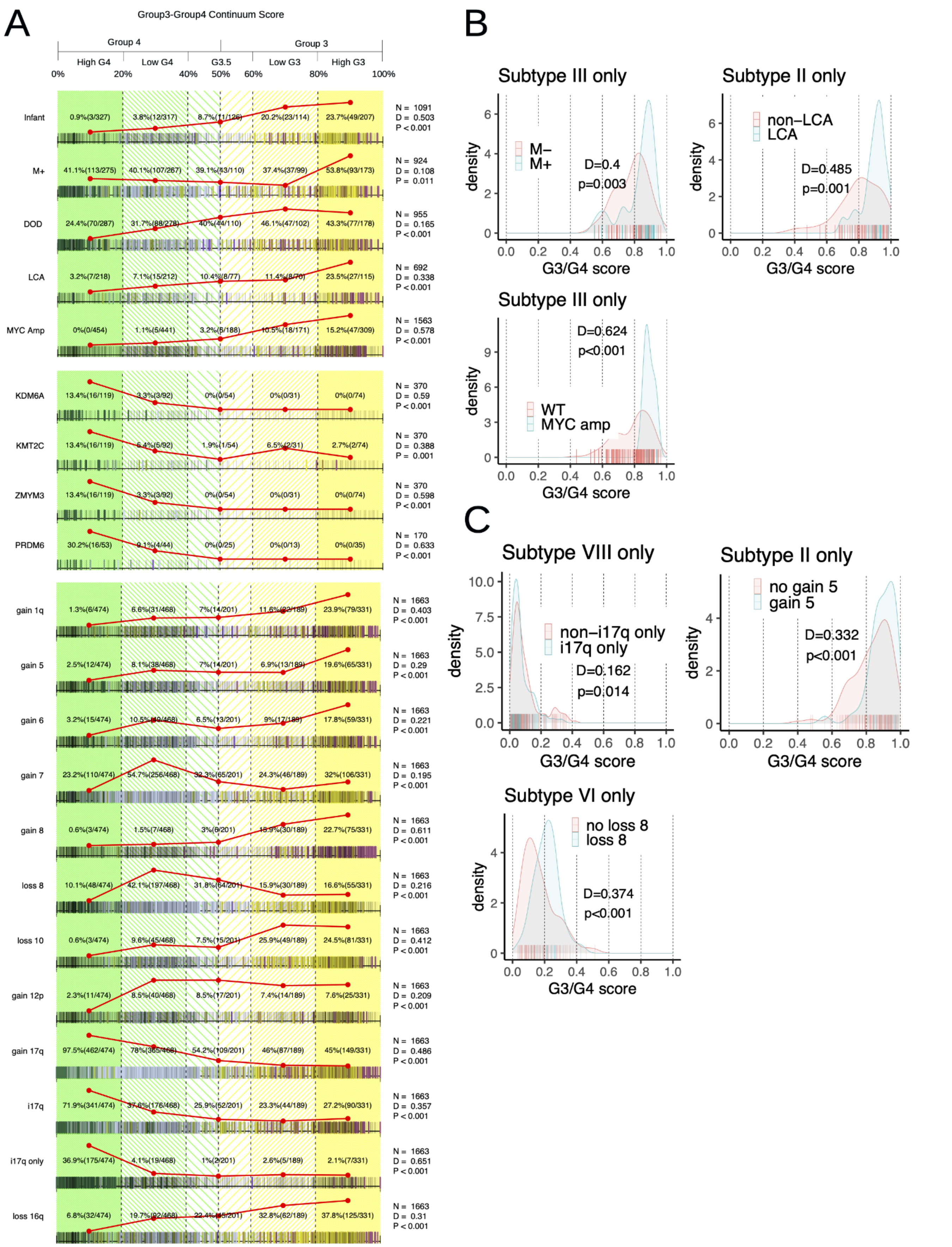

Figure 4S Clinico-pathological characteristics, mutations and copy number changes are all non-randomly distributed with respect to the Group3/Group4 continuum (as determined by DNA-methylation profile). A: Rug plot showing distribution of clinicopath features (top) mutations (middle) and copy number (bottom) with respect to G3/G4 score derived from DNA methylation data. Summary counts are given according to the convenient divisions of HighG4, LowG4, G3.5, LowG3, HighG3 (these categories are arbitrary divisions of the continuum for the purposes of visualization and comparison and do not represent "real" subgroups) and reflected by the red line plots. Presence of a given feature is indicated by a bold tick mark the color of which indicates methylation MB_{Grp3}/MB_{Grp4} subtype (I-VIII). P-values for a Kolmogorov-Smirnoff statistic (D) are shown to denote non-random distribution of features with respect to G3/G4 score. Infant=age at diagnosis < 3 years, Metastases = M+, DOD=Dead of Disease, LCA = Large Cell Anaplasia, PRDM6 = PRDM6 rearrangement. **B:** Empirical density and rug plots

showing the distribution of M+ in MB_{Grp3}/MB_{Grp4} subtype III, LCA in MB_{Grp3}/MB_{Grp4} subtype II and MYC amplification in MB_{Grp3}/MB_{Grp4} subtype III with respect to G3/G4 score. The given clinico-pathological features are significantly non randomly distributed with respect to G3/G4 score even within specific MB_{Grp3}/MB_{Grp4} subtypes as shown by Kolmogorov-Smirnoff test (D). **C:** Empirical density and rug plots showing the distribution of copy number changes i17q in MB_{Grp3}/MB_{Grp4} subtype VIII, Gain of chromosome 5 in MB_{Grp3}/MB_{Grp4} subtype II and loss of chromosome 8 in MB_{Grp3}/MB_{Grp4} subtype VI with respect to G3/G4 score. The given copy number features are significantly non randomly distributed with respect to G3/G4 score even within specific MB_{Grp3}/MB_{Grp4} subtypes as shown by Kolmogorov-Smirnoff test (D). Related to Figure 2.

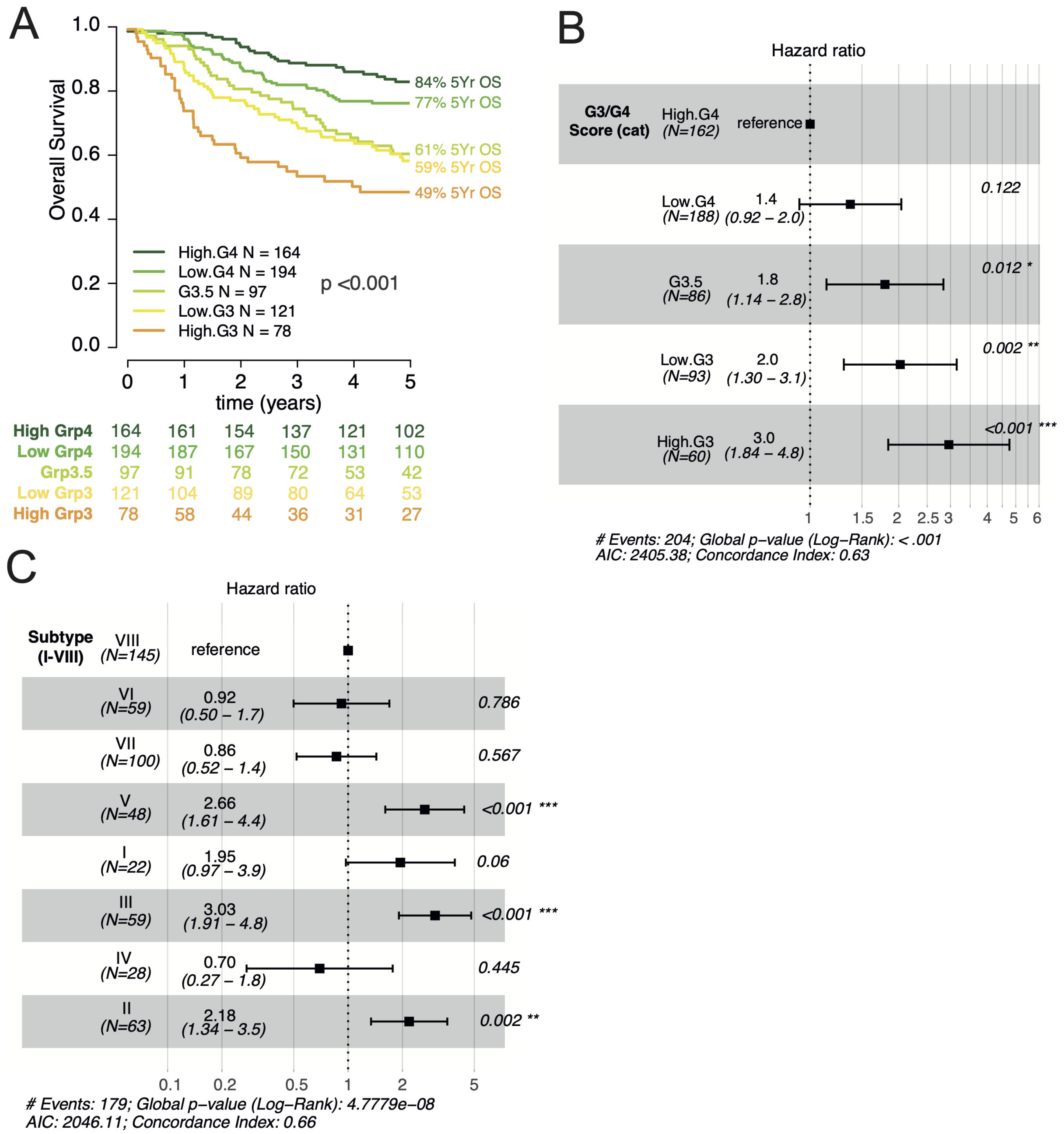

Figure 5S Survival outcomes of Group3/Group4 medulloblastoma patients is significantly related to position on the Group3/Group4 continuum (as determined by DNA-methylation profile). A: Kaplan-Meier plot showing significant differences in MB_{Grp3}/MB_{Grp4} overall survival (patients of all ages) by G3/G4 continuum position divided for convenience as HighG4, LowG4, G3.5, LowG3, HighG3 (these categories are arbitrary divisions of the continuum for the purposes of visualization and comparison and do not represent "real" subgroups). **B:** Forest plot showing univariate Cox models (patients > 3 years) of overall survival containing the variables G3/G4 score (as predicted by DNA methylation) treated as a categorical variable and **C:** MB_{Grp3}/MB_{Grp4} methylation subtype. Related to Figure 2.

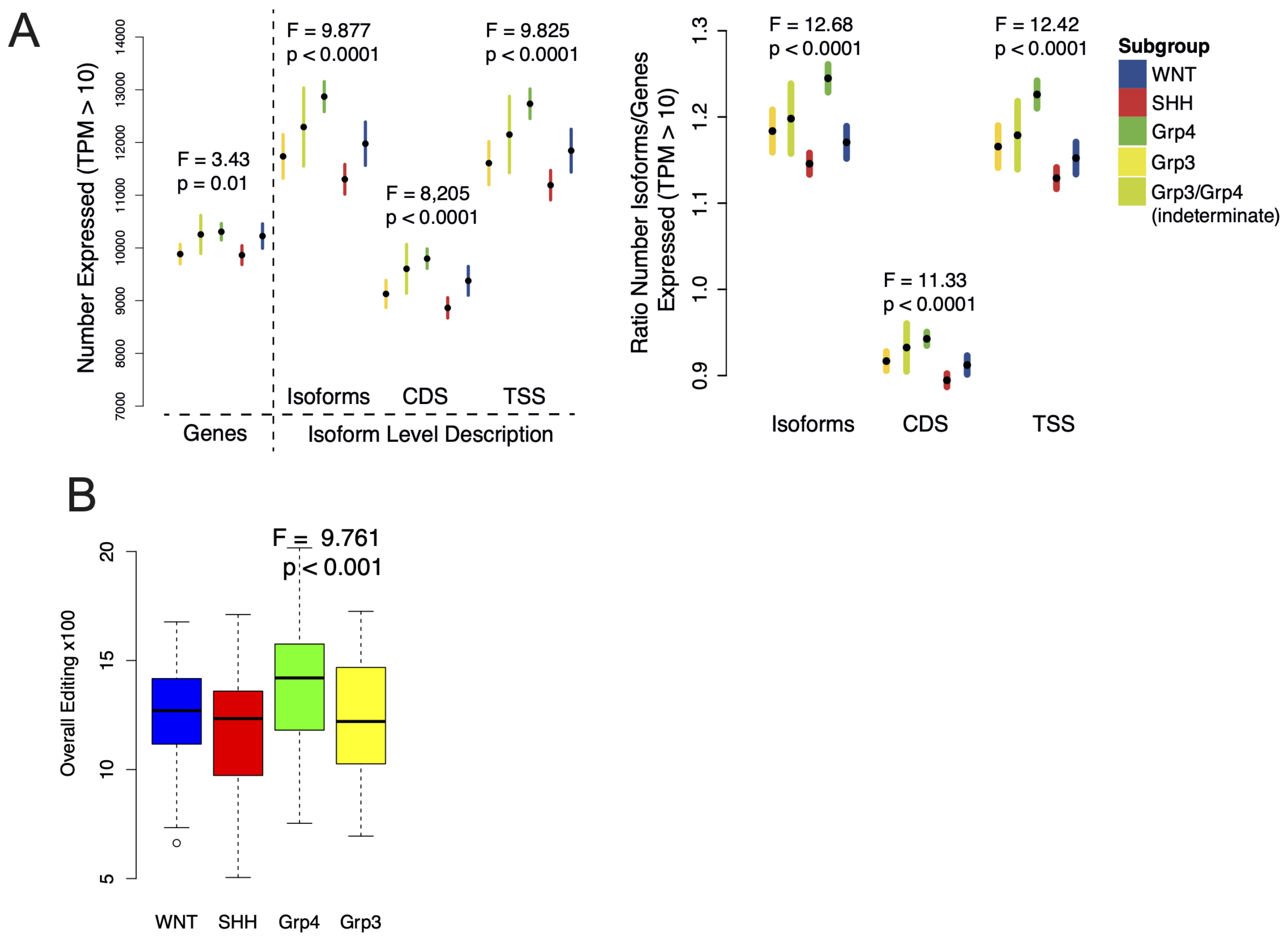

Figure 6S Isoform diversity and level of RNA-editing is related to medulloblastoma subgroup. A: Boxplot showing (left) the distribution by MB subgroup of moderately expressed genes, isoforms, CDS or TSS as defined by a TPM>10 and (right) the same given as a ratio of expressed isoforms, CDS or TSS per expressed genes. **B:** Boxplot showing significant differences in OEI (Overall Editing Index), i.e. level of RNA-editing by MB subgroup. Related to Figure 4.

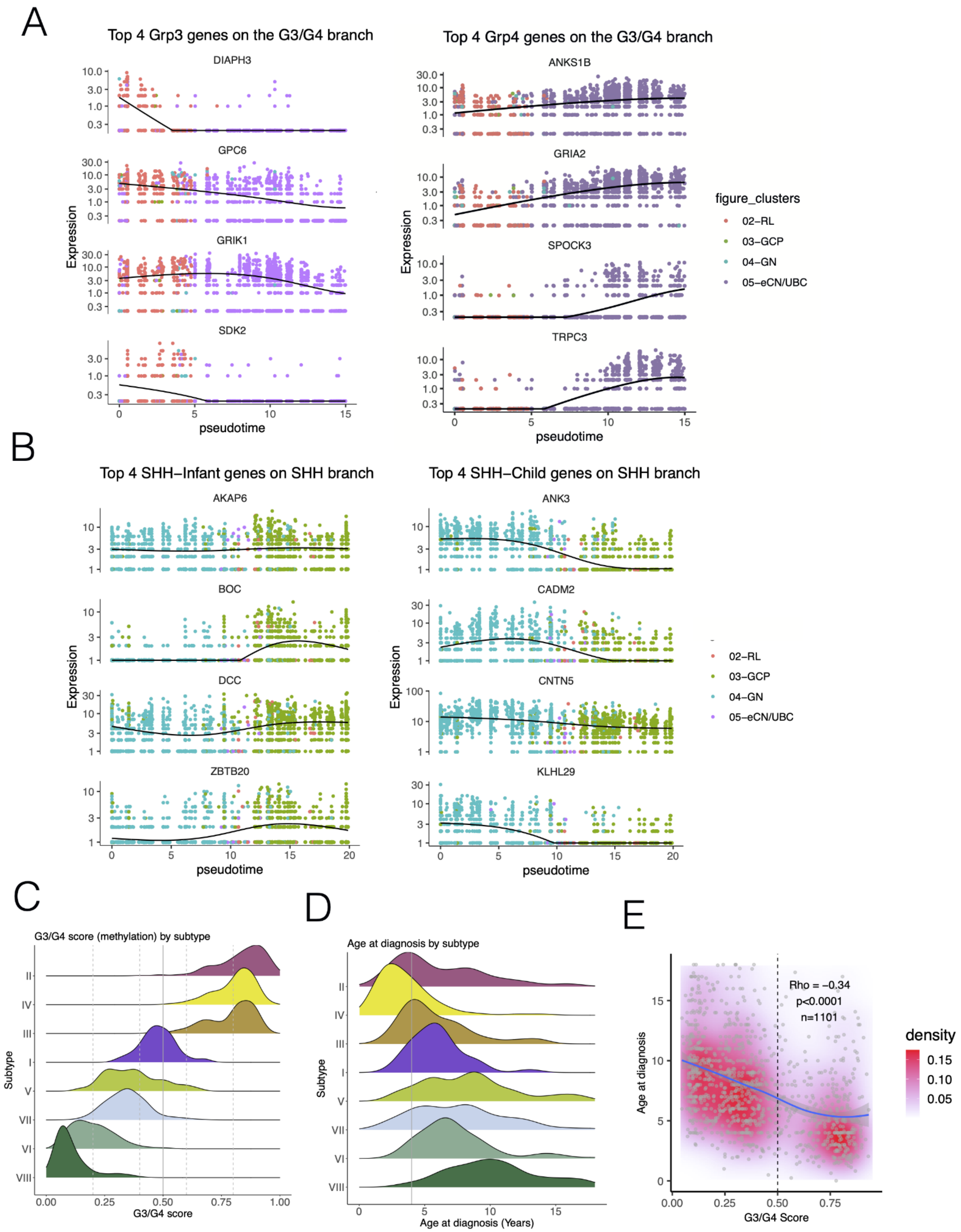

Figure 7S: Expression of Group3/Group4 genes is significantly related to (earlier/later) pseudotime position along the Rhombic Lip – Unipolar Brush cell trajectory and position on the continuum is related to age of onset. Plots showing the per-cell expression of genes whose expression varies according to pseudotime on the **A:** RL to eCN/UBC branch (MB_{Grp3} specific genes are shown on the left and MB_{Grp4} specific genes shown on the right) and the **B:** GCP to GN branch ($MB_{SHH\text{-}Infant}$ specific genes are show on the left and $MB_{SHH\text{-}Child}$ specific genes are shown on the right). Cell type is denoted by color. Black line represents a loess curve. Expression is represented as normalized count data. **C:** Ridgeplots showing distribution of G3/G4 score MB_{Grp3}/MB_{Grp4} patients by methylation subtype (I-VIII) and **D:** distribution of age at diagnosis by DNA methylation subtype (I-

VIII). **E:** Scatterplot showing age at diagnosis by G3/G4 score (as determined by DNA methylation), 2d empirical density is shown as red shading and a loess curve with 95% CI is shown as blue line with grey shading. Related to Figure 6 & Figure 7.
